# Chemoenzymatic Synthesis of Asymmetrically Branched Human Milk Oligosaccharide Lacto-*N*-Hexaose

**DOI:** 10.3389/fchem.2022.905105

**Published:** 2022-05-31

**Authors:** Kai-Eng Ooi, Xiu-Wen Zhang, Cheng-Yu Kuo, Ying-Jia Liu, Ching-Ching Yu

**Affiliations:** ^1^ Department of Chemistry and Biochemistry, National Chung Cheng University, Chiayi, Taiwan; ^2^ Institute of Biological Chemistry, Academia Sinica, Taipei, Taiwan

**Keywords:** human milk oligosaccharides, fucosylation, enzymatic synthesis, selective glycosylation, lacto-*N*-hexaose

## Abstract

We herein reported the first chemoenzymatic synthesis of lacto-*N*-hexaose (LNH) by combining chemical carbohydrate synthesis with a selectively enzymatic glycosylation strategy. A tetrasaccharide core structure GlcNH_2_β1→3 (GlcNAcβ1→6) Galβ1→4Glc, a key precursor for subsequent enzymatic glycan extension toward asymmetrically branched human milk oligosaccharides, was synthesized in this work. When the order of galactosyltransferase-catalyzed reactions was appropriately arranged, the β1,4-galactosyl and β1,3-galactosyl moieties could be sequentially assembled on the C6-arm and C3-arm of the tetrasaccharide, respectively, to achieve an efficient LNH synthesis. Lacto-*N*-neotetraose (LN*n*H), another common human milk oligosaccharide, was also synthesized en route to the target LNH.

## Introduction

Human milk oligosaccharides (HMOs) are a group of structurally complex glycans that are highly abundant in breast milk and are associated with various biological events in the human body (Chen et al., 2015; Cheng et al., 2021; Chutipongtanate et al., 2022). Several mechanisms of HMOs that provide health benefits, including intestinal microbiome regulation, epithelial and immune cell response modulation, prevention of viral infection, and the provision of nutrients to the brain, have been examined (Bode, 2012; Kulinich and Liu, 2016; Kirmiz et al., 2018; Fabiano et al., 2021; Moore et al., 2021; Morrin et al., 2021; Rousseaux et al., 2021). Due to advances in the structural analysis of HMOs by mass spectroscopy, most of the HMO scaffolds and linkages have been documented and studied (Ruhaak and Lebrilla, 2012; Bao et al., 2013; Mernie et al., 2019; Tsai et al., 2019a). All HMO structures contain lactose at the reducing end, which can be elongated through the addition of β1,3-linked or β1,6-linked lacto-*N*-biose (Galβ1,3-GlcNAc; type-1 LNB) or *N*-acetyllactosamine (Galβ1,4GlcNAc; type-2 LacNAc). Lactose elongation through the β1,3-linkage leads to linear structures that are *para*-HMOs. By contrast, the β1,6-linkage introduces chain branching, which leads to the formation of *iso*-HMOs (Kobata, 2010). Moreover, the LacNAc extension can be further decorated with fucosyl or sialyl moieties, which greatly expands the structural diversity of HMOs (>200 discovered in nature) (Remoroza et al., 2018; Xu and Townsend, 2021). To create adequate quantities of structurally defined compounds for biological studies, researchers have developed various reliable synthesis methods for HMO production that use whole-cell fermentation (Faijes et al., 2019; Lu et al., 2021), chemical (Craft and Townsend, 2017; Bandara et al., 2019a; Bandara et al., 2019b; Bandara et al., 2020), and enzymatic methods (Zhao et al., 2016; McArthur et al., 2019; Zeuner and Meyer, 2020).

**FIGURE 1:**
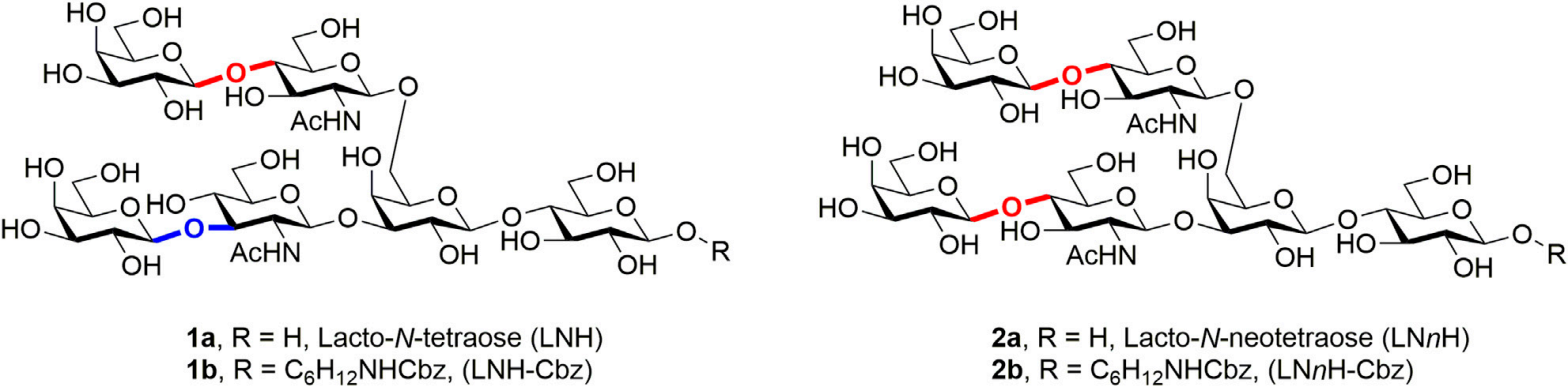
Structure of LNH and LN*n*H.

The branched hexasaccharides lacto-*N*-hexaose (LNH **1a**) and lacto-*N*-neohexaose (LN*n*H **2a**) are structurally derived from lacto-*N*-tetraose and lacto-*N*-neotetraose, respectively, and are two of the representative HMO core structures (Figure 1). Because exploring the role of branched HMOs in various biological processes and applications in glycobiology requires sufficient quantities of pure oligosaccharide samples, establishing efficient synthesis methods for more complicated branched HMOs has been necessary. LN*n*H, a symmetrically branched HMO, and its derivatives have been synthesized chemically (Bandara et al., 2020), enzymatically (Prudden et al., 2017), and chemoenzymatically (Xiao et al., 2016). However, strategies for the challenging preparation of LNH, the asymmetrically branched scaffold, and its extensions have been rarely reported. To the best of our knowledge, only two groups have synthesized LNH and its derivatives. Danishefsky et al. developed a glycal method for synthesizing tumor-related N3 minor (difucosyllacto-*N*-neohexose) and N3 major (difucosyllacto-*N*-hexose) antigens; the difucosyllacto-*N*-hexose was composed of an LNH structure with two fucose moieties attached to its LNB and LacNAc branches (Kim et al., 2001). Demchenko et al. reported an elegant (4 + 2) strategy for achieving the total synthesis of LNH, in which LN*n*H could also be prepared en route to the target glycan (Bandara et al., 2019b). Wang et al. developed a systematic strategy for preparing a library of branched fucosylated and sialylated HMOs with the symmetrical LN*n*H backbone by using enzymatic glycan extensions on three chemically preassembled branched core structures, but they did not address the LNH synthesis (Xiao et al., 2016). One of the critical bottlenecks in the branched HMO synthesis is the selective sugar unit elongation on the desired glycan branch upon enzymatic catalysis. Neither enzymatic nor chemoenzymatic strategies for the synthesis of LNH and its derivatives have been reported prior to this study.

We previously analyzed the versatile catalytic properties of fucosyltransferases (Fang et al., 2018; Tsai et al., 2019b; Huang et al., 2019). When a novel substrate design is employed, the inherent substrate preferences of the enzyme enable it to selectively transfer a fucose moiety onto the desired site of a glycan (Huang et al., 2021). This feature allows for the production of structurally complex multifucosylated HMOs at a preparative scale. Substrate-controlled glycosylation in enzymatic synthesis represents a solution for synthesizing asymmetrically branched HMOs. Recently, Boons et al. reported a stop-and-go strategy where the *N*-modified glucosamine moiety on the branched *N*-glycan was installed as a switch to trigger selective enzymatic β1,4-galactosylation; this enabled the synthesis of asymmetrically branched multiantennary *N*-glycans (Liu et al., 2019). Inspired by their substrate design and as part of our ongoing project to develop highly efficient approaches for HMO synthesis, we herein proposed a facile strategy for selective enzymatic glycan extension on a branched scaffold to complete the chemoenzymatic synthesis of LNH-Cbz **1b**. The hexylcarboxylbenzyl linker on the glycan can facilitate end-product purification, and the deprotected amine-functionalized product can be immobilized onto glass slides for further glycan array development. Another branched HMO, LN*n*H-Cbz **2b**, can also be obtained en route to the target molecule.

## Results and Discussion

**FIGURE 2:**
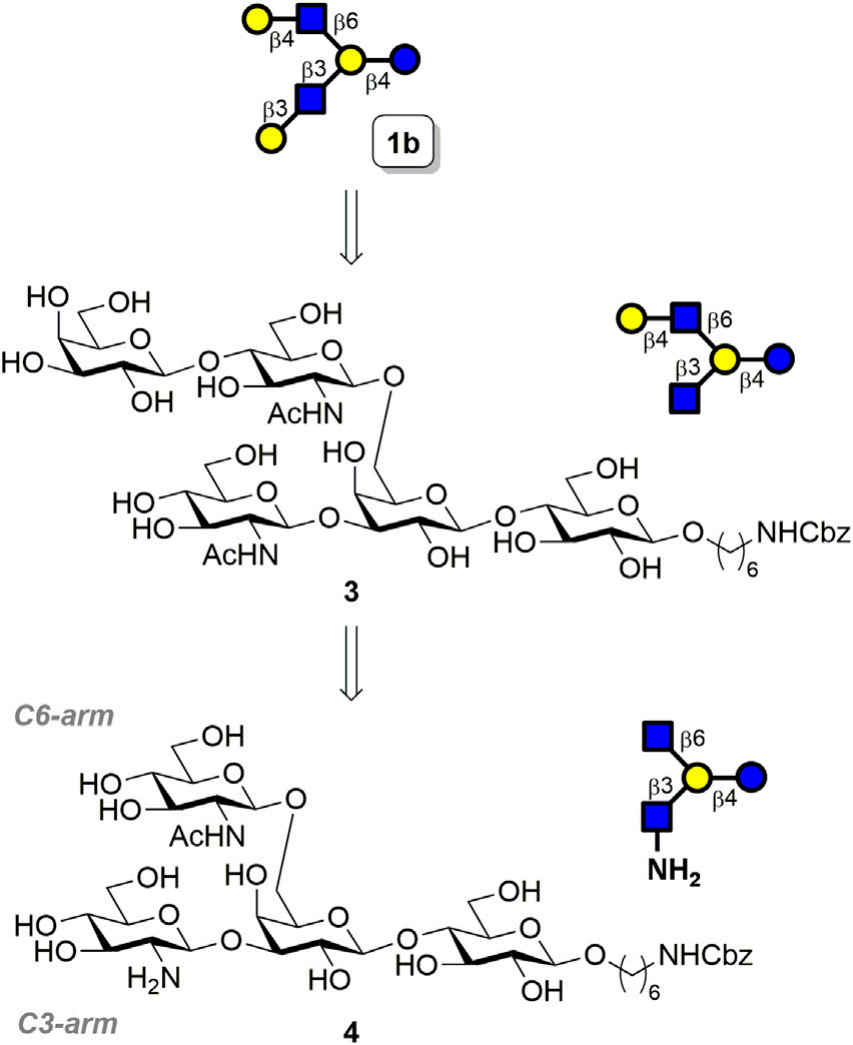
Retrosynthetic analysis of **1b**.

To design a substrate-controlled enzymatic glycosylation strategy for the selective β1,4-galactosyl extension of the GlcNAc moiety, we first examined the substrate specificity of the β1,4-galactosyltransferase enzyme from *Helicobacter pylori* 26695 (**HP0826**), which recognizes the terminal GlcNAc moiety and then converts it into type-2 LacNAc in the presence of uridine diphosphate galactose (UDP-Gal). Three β-glucosaminosides with *N*-modification were tested as substrates, including a commonly used substrate (GlcNAc-C_6_N_3_), an *N*-modified GlcNAc derivative (GlcNHBoc-C_6_N_3_), and glucosamine (GlcNH_2_-C_6_N_3_). The results indicated that **HP0826** exhibited excellent enzymatic activity on GlcNAc-C_6_N_3_ and moderate activity on GlcNHBoc-C_6_N_3_, whereas GlcNH_2_-C_6_N_3_ was a poor substrate for the enzyme (Supplementary Figure S1). Encouraged by this unique substrate specificity, we designed the branched architecture GlcNH_2_β1→3(GlcNAcβ1→6)Galβ1→4Glc **4** comprising a lactoside with a β-GlcNAc installed at C6′-OH (C6-arm) and a β-GlcNH_2_ connected at C3′-OH (C3-arm), as illustrated in Figure 2. Because **HP0826** was more active on intact GlcNAc than on GlcNH_2_, we anticipated the successful transfer of Gal onto the C6-arm, which would lead to a pentasaccharide, of which the C3-arm GlcNH_2_ would be barely recognized by the enzyme at a low UDP-Gal concentration. This feature would enable us to preferentially furnish the type-2 LacNAc at the C6-arm by exploiting the inherent substrate specificity of **HP0826**, and we could then convert GlcNH_2_ at the C3-arm into GlcNAc to obtain the LNH precursor **3** that could then be extended to type-1 LacNAc by the corresponding β1,3-galactosyltransferase to furnish the asymmetrically branched hexasaccharide LNH **1b**.

**SCHEME 1:**
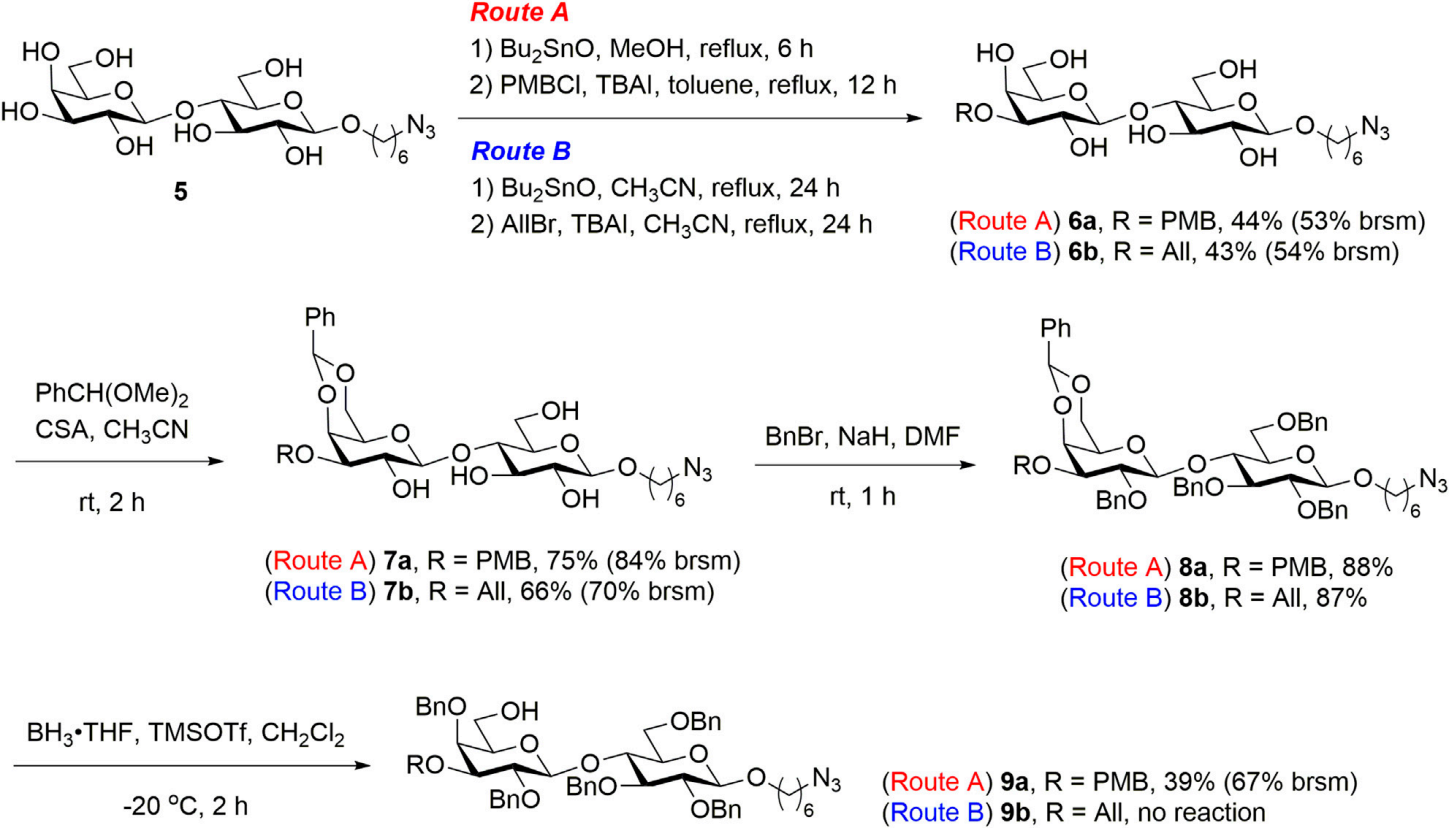
Synthesis of the disaccharide acceptor **9**.

To prepare the disaccharide **9** for chemical glycosylation at C6′-OH, the lactoside **5** was refluxed in anhydrous methanol in the presence of dibutyltin oxide, and the resulting dibutylstannylene derivatives were treated with *para*-methoxybenzyl (PMB) chloride to regioselectively introduce PMB to the C3′-OH of **5**, yielding lactoside **6a** [44% yield, 53% based on the recovered starting material (brsm)], as illustrated in Scheme 1 (Route A). The disaccharide **6a** was treated with benzaldehyde dimethyl acetal in the presence of camphorsulfonic acid in acetonitrile to yield lactoside **7a** (75% yield, 84% brsm). The remaining hydroxyl groups of **7a** were protected with benzyl groups, following a benzylation process to yield the fully protected disaccharide **8a** (88% yield). Under borane tetrahydrofuran complex (BH_3_·THF) and trimethylsilyl trifluoromethanesulfonate (TMSOTf) treatment, the regioselective reductive ring opening of the benzylidene acetal of disaccharide **8a** provided lactoside **9a** with a C6′-OH for a modest yield of 39% (67% brsm). To produce sufficient amounts of disaccharide acceptors for chemical glycosylation on C6′-OH, we developed an alternative route for introducing an allyl group at the C3′-OH of lactoside **5**, which resulted in the formation of **6b** and the corresponding derivatives **7b** and **8b** (Route B). However, all attempts to deprotect **8b** by using BH_3_·THF were unsuccessful, and no desired product was formed. Instead, we observed numerous unidentified side products derived from the starting material. Possible coordination between BH_3_ and the allyl at C3′ of **8b** and trace amounts of moisture that would lead to deprotection of benzylidene under acidic conditions might be the reason for unsuccessful regioselective ring opening of **8b**. As the preparation of the disaccharide acceptor *via* route B was not practicable, we focused on the reaction optimization of route A.

**SCHEME 2:**
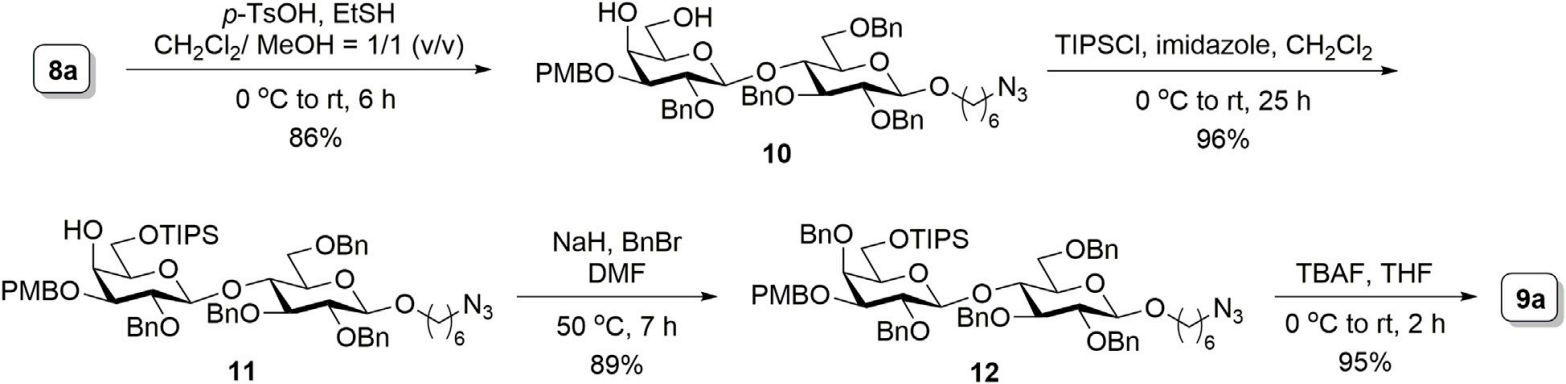
Synthesis of the disaccharide acceptor **9a**.

Because the yield from the selective ring opening experiment was not satisfactory in our case, an alternative route was considered. The conventional strategy of protecting the to-be-glycosylated hydroxyl with a silyl group was applied. Accordingly, as depicted in Scheme 2, the ring opening of **8a** was conducted using the acidic catalysis of *p*-toluenesulfonic acid and ethanethiol, an approach adapted from another study (Bandara et al., 2019b), to produce **10** (86% isolated yield). Selective silylation on the primary hydroxyl group of **10** with triisopropylsilyl chloride and imidazole resulted in **11** (96% yield). The remaining hydroxyl group was then benzylated in the presence of sodium hydride and benzyl bromide to obtain **12** (89% yield). Subsequent deprotection of the silyl group by using tetra-*n*-butylammonium fluoride produced the disaccharide acceptor **9a** (95% yield). Although this route required manipulations of an additional protecting group to transform **8a** to **9a**, the increased overall yield (70% for four steps) provided a sufficient amount of material for further synthesis.

**SCHEME 3:**
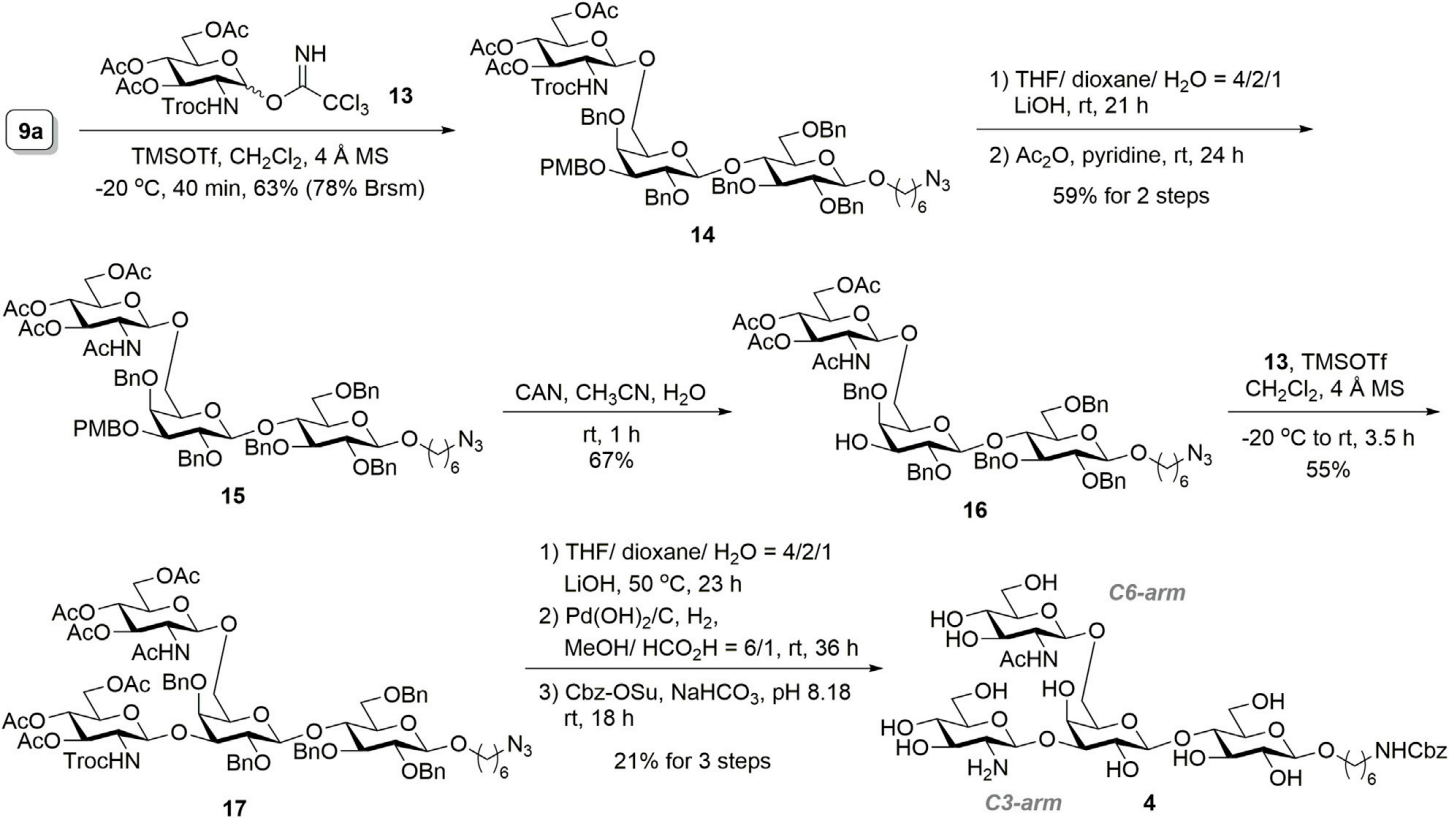
Synthesis of tetrasaccharide **4**.

The construction of tetrasaccharide **4** was achieved through sequential installations of the glucosamine residue on **9a**, as illustrated in Scheme 3. Glycosylation of the 6′-*O*-unprotected acceptor **9a** with glycosyl donor **13** (Mitachi et al., 2014) proceeded at −20°C in the presence of catalytic TMSOTf, and the desired trisaccharide **14** was obtained (63% yield, 78% brsm) with complete β-stereoselectivity. The 2,2,2-trichloroethoxycarbonyl (Troc) group of **14** was converted to an acetyl group by executing a two-step protecting group manipulation, which resulted in **15** (59% yield over two steps). Accordingly, concomitant deprotection of the Troc and ester groups of **14** was accomplished by using lithium hydroxide in a THF/dioxane/H_2_O solution (4:2:1 ratio). The crude product was then desalted using a C_18_ solid-phase extraction (SPE) cartridge, which was followed by treatment with acetic anhydride and pyridine to convert amine to acetamide and reacetylation of the remaining hydroxyls to complete the transformation. Selective removal of the PMB group of **15** through oxidation was achieved through a reaction with ceric ammonium nitrate, producing trisaccharide acceptor **16** (67% yield). TMSOTf-promoted glycosylation of **16** with glycosyl donor **13** yielded the desired tetrasaccharide **17** with complete β-selectivity (55% isolated yield). To minimize product loss in individual purification steps, we performed a three-step global deprotection process. First, the hydrolysis of acetates and the Troc-protecting group was directly conducted using lithium hydroxide. Benzyl ethers subsequently underwent hydrogenolysis by palladium hydroxide on carbon using a hydrogen balloon, which was followed by the selective conversion of the primary amine to *N*-benzyloxycarbonyl (NHCbz) on the linker in sodium bicarbonate buffer. After the desalting step by HW-40F size-exclusion chromatography, the key tetrasaccharide precursor **4** for selective enzymatic glycan extension was obtained (21% yield) in three steps. The stereochemistry of the anomeric centers was confirmed by proton nuclear magnetic resonance spectroscopy, which showed GlcNAc and GlcNH_2_ anomeric peaks as two doublet peaks at 4.64 and 4.65 ppm, with coupling constants of 8.1 and 7.2 Hz, respectively, suggesting a *β*-configuration. Furthermore, confirmation of the glycan linkages was obtained from the heteronuclear multiple bond correlation spectrum data, which revealed the β1,6-linkage of GlcNAc to Gal and β1,3-linkage of GlcNH_2_ to the Gal moiety.

**SCHEME 4:**
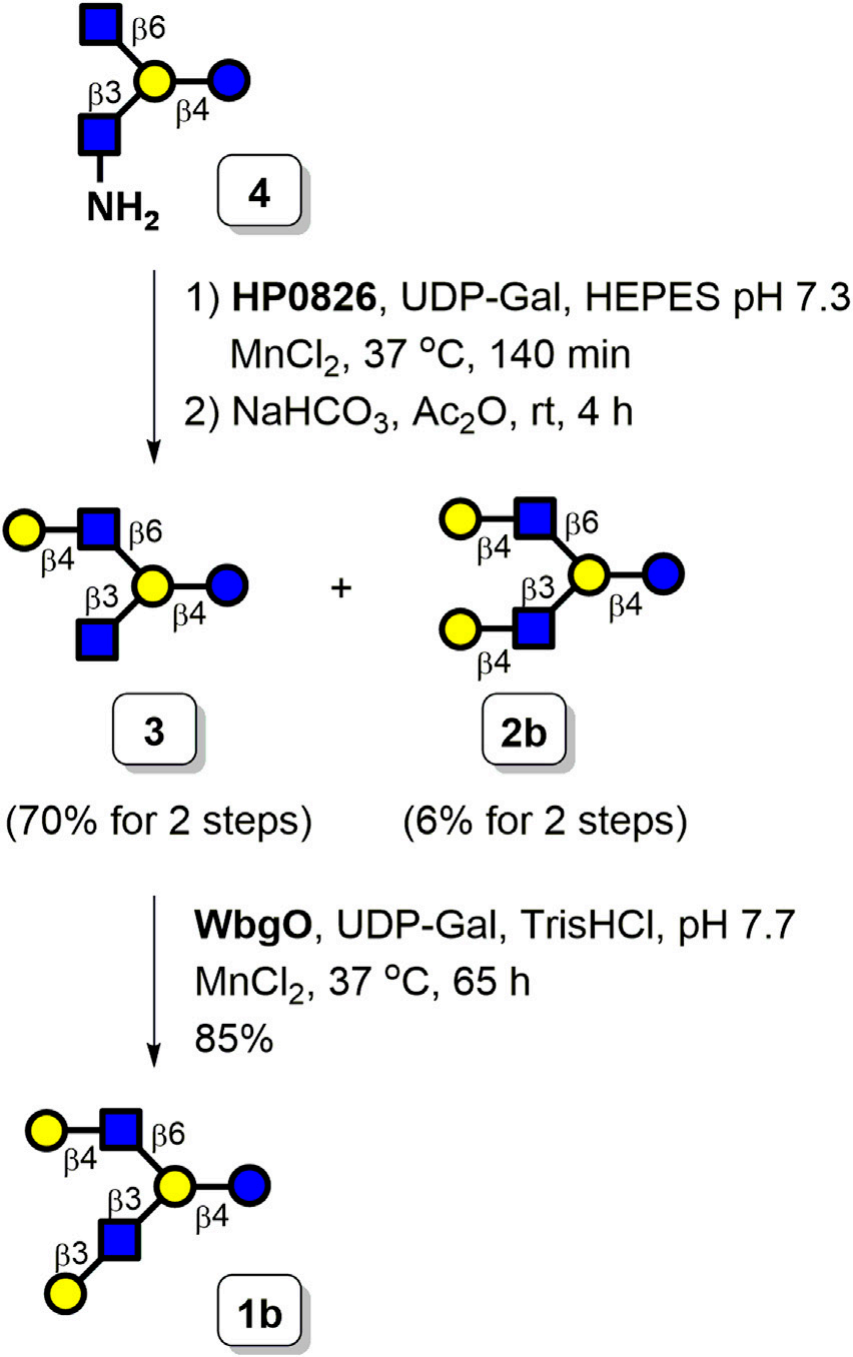
Enzymatic synthesis of LNH **1b** and LN*n*H **2b**.

Next, we prepared asymmetrically branched LNH starting from tetrasaccharide **4** and employed bacterial glycosyltransferases to selectively install galactosyl moieties. A preliminary examination of the substrate specificity indicated that the GlcNAc moiety can be galactosylated by **HP0826** faster than the GlcNH_2_ acceptor. The selectively enzymatic β1,4-galactosylation of tetrasaccharide **4** was conducted by **HP0826**. Once the C6-arm was capped with Gal to prevent further elongation of this position, GlcNH_2_ could be acetylated to yield natural GlcNAc, which was capable of being extended by β1,3-galactosyltransferase. The successfully selective β1,4-galactosylation on the C6-arm of the branched tetrasaccharide was as we predicted, thus proving our hypothesis. Tetrasaccharide **4** was incubated with **HP0826** in HEPES buffer (pH 7.3) in the presence of manganese (II) chloride and UDP-Gal (1.5 equiv), which was followed by *N*-acetylation of the GlcNH_2_ unit by acetic anhydride in the sodium bicarbonate solution. As depicted in Scheme 4, one monogalactosylated product (**3**) and one digalactosylated product (**2b**, LN*n*H-Cbz) were formed with isolation yields of 70% (4 mg) and 6% (0.5 mg), respectively. When the amount of UDP-Gal was raised to 2 equiv in the **HP0826** reaction, the isolation yield of **3** decreased to 48% (2.7 mg), whereas the isolation yield of LN*n*H-Cbz (**2b**) increased to 26% (2.2 mg). The results indicated that **HP0826** displayed its catalytic preference for the GlcNAc moiety and proceeded to initiate the reaction on a given molecule with GlcNAc and GlcNH_2_ simultaneously present. Nevertheless, digalactosylation of **4** could be achieved with a fair yield by increasing the donor concentration. Additionally, separation of the resulting products **3** and **2b** could be easily accomplished using size-exclusion chromatography with a gravity-flow system. The asymmetrically branched HMO hexasaccharide LNH (**1b**) was prepared by treating **3** with **WbgO** (β1,3-galactosyltransferase from *Escherichia coli* O55), manganese (II) chloride, and UDP-Gal in the presence of Tris buffer (pH 7.7). By combining the use of a C_18_ SPE column with gel filtration, we obtained the desired LNH-Cbz (**1b**) with a satisfactory yield of 85%.

## Materials and Methods

### General Methods

The chemicals for the synthesis were all obtained from Acros, Merck Millipore, Fluka, Tokyo Chemical Industry (TCI), or Sigma-Aldrich and used without further purification unless otherwise stated. Thin layer chromatography (TLC) was performed on Merck silica gel plates 60 F_254_. TLC plates were visualized under UV light (254 or 365 nm) and by treatment with *p*-anisaldehyde, ninhydrin, or cerium molybdate staining solution, followed by heating. Silica gel flash column chromatography was conducted on Silica gel 60 (Merck Millipore, 40–63 μm) or Silia Flash Irregular Silica Gel P60 (SiliCycle, 40–63 μm). Reversed‐phase silica gel chromatography was performed using Macherey-Nagel Chromabond^®^ C18 ec (70 ml/10 g). Size-exclusion chromatography gel HW-40F was purchased from Tosoh Bioscience (Tokyo, Japan). Cartridges were preconditioned by washing with five bed volumes of methanol, followed by five bed volumes of 50% methanol in deionized water and then five bed volumes of deionized water, followed by water-methanol gradient elution. The protein concentrations were determined with the Bradford protein assay (Bio-Rad) and Pierce™ BCA protein assay kit (Thermo Fisher Scientific) using bovine serum albumin as the standard. Protein purification devices were using centrifugal filter devices Vivaspin^®^ Turbo 15 10 kDa MWCO (Sartorius) or Amicon^®^ Ultra 4 ml Centrifugal Filters 10 kDa MWCO (Merck Millipore). ^1^H and ^13^C nuclear magnetic resonance spectra were recorded on the Bruker DPX 400 MHz NMR spectrometer and the Varian Unity INOVA 500 MHz NMR spectrometer at the National Chung Cheng University NMR facility and Varian VNMRS 700 MHz at National Tsing Hua University. All spectra were recorded using an internal lock (deuterium) and are referenced to a residual solvent peak. ^1^H and ^13^C NMR spectra were referenced to the solvent used (D_2_O, *δ* 4.79 ppm for ^1^H; CDCl_3_, *δ* 7.24, and 77 ppm; CD_3_OD, *δ* 3.31, and 49 ppm at 298 K for ^1^H and ^13^C, respectively). ^1^H and ^13^C chemical shifts are quoted in parts per million (ppm) downfield of tetramethylsilane, and chemical shifts (δ) are rounded to the nearest 0.1 ppm unless increased precision was required to distinguish resonances, and coupling constants (*J*) were reported in hertz (Hz) as units. The following abbreviations were used to indicate multiplicities: s = singlet, d = doublet, t = triplet, q = quartet, qui. = quintet, dd = doublet of doublets, ddd = doublet of doublet of doublets, and m = multiplet. High-resolution electrospray ionization (ESI) mass spectra were recorded by Waters ESI-Q-TOF at the Mass Laboratory in the Institute of Chemistry, Academia Sinica. HP0826 (β1,4-galactosyltransferase enzyme from *H. pylori* 26695) and WbgO (β1,3-galactosyltransferase from *E. coli* O55) were recombinantly expressed according to the reported literature (Fang et al., 2018).

### 6-Carboxybenzyl-Aminohexyl *β*-D-Galactopyranosyl-(1→3)-2-Acetamido-2-Deoxy-*β*-D-Glucopyranosyl-(1→3)-[*β*-D-Galactopyranosyl-(1→4)-2-Acetamido-2-Deoxy-*β*-D-Glucopyranosyl-(1→6)]-*β*-D-Galactopyranosyl-(1→4)-*β*-D-Glucopyranoside (1b)

A mixture of **3** (3.4 mg, 2.97 μmol, 20 mM), UDP-Gal (30 mM), manganese (II) chloride (30 mM), WbgO (0.65 mg/ml), and alkaline phosphatase (1 μl/ml) in a buffered (30 mM Tris-HCl, pH 7.7) solution (150 μl) was incubated at 37°C with agitation at 200 rpm for 65 h. The product formation was monitored by TLC. When the TLC analysis indicated the completion of the reaction, the reaction solution was quenched by heating at 100°C for 10 min. The resulting mixture was centrifuged (4°C, 10,000 × *g*, 10 min) to remove the proteins and insoluble precipitates. The supernatant was then concentrated, purified by Chromabond^®^ C18 ec column, followed by passing through a HW-40F size-exclusion chromatography (1.5 cm × 75 cm) with water to afford compound **1b** (3.3 mg, 85%) as the white powder. R_f_ = 0.57 [*n*-propanol/water/acetic acid = 6/2/1 (v/v/v), 2 runs]; ^1^H NMR (700 MHz, D_2_O) *δ* 7.48–7.38 (m, 5H, 5×CH _Ph_), 5.11 (s, 2H, CH_2 Cbz_), 4.73 (d, *J* = 8.5 Hz, 1H, H-1 _GlcNAc-2_), 4.64 (d, *J* = 7.8 Hz, 1H, H-1 _GlcNAc-1_), 4.49–4.41 (m, 4H, H-1 _Gal-2_, H-1 _Glc_, H-1 _Gal-3_, H-1 _Gal-1_), 4.15 (d, *J* = 2.9 Hz, 1H, H-4 _Gal-1_), 4.02–3.97 (m, 2H, H-6b _Gal-1_, H-6b _GlcNAc-1_), 3.95 (d, *J* = 11.6 Hz, 1H, H-6b _Glc_), 3.94–3,87 (m, 5H, H-4 _Gal-2_, H-4 _Gal-3_, OC*H*H, H-6b _GlcNAc-2_, H-2 _GlcNAc-2_), 3.87–3.81 (m, 4H, H-6a _GlcNAc-1_, H-6a _Gal-1_, H-5 _Gal-1_, H-3 _GlcNAc-2_), 3.81–3.69 (m, 12H, H-6 _Gal-3_, H-6 _Gal-2_, H-6a _GlcNAc-2_, H-6a _Glc_, H-2 _GlcNAc-1_, H-4 _GlcNAc-1_, H-3 _Gal-1_, H-3 _GlcNAc-1_, H-5 _Gal-3_, H-5 _Gal-2_), 3.68–3.57 (m, 9H, H-3 _Gal-2_, OCH*H*, H-3 _Gal-3_, H-3 _Glc_, H-5 _GlcNAc-1_, H-2 _Gal-1_, H-4 _Glc_, H-2 _Gal-2_, H-2 _Gal-3_), 3.57–3.47 (m, 3H, H-2 _Gal-2_, H-2 _Gal-3_, H-5 _GlcNAc-2_), 3.31 (t, *J* = 8.6 Hz, 1H, H-2 _Glc_), 3.12 (t, *J* = 6.3 Hz, 2H, CH_2_NHCbz), 2.06 (s, 3H, CH_3 Ac_), 2.03 (s, 3H, CH_3 Ac_), 1.65–1.57 (m, 2H, CH_2_), 1.53–1.46 (m, 2H, CH_2_), 1.40–1.28 (m, 4H, 2 × CH_2_); ^13^C NMR (175 MHz, D_2_O) *δ* 176.62 (C _Ac_), 176.18 (C _Ac_), 160.15 (C _Cbz_), 138.35 (C _Cbz_), 130.44 (CH _Ph_), 129.96 (CH _Ph_), 129.20 (CH _Ph_), 105.14 (C-1 _Gal-3_), 104.66 (C-1 _Gal-1_), 104.54 (C-1 _Gal-2_), 104.18 (C-1 _GlcNAc-2_), 103.64 (C-1 _Glc_), 102.63 (C-1 _GlcNAc-1_), 83.69 (C-3 _GlcNAc-2_), 83.37 (C-3 _Gal-1_), 80.62 (C-4 _Glc_), 80.02 (C-4 _GlcNAc-1_), 77.00 (C-5 _Gal-2_), 76.93 (C-5 _Gal-3_), 76.84 (C-5 _GlcNAc-2_), 76.41 (C-5 _GlcNAc-1_), 76.31 (C-5 _Glc_), 76.13 (C-3 _Glc_), 75.12 (C-5 _Gal-1_), 74.52 (C-2 _Glc_), 74.15 (C-3 _Gal-2_), 74.12 (C-3 _GlcNAc-1_, C-3 _Gal-3_), 72.61 (C-2 _Gal-2_), 72.33 (C-2 _Gal-3_), 72.20 (OCH_2_), 71.52 (C-2 _Gal-1_), 70.35 (C-6 _Gal-1_), 70.18 (C-4 _Gal-2_, C-4 _GlcNAc-2_), 70.09 (C-4 _Gal-3_), 70.03 (C-4 _Gal-1_), 68.35 (CH_2 Cbz_), 62.68 (C-6 _Gal-2_, C-6 _Gal-3_), 62.15 (C-6 _GlcNAc-2_), 61.67 (C-6 _GlcNAc-1_, C-6 _Glc_), 56.67 (C-2 _GlcNAc-1_), 56.36 (C-2 _GlcNAc-2_), 41.95 (CH_2_NHCbz), 30.24 (2 × CH_2_), 27.12 (CH_2_), 26.26 (CH_2_), 24.07 (CH_3 Ac_), 23.87 (CH_3 Ac_); HRMS (ESI) *m/z* calcd for C_54_H_87_N_3_O_33_Na [M + Na]^+^: 1328.5114; found 1328.5118.

### 6-Carboxybenzyl-Aminohexyl *β*-D-Galactopyranosyl-(1→4)-2-Amino-2-Deoxy-*β*-D-Glucopyranosyl-(1→3)-[*β*-D-Galactopyranosyl-(1→4)-2-Acetamido-2-Deoxy-*β*-D-Glucoyranosyl-(1→6)]-*β*-D-Galactopyranosyl-(1→4)-*β*-D-Glucopyranoside (2b)

Compound **2b** R_f_ = 0.50 [propanol/water/acetic acid = 6/2/1 (v/v/v), 2 runs]; ^1^H NMR (500 MHz, D_2_O) *δ* 7.52–7.39 (m, 5H, 5 × CH _Ph_), 5.13 (s, 2H, CH_2 Cbz_), 4.73 (d, *J* = 8.3, 1H, H-1 _GlcNAc-2_), 4.66 (d, *J* = 7.5 Hz, 1H, H-1 _GlcNAc-1_), 4.53–4.46 (m, 3H, H-1 _Gal-2_, H-1 _Glc_, H-1 _Gal-3_), 4.45 (d, *J* = 7.9 Hz, 1H, H-1 _Gal-1_), 4.17 (d, *J* = 3.0 Hz, 1H, H-4 _Gal-1_), 4.05–3.93 (m, 6H, H-6b _Gal-1_, H-6b _GlcNAc-1_, H-6b _GlcNAc-2_, H-6b _Glc_, H-4 _Gal-2_, H-4 _Gal-3_), 3.93–3.81 (m, 7H, H-6a _GlcNAc-1_, H-6a _Gal-1_, H-6a _GlcNAc-2_, H-6a _Glc_, OC*H*H, H-2 _GlcNAc-2_, H-5 _Gal-1_), 3.81–3,71 (m, 12H, H-3 _GlcNAc-2_, H-3 _GlcNAc-1_, H-5 _Gal-3_, H-5 _Gal-2_, H-4 _GlcNAc-1_, H-4 _GlcNAc-2_, H-3 _Gal-1_, H-6 _Gal-3_, H-6 _Gal-2_, H-2 _GlcNAc-1_), 3.71–3.66 (m, 3H, H-3 _Gal-2_, H-3 _Gal-3_, OCH*H*), 3.66–3.53 (m, 8H, H-2 _Gal-1_, H-2 _Gal-2_, H-2 _Gal-3_, H-5 _Glc_, H-5 _GlcNAc-1_, H-5 _GlcNAc-2_, H-3 _Glc_, H-4 _Glc_), 3.32 (t, *J* = 8.5 Hz, 1H, H-2 _Glc_), 3.14 (t, *J* = 6.3 Hz, 2H, C*H*_2_NHCbz), 2.08 (s, 3H, CH_3 Ac_), 2.06 (s, 3H, CH_3 Ac_), 1.67–1.59 (m, 2H, CH_2_), 1.55–1.47 (m, 2H, CH_2_), 1.42–1.28 (m, 4H, 2 × CH_2_); ^13^C NMR (125 MHz, D_2_O) *δ* 176.56 (C _Ac_), 176.18 (C _Ac_), 160.21 (C _Cbz_), 138.21 (C _Cbz_), 130.44 (CH _Ph_), 129.97 (CH _Ph_), 129.22 (CH _Ph_), 104.67 (C-1 _Gal-1_), 104.53 (C-1 _Gal-2_, C-1 _Gal-3_), 104.36 (C-1 _GlcNAc-2_), 103.65 (C-1 _Glc_), 102.63 (C-1 _GlcNAc-1_), 83.39 (C-3 _Gal-1_), 80.62 (C-4 _Glc_), 80.06 (C-4 _GlcNAc-1_), 79.82 (C-4 _GlcNAc-2_), 77.01 (C-5 _Gal-2_, C-5 _Gal-3_), 76.41 (C-5 _GlcNAc-1_), 76.31 (C-5 _Glc_), 76.22 (C-5 _GlcNAc-2_), 76.13 (C-3 _Glc_), 75.10 (C-5 _Gal-1_), 74.54 (C-2 _Glc_), 74.16 (C-3 _Gal-2_, C-3 _Gal-3_), 74.10 (C-3 _GlcNAc-1_), 73.84 (C-3 _GlcNAc-2_), 72.61 (C-2 _Gal-2_, C-2 _Gal-3_), 72.20 (OCH_2_), 71.48 (C-2 _Gal-1_), 70.30 (C-6 _Gal-1_), 70.21 (C-4 _Gal-2_, C-4 _Gal-3_), 70.04 (C-4 _Gal-1_), 68.36 (CH_2 Cbz_), 62.67 (C-6 _Gal-2_, C-6 _Gal-3_), 61.70 (C-6 _GlcNAc-1_, C-6 _Glc_), 61.56 (C-6 _GlcNAc-2_), 56.67 (C-2 _GlcNAc-2_), 56.67 (C-2 _GlcNAc-1_), 41.95 (CH_2_NHCbz), 30.24 (2×CH_2_), 27.11 (CH_2_), 26.26 (CH_2_), 24.08 (CH_3 Ac_), 23.83 (CH_3 Ac_); HRMS (ESI) *m/z* calcd for C_54_H_87_N_3_O_33_Na [M + Na]^+^: 1328.5114; found 1328.5111.

### 6-Carboxybenzyl-Aminohexyl 2-Amino-2-Deoxy-*β*-D-Glucopyranosyl-(1→3)-[*β*-D-Galactopyranosyl-(1→4)-2-Acetamido-2-Deoxy-*β*-D-Glucopyranosyl-(1→6)]-*β*-D-Galactopyranosyl-(1→4)-*β*-D-Glucopyranoside (3)

A mixture of **4** (167 μl, 30 mM), UDP-Gal (60 mM), manganese (II) chloride (10 mM), HP0826 cell lysate (20%, v/v), and alkaline phosphatase (1 μl/ml) in a buffered (1 M HEPES, pH 7.3) solution (25 μl) was incubated at 37°C with agitation at 200 rpm for 140 min. The product formation was monitored by TLC [ethyl acetate/methanol/water/acetic acid = 10:4:2:1 (v/v/v/v), R_f_ = 0.46]. When the TLC analysis indicated the completion of the reaction, the reaction solution was quenched by heating at 100°C for 10 min. The resulting mixture was centrifuged (4°C, 10,000 × *g*, 10 min) to remove the proteins and insoluble precipitates. The supernatant was concentrated and purified by using the Chromabond^®^ C18 ec column [50 mM ammonium bicarbonate/methanol = 10/3.5 (v/v)], followed by passing through HW-40F gel chromatography (1.5 cm × 75 cm) with 50 mM of ammonium bicarbonate to obtain the desired product. Product-containing fractions were identified by the TLC analysis, were pooled together, and then lyophilized to give the white powder. Sodium bicarbonate (0.5 mg, 6 μmol, 1.2 eq.) and acetic anhydride (5 μl, 50 μmol, 10 eq.) were added to a solution of the resulting compound (4.7 mg, 5 μmol, 1.0 eq.) in water (0.5 ml). After being stirred for 4 h, the solution was concentrated in *vacuo*. The resulting mixture was purified by the Chromabond^®^ C18 ec column [50 mM ammonium bicarbonate/methanol = 1/1 (v/v)], followed by passing through HW-40F gel chromatography (1.5 cm × 75 cm) with 50 mM ammonium bicarbonate to afford compound **3** (4 mg, 70% for two steps) and compound **2b** (0.5 mg, 6% for two steps) as the white powder. Compound **3** R_f_ = 0.63 [propanol/water/acetic acid = 6/2/1 (v/v/v/v), 2 runs]; ^1^H NMR (500 MHz, D_2_O) *δ* 7.50–7.39 (m, 5H, 5 × CH _Ph_), 5.13 (s, 2H, CH_2 Cbz_), 4.71 (d, *J* = 8.4 Hz, 1H, H-1 _GlcNAc-2_), 4.66 (d, *J* = 7.7 Hz, 1H, H-1 _GlcNAc-1_), 4.49 (d, *J* = 7.8 Hz, 1H, H-1 _Gal-2_), 4.48 (d, *J* = 8.0 Hz, 1H, H-1 _Glc_), 4.45 (d, *J* = 7.9 Hz, 1H, H-1 _Gal-1_), 4.16 (d, *J* = 3.3 Hz, 1H, H-4 _Gal-1_), 4.05–3.90 (m, 6H, H-6b _Gal-1_, H-6b _GlcNAc-1_, H-6b _Glc_, H-4 _Gal-2_, H-6b _GlcNAc-2_, OC*H*H), 3.90–3.81 (m, 4H, H-6a _GlcNAc-1_, H-6a _Gal-1_, H-5 _Gal-1_, H-6a _Glc_), 3.81–3.71 (m, 9H, H-6a _GlcNAc-2_, H-2 _GlcNAc-2_, H-6 _Gal-2_, H-2 _GlcNAc-1_, H-3 _GlcNAc-1_, H-5 _Gal-2_, H-4 _GlcNAc-1_, H-3 _Gal-1_), 3.71–3.53 (m, 9H, H-3 _Gal-2_, OCH*H*, H-3 _Glc_, H-5 _Glc_, H-5 _GlcNAc-1_, H-2 _Gal-1_, H-4 _Glc_, H-3 _GlcNAc-2_, H-2 _Gal-2_), 3.52–3.43 (m, 2H, H-4 _GlcNAc-2_, H-5 _GlcNAc-2_), 3.32 (t, *J* = 8.5 Hz, 1H, H-2 _Glc_), 3.14 (t, *J* = 6.5 Hz, 2H, C*H*_2_NHCbz), 2.07 (s, 3H, CH_3_), 2.06 (s, 3H, CH_3_), 1.68–1.58 (m, 2H, CH_2_), 1.55–1.47 (m, 2H, CH_2_), 1.43–1.29 (m, 4H, 2 × CH_2_); ^13^C NMR (125 MHz, D_2_O) *δ* 176.60 (C _Ac_), 176.18 (C _Ac_), 160.19 (C _Cbz_), 138.38 (C _Cbz_), 130.44 (CH _Ph_), 129.96 (CH _Ph_), 129.21 (CH _Ph_), 104.66 (C-1 _Gal-1_), 104.54 (C-1 _Gal-2_), 104.46 (C-1 _GlcNAc-2_), 103.65 (C-1 _Glc_), 102.63 (C-1 _GlcNAc-1_), 83.35 (C-3 _Gal-1_), 80.62 (C-4 _Glc_), 80.05 (C-4 _GlcNAc-1_), 77.32 (C-5 _GlcNAc-2_), 77.00 (C-5 _Gal-2_), 76.41 (C-5 _GlcNAc-1_), 76.31 (C-5 _Glc_), 76.14 (C-3 _Glc_), 75.22 (C-3 _GlcNAc-2_), 75.12 (C-5 _Gal-1_), 74.53 (C-2 _Glc_), 74.16 (C-3 _Gal-2_), 74.10 (C-3 _GlcNAc-1_), 72.61 (C-2 _Gal-2_), 72.20 (OCH_2_), 71.52 (C-2 _Gal-1_), 71.33 (C-4 _GlcNAc-2_), 70.20 (C-4 _Gal-2_), 70.05 (C-4 _Gal-1_), 68.34 (CH_2__Cbz_), 62.67 (C-6 _Gal-2_), 62.14 (C-6 _GlcNAc-2_), 61.68 (C-6 _GlcNAc-1_, C-6 _Glc_), 57.33 (C-2 _GlcNAc-2_), 56.67 (C-2 _GlcNAc-1_), 41.95 (CH_2_NHCbz), 30.24 (2 × CH_2_), 27.11 (CH_2_), 26.26 (2 × CH_2_), 24.08 (CH_3 Ac_), 23.81 (CH_3 Ac_); HRMS (ESI) *m/z* calcd for C_48_H_77_N_3_O_28_Na [M + Na]^+^: 1166.4585; found 1166.4563.

### 6-Carboxybenzyl-Aminohexyl 2-Deoxy-2-Amino-*β*-D-Glucopyranosyl-(1→3)-[2-Acetamido-2-Deoxy-*β*-D-Glucopyranosyl-(1→6)]-*β*-D-Galactopyranosyl-(1→4)-*β*-D-Glucopyranoside (4)

Compound **17** (114 mg, 0.067 mmol) was dissolved in a mixture of tetrahydrofuran/dioxane/water = 4/2/1 (2.2 ml), and then lithium hydroxide (28 mg, 1.17 mmol, 17.5 eq.) was added. The mixture was stirred at room temperature for 23 h. The solution was neutralized by adding 1N HCl and then concentrated *in vacuo*. The residue was purified by Chromabond^®^ C18 ec column (methanol) to afford crude compound. Then, a solution of the resulting crude product and palladium hydroxide (20 wt% on carbon, 351 mg, 0.2 mmol, 3.0 eq.) in methanol (2.87 ml) and formic acid (0.48 ml) was stirred under hydrogen using a balloon. After being stirred for 36 h, the solution was filtered through celite pad and concentrated *in vacuo* to afford a crude product. Finally, a solution of the crude compound in sodium bicarbonate buffer, pH 8.2 (2.2 ml) was added *N*-(benzyloxycarbonyloxy)succinimide (Cbz-OSu, 19 mg, 77 μmol, 1.3 eq.). After being stirred for 18 h, the solution was concentrated *in vacuo*, then purified by using Chromabond^®^ C18 ec column (50 mM ammonium bicarbonate/methanol = 1/1) to afford compound **4** as the white powder in 21% for three steps (13 mg). R_f_ = 0.37 [ethyl acetate/methanol/water/acetic acid = 10/4/2/1 (v/v/v/v)]; ^1^H NMR (500 MHz, D_2_O) *δ* 7.49–7.38 (m, 5H, 5 × CH _Ph_), 5.12 (s, 2H, CH_2 Cbz_), 4.65 (d, *J* = 7.2 Hz, 1H, H-1 _GlcN_), 4.64 (d, *J* = 8.1 Hz, 2H, H-1 _GlcNAc_), 4.50 (d, *J* = 7.9 Hz, H-1 _Gal_), 4.47 (d, *J* = 8.0 Hz, H-1 _Glc_), 4.18 (d, *J* = 3.2 Hz, 1H, H-4 _Gal_), 4.05–3.84 (m, 7H, H-5 _Gal_, H-6 _Gal_, OC*H*H, H-6b _Glc_, H-6b _GlcNAc_, H-6b _GlcN_), 3.84–3.75 (m, 4H, H-3 _Gal_, H-6a _Glc_, H-6a _GlcNAc_, H-6a _GlcN_), 3.75–3.61 (m, 5H, H-2 _GlcNAc_, H-2 _Gal_, OCH*H*, H-3 _Glc_, H-4 _Glc_), 3.61–3.55 (m, 2H, H-3 _GlcNAc_, H-5 _Glc_), 3.52–3.40 (m, 5H, H-3 _GlcN_, H-4 _GlcN_, H-5 _GlcN_, H-4 _GlcNAc_, H-5 _GlcNAc_), 3.33 (t, *J* = 8.5 Hz, 1H, H-2 _Glc_), 3.13 (t, *J* = 6.5 Hz, 2H, CH_2_NHCbz), 2.76 (td, *J* = 7.9, 2.6 Hz, 1H, H-2 _GlcN_), 2.08 (s, 3H, CH_3 NHAc_), 1.68–1.59 (m, 2H, CH_2_), 1.55–1.46 (m, 2H, CH_2_), 1.342–1.29 (m, 4H, 2×CH_2_); ^13^C NMR (125 MHz, D_2_O) *δ* 176.20 (C _NHAc_), 160.08 (C _Cbz_), 138.30 (C _Cbz_), 130.42 (CH _Ph_), 129.95 (CH _Ph_), 129.21 (CH _Ph_), 105.84 (C-1 _GlcN_), 104.42 (C-1 _Gal_), 103.66 (C-1 _Glc_), 102.70 (C-1 _GlcNAc_), 83.42 (C-3 _Gal_), 80.65 (C-4 _Glc_), 77.57 (C-5 _GlcNAc_), 77.50 (C-5 _GlcN_), 77.00 (C-3 _GlcN_), 76.27 (C-5 _Glc_), 76.13 (C-3 _Glc_), 75.48 (C-3 _GlcNAc_), 75.15 (C-5 _Gal_), 74.52 (C-2 _Glc_), 72.17 (OCH_2_), 71.66 (C-2 _Gal_), 71.50 (C-4 _GlcNAc_), 71.17 (C-5 _GlcN_), 70.19 (C-6 _Gal_), 69.96 (C-4 _Gal_), 68.32 (CH_2__Cbz_), 62.32 (C-6 _GlcNAc_), 62.19 (C-6 _GlcN_), 61.71 (C-6 _Glc_), 58.36 (C-2 _GlcN_), 57.14 (C-2 _GlcNAc_), 41.95 (CH_2_NHCbz), 30.29 (CH_2_), 30.24 (CH_2_), 27.12 (CH_2_), 26.25 (CH_2_), 24.04 (CH_3 NHAc_); HRMS (ESI) m/z calcd for C_40_H_65_N_3_O_22_Na [M + Na]^+^: 962.3957; found 962.3956.

### 6-Azidohexyl 3-*O*-(-4-Methoxylbenzyl)-*β*-D-Galactopyranosyl-(1→4)-*β*-D-Glucopyranoside (6a)

A mixture of 6-azidohexyl *O*-*β*-D-lactose (**5**) (Fang et al., 2018) (1.45 g, 3.10 mmol, 1.0 eq.), dibutyltin oxide (926 mg, 3.72 mmol, 1.2 eq.), and anhydrous methanol (31.0 ml) was heated at reflux for 6 h. The solution was concentrated in *vacuo*. Tetrabutylammonium iodide (573 mg, 1.55 mmol, 0.5 eq.) and *p-*methoxybenzyl chloride (0.50 ml, 3.72 mmol, 1.2 eq.) were added to a solution of the resulting residue in anhydrous toluene (31.0 ml). The reaction was heated at reflux for 12 h. The solution was filtered through celite pad and concentrated in *vacuo*. The residue was chromatographed on silica gel (methanol/dichloromethane = 1/15) and purified by using Chromabond^®^ C18 ec column [methanol/water = 1/1 (v/v)] to afford compound **6a** (798 mg, 44%; brsm 53%) as a white solid. R_f_ = 0.63 [ethyl acetate/methanol/water = 10/2/1 (v/v/v)]; ^1^H NMR (500 MHz, D_2_O): *δ* 7.44 (d, *J* = 8.7 Hz, 2H, 2 × CH _PMB_), 7.05 (d, *J* = 8.6 Hz, 2H, 2 × CH _PMB_), 4.71 (d, *J* = 11.4 Hz, 1H, C*H*H _PMB_), 4.61 (d, *J* = 11.4 Hz, 1H, CH*H*_PMB_), 4.49 (d, *J* = 7.9 Hz, 1H, H-1 _Glc_), 4.46 (d, *J* = 7.7 Hz, 1H, H-1 _Gal_), 4.09 (d, *J* = 3.1 Hz, 1H, H-4 _Gal_), 4.02–3.90 (m, 2H, H-6a _Glc_, OC*H*H), 3.87 (s, 3H, CH_3 PMB_), 3.84–3.78 (m, 2H, H-6b _Glc_, H-6a _Gal_), 3.77–3.53 (m, 8H, OCH*H*, H-6b _Gal_, H-4 _Glc_, H-5 _Gal_, H-3 _Glc_, H-2 _Gal_, H-5 _Glc_, H-3 _Gal_), 3.36–3.29 (m, 3H, H-2 _Glc_, CH_2_N_3_), 1.71–1.57 (m, 4H, 2 × CH_2_), 1.46–1.36 (m, 4H, 2 × CH_2_); ^13^C NMR (125 MHz, D_2_O) *δ* 160.50 (C _PMB_), 132.04 (2 × CH _PMB_), 131.51 (C _PMB_), 115.76 (2 × CH _PMB_), 104.52 (C-1 _Gal_), 103.70 (C-1 _Glc_), 81.22 (C-3 _Gal_), 79.98 (C-4 _Glc_), 76.91 (C-5 _Gal_), 76.39 (C-5 _Glc_), 76.11 (C-3 _Glc_), 74.51 (C-2 _Glc_), 72.53 (CH_2 PMB_), 72.19 (OCH_2_), 71.72 (C-2 _Gal_), 66.90 (C-4 _Gal_), 62.70 (C-6 _Gal_), 61.76 (C-6 _Glc_), 57.04 (CH_3 PMB_), 52.79 (CH_2_N_3_), 30.25 (CH_2_), 29.53 (CH_2_), 27.31 (CH_2_), 26.26 (CH_2_); HRMS (ESI): *m/z* of C_26_H_41_N_3_O_12_Na [M + Na]^+^: calcd 610.2588; found 610.2581.

### 6-Azidohexyl 3-*O*-Allyl-*β*-D-Galactopyranosyl-(1→4)-*β*-D-Glucopyranoside (6b)

A mixture of 6-azidohexyl *O*-*β*-D-lactose (**5**) (Fang et al., 2018) (500 mg, 1.07 mmol, 1.0 eq.), dibutyltin oxide (319 mg, 1.28 mmol, 1.2 eq.), and anhydrous acetonitrile (10.7 ml) was heated at reflux for 24 h under nitrogen. Allyl bromide (278 μl, 3.21 mmol, 3.0 eq.) and tetrabutylammonium iodide (199 mg, 0.54 mmol, 0.5 eq.) was added and the reaction was heated at reflux for 24 h. The solution was concentrated in *vacuo*, chromatographed on silica gel [methanol/ethyl acetate = 1/6 (v/v)], and then purified by Chromabond^®^ C18 ec column [methanol/water = 9/11 (v/v)] to afford compound **6b** (233 mg, 43%; brsm 54%) as a white solid. R_f_ = 0.5 [methanol/ethyl acetate = 1/6 (v/v)]; ^1^H NMR (500 MHz, D_2_O): *δ* 6.07–5.95 (m, 1H, CH _Allyl_), 5.40 (d, *J* = 17.3 Hz, 1H, C*H*H _Allyl_), 5.31 (d, *J* = 10.5 Hz, 1H, CH*H*_Allyl_), 4.49 (d, *J* = 7.9 Hz, 1H, H-1 _Glc_), 4.48 (d, *J* = 7.6 Hz, 1H, H-1 _Gal_), 4.25 (dd, *J* = 12.4, *J* = 5.8 Hz, 1H, C*H*H _Allyl_), 4.17 (d, *J* = 3.0 Hz, 1H, H-4 _Gal_), 4.13 (dd, *J* = 12.4 Hz, *J* = 6.2 Hz, 1H, CH*H*_Allyl_), 4.02–3.89 (m, 2H, H-6a _Glc_, OC*H*H), 3.86–3.52 (m, 10H, H-6b _Glc_, H-6 _Gal_, OCH*H*, H-5 _Gal_, H-4 _Glc_, H-3 _Glc_, H-2 _Gal_, H-5 _Glc_, H-3 _Gal_), 3.35 (t, *J* = 7.0 Hz, 2H, CH_2_N_3_), 3.33 (t, *J* = 8.5 Hz, 1H, H-2 _Glc_), 1.71–1.60 (m, 4H, 2×CH_2_), 1.47–1.38 (m, 4H, 2×CH_2_); ^13^C NMR (125 MHz, D_2_O): *δ* 135.6 (CH _Allyl_), 120.4 (CH_2 Allyl_), 104.5 (C-1 _Gal_), 103.7 (C-1 _Glc_), 80.2 (C-3 _Gal_), 79.9 (C-4 _Glc_), 76.9 (C-5 _Gal_), 76.4 (C-5 _Glc_), 76.1(C-3 _Glc_), 74.5 (C-2 _Glc_), 72.2 (OCH_2_), 71.9 (CH_2 Allyl_), 71.6 (C-2 _Gal_), 66.7 (C-4 _Gal_), 62.7 (C-6 _Gal_), 61.8 (C-6 _Glc_), 52.8 (CH_2_N_3_), 30.2 (CH_2_), 29.5 (CH_2_), 27.3 (CH_2_), 26.3 (CH_2_); HRMS (ESI): *m/z* of C_21_H_37_N_3_O_11_Na [M + Na]^+^: calcd 530.2326; found 530.2319.

### 6-Azidohexyl 3-*O*-(-4-Methoxylbenzyl)-4,6-*O*-Benzylidine-*β*-D-Galactopyranosyl-(1→4)-*β*-D-Glucopyranoside (7a)

Benzaldehyde dimethyl acetal (126 μl, 0.84 mmol, 2.4 eq.) was added to a solution of compound **6a** (207 mg, 0.35 mmol, 1 eq.) in anhydrous acetonitrile (4.5 ml). Camphorsulfonic acid (16 mg, 0.07 mmol, 0.2 eq.) was then added to the prechilled reaction mixture at 0°C. The solution was stirred 10 min then was allowed to warm to room temperature. The reaction mixture was stirred for 2 h at room temperature and then quenched with triethylamine under an ice bath. The residue was chromatographed on silica gel [ethyl acetate/methanol = 100/1 (v/v)] to afford compound **7a** (179 mg, 75%; brsm 84%) as a white solid. Rf = 0.55 [ethyl acetate/methanol = 20/1 (v/v)]; ^1^H NMR (500 MHz, CDCl_3_) *δ* 7.51–7.48 (m, 2H, 2 × CH _PMB_), 7.39–7.34 (m, 3H, 3 × CH _Ph_), 7.31–7.27 (m, 2H, 2 × CH _Ph_), 6.89–6.85 (m, 2H, 2 × CH _PMB_), 5.44 (s, 1H, PhCH), 4.64 (dd, *J* = 26.0, 11.8 Hz, 2H, CH_2__PMB_), 4.48 (d, *J* = 8.2 Hz, 1H, H-1 _Gal_), 4.32 (d, *J* = 7.8 Hz, 1H, H-1 _Glc_), 4.27 (dd, *J* = 11.3, 1.3 Hz, 1H, H-6a _Gal_), 4.22 (br s, 1H, OH), 4.13 (d, *J* = 2.8 Hz, 1H, H-4 _Gal_), 4.06–3.94 (m, 3H, H-2 _Gal_, H-6b _Gal_, H-6a _Glc_), 3.93–3.82 (m, 2H, OC*H*H, H-6b _Glc_), 3.80 (s, 3H, CH_3 PMB_), 3.72–3.63 (m, 2H, H-4 _Glc_, H-5 _Glc_), 3.56–3.38 (m, 5H, H-3 _Gal_, H-5 _Gal_, OCH*H*, H-2 _Glc_, H-3 _Glc_), 3.26 (t, *J* = 6.9 Hz, 2H, CH_2_N_3_), 3.17 (br s, 1H, OH), 2.79 (br s, 1H, OH), 2.53 (br s, 1H, OH), 1.68–1.59 (m, 4H, 2 × CH_2_), 1.42–1.36 (m, 4H, 2×CH_2_); ^13^C NMR (125 MHz, CDCl_3_) *δ* 159.70 (C _PMB_), 137.56 (C _Benzylidene_), 129.79 (C _PMB_), 129.67, 129.23, 128.40 (129.67–128.40, 5 × CH _Ph_), 126.42 (2 × CH _PMB_), 114.15 (2 × CH _PMB_), 103.84 (C-1 _Gal_), 102.75 (C-1 _Glc_), 101.37 (PhCH), 80.58 (C-4 _Glc_), 79.02 (C-3 _Gal_), 74.85 (C-5 _Glc_), 74.79 (C-2 _Glc_), 73.79 (C-3 _Glc_), 72.60 (C-4 _Gal_), 71.37 (CH_2 PMB_), 70.16 (OCH_2_), 69.25, 69.22 (69.25–69.22, C-6 _Gal_, C-2 _Gal_), 67.25 (C-5 _Gal_), 62.22 (C-6 _Glc_), 55.45(CH_3 PMB_), 51.52 (CH_2_N_3_), 29.57 (CH_2_), 28.88 (CH_2_), 26.61 (CH_2_), 25.68 (CH_2_); HRMS (ESI) *m/z* calcd for C_33_H_45_N_3_O_12_Na [M + Na]^+^: 698.2896; found 698.2890.

### 6-Azidohexyl 3-*O*-Allyl-4,6-*O*-Benzylidene-*β*-D-Galactopyranosyl-(1→4)-*β*-D-Glucopyranoside (7b)

Benzaldehyde dimethyl acetal (0.1 ml, 0.69 mmol, 1.5 eq.) was added to a solution of compound **6b** (233 mg, 0.46 mmol, 1 eq.) in anhydrous acetonitrile (5.3 ml). Camphorsulfonic acid (21 mg, 0.09 mmol, 0.2 eq.) was then added to the prechilled reaction mixture at 0°C. The solution was stirred 10 min then was allowed to warm to room temperature. The reaction mixture was stirred for 2.5 h at room temperature and then quenched with triethylamine under an ice bath. The mixture was concentrated and purified by silica gel flash chromatography [ethyl acetate/methanol = 10/1 (v/v)] to afford compound **7b** (180 mg, 66%; brsm 70%) as a white solid. R_f_ = 0.5 [ethyl acetate/methanol = 10/1 (v/v)]; ^1^H NMR (500 MHz, CD_3_OD) *δ* 7.54–7.47 (m, 2H, 2 × CH _Ph_), 7.39–7.31 (m, 3H, 3 × CH _Ph_), 6.05–5.94 (m, 1H, CH _Allyl_), 5.62 (s, 1H, PhCH), 5.35 (dd, *J* = 17.3, 1.7 Hz, 1H, C*H*H _Allyl_), 5.17 (dd, *J* = 10.4, 1.2 Hz, 1H, CH*H*_Allyl_), 4.51 (d, *J* = 7.9 Hz, 1H, H-1 _Gal_), 4.40 (d, *J* = 3.4 Hz, 1H, H-4 _Gal_), 4.29 (d, *J* = 7.9 Hz, 1H, H-1 _Glc_), 4.27–4.14 (m, 4H, CH_2__Allyl_, H-6 _Gal_), 3.93–3.86 (m, 3H, OC*H*H, H-6 _Glc_), 3.76 (dd, *J* = 9.7, 8.0 Hz, 1H, H-2 _Gal_), 3.64–3.50 (m, 5H, OCH*H*, H-3 _Glc_, H-4 _Glc_, H-3 _Gal_, H-5 _Gal_), 3.41 (dt, *J* = 9.5, 3.1 Hz, 1H, H-5 _Glc_), 3.30–3.22 (m, 3H, CH_2_N_3,_ H-2 _Glc_), 1.67–1.57 (m, 4H, 2 × CH_2_), 1.47–1.38 (m, 4H, 2 × CH_2_); ^13^C NMR (125 MHz, CD_3_OD) *δ* 139.50 (C _Ph_), 136.30 (CH _Allyl_), 129.88, 129.04, 129.03, 127.37 (129.88–127.37, 5 × CH _Ph_), 117.57 (CH_2 Allyl_), 104.73 (C-1 _Gal_), 104.27 (C-1 _Glc_), 102.12 (PhCH), 80.40 (C-3 _Gal_), 80.19 (C-4 _Glc_), 76.41 (C-5 _Glc_), 76.34 (C-3 _Glc_), 74.81 (C-2 _Glc_), 74.26 (C-4 _Gal_), 71.73 (CH_2 Allyl_), 70.83 (C-2 _Gal_), 70.77 (OCH_2_), 70.25 (C-6 _Gal_), 68.25 (C-5 _Gal_), 61.78 (C-6 _Glc_), 52.40 (CH_2_N_3_), 30.60 (OCH_2_ *C*H_2_), 29.84 (*C*H_2_CH_2_N_3_), 27.59 (CH_2_), 26.62 (CH_2_); HRMS (ESI) *m/z* calcd for C_28_H_41_N_3_O_11_Na [M + Na]^+^: 618.2639; found 618.2641.

### 6-Azidohexyl 2-*O*-Benzyl-3-*O*-(-4-Methoxylbenzyl)-4,6-*O*-Benzylidine-*β*-D-Galactopyranosyl-(1→4)-2,3,6-Tri-*O*-Benzyl-*β*-D-Glucopyranoside (8a)

To a solution of compound **7a** (772 mg, 1.14 mmol, 1.0 eq.), anhydrous dimethylformamide (12.0 ml) and benzyl bromide (1.1 ml, 9.12 mmol, 8.0 eq.) were added. The solution was cooled to 0°C, and sodium hydride was added (60 wt% dispersion in mineral oil, 365 mg, 9.12 mmol, 8.0 eq.). The solution was stirred for 10 min and then was allowed to warm to room temperature. The reaction mixture was stirred for 1 h at room temperature and then quenched with methanol under an ice bath. The solution concentrated in *vacuo*, and the crude residue was diluted with a solution of ethyl acetate and water. The organic layer was separated and dried over magnesium sulfate, filtered, and then concentrated in *vacuo*. The residue was chromatographed on silica gel [ethyl acetate/hexane = 1/2 (v/v)] to afford compound **8a** (1.07 g, 88%) as white solid. R_f_ = 0.33 [ethyl acetate/hexane = 1/2 (v/v)]; ^1^H NMR (500 MHz, CDCl_3_) *δ* 7.54–7.14 (m, 27H, 25 × CH _Ph_, 2×CH _PMB_), 6.87–6.82 (m, 2H, 2 × CH _PMB_), 5.45 (s, 1H, PhCH), 5.17 (d, *J* = 10.7 Hz, 1H, C*H*H _Bn_), 4.90 (d, *J* = 11.0 Hz, 1H, C*H*H _Bn_), 4.83 (d, *J* = 11.2 Hz, 1H, C*H*H _Bn_), 4.78 (d, *J* = 10.7 Hz, 1H, CH*H*_Bn_), 4.75 (d, *J* = 10.8 Hz, 1H, CH_2__Bn_), 4.73 (d, *J* = 11.3 Hz, 1H, CH*H*_Bn_), 4.64 (t, *J* = 12.4 Hz, 2H, CH_2__PMB_), 4.54 (d, *J* = 12.1 Hz, 1H, C*H*H _Bn_), 4.46 (d, *J* = 7.9 Hz, 1H, H-1 _Glc_), 4.37 (d, *J* = 7.8 Hz, 1H, H-1 _Gal_), 4.34 (d, *J* = 12.1 Hz, 1H, CH*H*_Bn_), 4.20 (d, *J* = 11.5 Hz, 1H, H-6a _Gal_), 3.99 (d, *J* = 3.7 Hz, 1H, H-4 _Gal_), 3.98–3.90 (m, 2H, H-4 _Glc_, OC*H*H), 3.85–3.81 (m, 2H, H-6a _Glc_, H-6b _Gal_), 3.80 (s, 3H, CH_3 PMB_), 3.76–3.70 (m, 2H, H-2 _Gal_, H-6b _Glc_), 3.62 (t, *J* = 9.0 Hz, 1H, H-3 _Glc_), 3.55–3.48 (m, 1H, OCH*H*), 3.45–3.34 (m, 3H, H-2 _Glc_, H-3 _Gal_, H-5 _Glc_), 3.21 (t, *J* = 6.9 Hz, 2H, CH_2_N_3_), 2.94 (s, 1H, H-5 _Gal_), 1.69–1.60 (m, 2H, CH_2_), 1.57–1.52 (m, 2H, CH_2_), 1.45–1.33 (m, 4H, 2 × CH_2_); ^13^C NMR (125 MHz, CDCl_3_) *δ* 159.40 (C _PMB_), 139.14 (C _Ph_), 139.06 (C _Ph_), 138.90 (C _Ph_), 138.72 (C _Ph_), 138.26 (C _Benzylidene_), 130.59 (C _PMB_), 129.45, 128.98, 128.68, 128.42, 128.35, 128.33, 128.24, 128.22, 128.03, 127.88, 127.71, 127.66, 127.55, 127.51, 127.38, 126.72 (129.45–126.72, 25 × CH _Ph_, 2 × CH _PMB_), 113.89 (2 × CH _PMB_), 103.78 (C-1 _Glc_), 103.06 (C-1 _Gal_), 101.53 (PhCH), 83.21 (C-3 _Glc_), 82.01 (C-2 _Glc_), 79.53 (C-3 _Gal_), 78.97 (C-2 _Gal_), 77.87 (C-4 _Glc_), 75.88 (CH_2 Bn_), 75.39 (CH_2__Bn_), 75.27 (C-5 _Glc_), 75.07 (CH_2__Bn_), 73.88 (C-4 _Gal_), 73.12 (CH_2__Bn_), 71.47 (CH_2__PMB_), 69.93 (OCH_2_), 69.11 (C-6 _Gal_), 68.53 (C-6 _Glc_), 66.52 (C-5 _Gal_), 55.43 (CH_3 PMB_), 51.51 (CH_2_N_3_), 29.75 (CH_2_), 28.92 (CH_2_), 26.68 (CH_2_), 25.89 (CH_2_); HRMS (ESI) *m/z* calcd for C_61_H_69_N_3_O_12_Na [M + Na]^+^: 1058.4774; found 1058.4772.

### 6-Azidohexyl 2-*O*-Benzyl-3-*O*-Allyl-4,6-*O*-Benzylidene-*β*-D-Galactopyranosyl-(1→4)-*β*-D-Glucopyranoside (8b)

Benzyl bromide (290 μl, 2.4 mmol, 8.0 eq.) was added to a solution of compound **7b** (180 mg, 0.3 mmol, 1.0 eq.) in anhydrous dimethylformamide (3.2 ml). Sodium hydride (60 wt% dispersion in mineral oil, 96 mg, 2.4 mmol, 8.0 eq.) was then added to the prechilled reaction mixture at 0°C. The solution was stirred for 10 min and then was allowed to warm to room temperature. The reaction mixture was stirred for 2.5 h at room temperature and then quenched with methanol under an ice bath. The solution concentrated in *vacuo*; the crude residue was diluted with a solution of ethyl acetate and water. The organic layer was separated and dried over magnesium sulfate, filtered, and then concentrated in *vacuo*. The residue was chromatographed on silica gel [ethyl acetate/hexane = 1/3 (v/v)] to afford compound **8b** (249 mg, 87%) as white solid. R_f_ = 0.20 [ethyl acetate/hexane = 1/3 (v/v)]; ^1^H NMR (500 MHz, CDCl_3_) *δ* 7.55–7.50 (m, 2H, 2 × CH _Ph_), 7.49–7.45 (m, 2H, 2 × CH _Ph_), 7.38–7.27 (m, 18H, 18 × CH _Ph_), 7.22–7.17 (m, 3H, 3 × CH _Ph_),6.01–5.91 (m, 1H, CH _Allyl_), 5.52 (s, 1H, PhCH), 5.33 (dd, *J* = 17.3, 1.6 Hz, 1H, C*H*H _Allyl_), 5.20 (m, 2H, C*H*H _Allyl_, C*H*H _Bn_), 4.92 (d, *J* = 11.0 Hz, 1H, C*H*H _Bn_), 4.85–4.79 (m, 2H, C*H*H _Bn_, CH*H*_Bn_), 4.77 (d, *J* = 11.0 Hz, 1H, CH*H*_Bn_), 4.74 (d, *J* = 11.1 Hz, 1H, CH*H*_Bn_),4.57 (d, *J* = 12.2 Hz, 1H, C*H*H _Bn_), 4.51 (d, *J* = 7.9 Hz, 1H, H-1 _Gal_), 4.42–4.37 (m, 2H, CH*H*_Bn_, H-1 _Glc_), 4.26–4.18 (m, 3H, H-6a _Gal_, CH_2 Allyl_), 4.13 (d, *J* = 3.6 Hz, 1H, H-4 _Gal_), 4.01–3.92 (m, 2H, H-4 _Glc_, OC*H*H), 3.92–3.86 (m, 2H, H-6a _Glc_, H-6b _Gal_), 3.76–3.71 (m, 2H, H-6b _Glc_, H-2 _Gal_), 3.65 (t, *J* = 9.0 Hz, 1H, H-3 _Glc_), 3.57–3.51 (m, 1H, OCH*H*), 3.45 (dd, *J* = 9.0, 8.1 Hz, 1H, H-2 _Glc_), 3.42–3.34 (m, 2H, H-5 _Glc_, H-3 _Gal_), 3.22 (t, *J* = 7.0 Hz, 2H, CH_2_N_3_), 3.04 (s, 1H, H-5 _Gal_), 1.71–1.64 (m, 2H, CH_2_), 1.60–1.53 (m, 2H, CH_2_), 1.48–1.36 (m, 4H, 2 × CH_2_); ^13^C NMR (125 MHz, CDCl_3_) *δ* 139.13 (C _Ph_), 138.98 (C _Ph_), 138.87 (C _Ph_), 138.72 (C _Ph_), 138.20 (C _Ph_), 135.25 (CH _Allyl_), 128.96, 128.63, 128.39, 128.33, 128.33, 128.22, 128.18, 128.01, 127.90, 127.69, 127.63, 127.56, 127.51, 127.35, 126.71 (128.96–126.71, 25 × CH _Ph_), 117.19 (CH_2 Allyl_), 103.75 (C-1 _Glc_), 103.03 (C-1 _Gal_), 101.50 (PhCH), 83.20 (C-3 _Glc_), 81.99 (C-2 _Glc_), 79.85 (C-3 _Gal_), 78.94 (C-2 _Gal_), 77.87 (C-4 _Glc_), 75.85 (CH_2 Bn_), 75.38 (CH_2 Bn_), 75.25 (C-5 _Glc_), 75.04 (CH_2 Bn_), 74.07 (C-4 _Gal_), 73.10 (CH_2 Bn_), 71.29 (CH _Allyl_), 69.90 (OCH_2_), 69.11 (C-6 _Gal_), 68.50 (C-6 _Glc_), 66.51 (C-5 _Gal_), 51.47 (CH_2_N_3_), 29.73 (OCH_2_ *C*H_2_), 28.89 (*C*H_2_CH_2_N_3_), 26.65 (CH_2_), 25.86 (CH_2_); HRMS (ESI) *m/z* calcd for C_56_H_65_N_3_O_11_Na [M + Na]^+^: 978.4517; found 978.4510.

### 6-Azidohexyl 2,4-Di-*O*-Benzyl-3-*O*-(-4-Methoxylbenzyl)-*β*-D-Galactopyranosyl-(1→4)-2,3,6-Tri-*O*-Benzyl-*β*-D-Glucopyranoside (9a)

Borane tetrahydrofuran complex (1 M, 9.2 ml) was added to a mixture of compound **8a** (903 mg, 0.87 mmol, 1 eq.). The solution was cooled to −20°C and added 1 M TMSOTf in CH_2_Cl_2_ (438 μl, 0.44 mmol). After stirring at −20°C for 2 h, the reaction was quenched with triethylamine and methanol under an ice bath. The solution was concentrated in *vacuo*, the crude residue was diluted with a solution of ethyl acetate and 1N hydrochloric acid. The organic layer was separated and dried over magnesium sulfate, filtered, and then concentrated in *vacuo*. The residue was chromatographed on silica gel [ethyl acetate/hexane = 2/3 (v/v)] to afford compound **9a** (353 mg, 39%; brsm 67%) as colorless oil. R_f_ = 0.26 [ethyl acetate/hexane = 2/3 (v/v)].

Compound **9a** was alternatively prepared from **12**. Compound **12** (219 mg, 0.18 mmol, 1 eq.) was dissolved in tetrahydrofuran (1.6 ml). 1 M tetrabutylammonium fluoride in tetrahydrofuran solution (220 μl, 0.22 mmol, 1.2 eq.) was added under an ice bath, and the reaction was allowed to warm up to room temperature, stirred for 2 h. The solution was concentrated in *vacuo* and chromatographed on silica gel [ethyl acetate/hexane = 1/2 (v/v)] to afford compound **9a** (181 mg, 95%) as a colorless oil. ^1^H NMR (500 MHz, CDCl_3_) *δ* 7.37–6.85 (m, 29H, 25 × CH _Ph_, 4 x CH _PMB_), 5.02 (d, *J* = 10.9 Hz, 1H, C*H*H _Bn_), 4.96 (d, *J* = 11.6 Hz, 1H, C*H*H _Bn_), 4.90 (d, *J* = 11.1 Hz, 1H, C*H*H _Bn_), 4.84–4.78 (m, 2H, C*H*H _Bn_), 4.76 (d, *J* = 11.1 Hz, 1H, CH*H*_Bn_), 4.74 (d, *J* = 10.7 Hz, 1H, CH*H*_Bn_), 4.70–4.64 (m, 2H, CH_2__PMB_), 4.57 (d, *J* = 11.6 Hz, 1H, CH*H*_Bn_), 4.54 (d, *J* = 12.2 Hz, 1H, C*H*H _Bn_), 4.44–4.36 (m, 3H, CH*H*_Bn_, H-1 _Gal_, H-1 _Glc_), 3.97–3.91 (m, 1H, OC*H*H), 3.88 (t, *J* = 9.4 Hz, 1H, H-4 _Glc_), 3.82 (s, 3H, CH_3 PMB_), 3.81–3.73 (m, 3H, H-2 _Gal_, H-6 _Glc_), 3.67 (d, *J* = 2.6 Hz, 1H, H-4 _Gal_), 3.60–3.50 (m, 3H, OCH*H*, H-3 _Glc_, H-6a _Gal_), 3.43–3.37 (m, 3H, H-3 _Gal_, H-5 _Glc_, H-2 _Glc_), 3.31 (dd, *J* = 7.0, 4.5 Hz, 1H, H-6b _Gal_), 3.22 (t, *J* = 6.9 Hz, 2H, CH_2_N_3_), 3.19 (dd, *J* = 4.5, 3.3 Hz, 1H, H-5 _Gal_), 1.88 (br s, 1H, OH), 1.73–1.62 (m, 2H, CH_2_), 1.62–1.54 (m, 2H, CH_2_), 1.49–1.35 (m, 4H, 2 × CH_2_); ^13^C NMR (125 MHz, CDCl_3_) *δ* 159.35 (C _PMB_), 139.01 (C _Ph_), 138.93 (C _Ph_), 138.78 (C _Ph_), 138.74 (C _Ph_), 138.48 (C _Ph_), 130.63 (C _PMB_), 129.30, 128.45, 128.41, 128.35, 128.21, 128.17, 128.15, 128.09, 127.90, 127.82, 127.79, 127.68, 127.57, 127.55, 127.45 (129.30–127.45, 25 × CH _Ph_, 2 × CH _PMB_), 113.94 (2 × CH _PMB_), 103.71 (C-1 _Glc_), 103.04 (C-1 _Gal_), 82.91 (C-3 _Glc_), 82.58 (C-3 _Gal_), 81.76 (C-2 _Glc_), 80.02 (C-2 _Gal_), 77.19 (C-4 _Glc_), 75.68 (CH_2 Bn_), 75.32 (CH_2 Bn_), 75.25 (C-5 _Glc_), 75.14 (C-5 _Gal_), 74.97 (CH_2 Bn_), 74.54 (CH_2 Bn_), 73.95 (C-4 _Gal_), 73.28 (CH_2 Bn_), 72.84 (CH_2 PMB_), 69.83 (OCH_2_), 68.46 (C-6 _Glc_), 62.09 (C-6 _Gal_), 55.40 (CH_3 PMB_), 51.48 (CH_2_N_3_), 29.74 (CH_2_), 28.90 (CH_2_), 26.64 (CH_2_), 25.87 (CH_2_); HRMS (ESI) *m/z* calcd for C_61_H_71_N_3_O_12_Na [M + Na]^+^: 1060.4930; found 1060.4926.

### 6-Azidohexyl *O*-[2-*O*-Benzyl-3-*O*-(4-Methoxylbenzyl)-*β*-D-Galactopyranosyl]-(1→4)-2,3,6-Tri-*O*-Benzyl-*β*-D-Glucopyranoside (10)

Compound **8a** (257 mg, 0.25 mmol, 1 eq.) was dissolved in dichloromethane (2.4 ml) and methanol (2.4 ml). *p*-Toluenesulfonic acid (33 mg, 0.17 mmol, 0.7 eq.) and ethanethiol (110 μl, 1.49 mmol, 6 eq.) were added sequentially at 0°C. The ice bath was removed and stirred at room temperature for 6 h. The reaction was neutralized with triethylamine at 0°C, concentrated, and chromatographed on silica gel [ethyl acetate/hexane = 2/3 (v/v)] to afford compound **10** (203 mg, 86%) as a white powder. R_f_ = 0.37 [ethyl acetate/hexane = 1/1 (v/v)]; ^1^H NMR (400 MHz, CDCl_3_) *δ* 7.44–6.83 (m, 24H, 20 × CH _Ph_, 4 × CH _PMB_), 4.98 (d, *J* = 10.8 Hz, 1H, C*H*H _Bn_), 4.90 (d, *J* = 11.0 Hz, 1H, C*H*H _Bn_), 4.82–4.72 (m, 4H, C*H*H _Bn_, 3 × CH*H*_Bn_), 4.63–4.59 (m, 2H, CH_2__PMB_), 4.56 (d, *J* = 12.2 Hz, 1H, C*H*H _Bn_), 4.43–4.34 (m, 3H, CH*H*_Bn_, H-1 _Gal_, H-1 _Glc_), 3.99–3.87 (m, 2H, OC*H*H, H-4 _Glc_), 3.87–3.70 (m, 6H, H-4 _Gal_, CH_3 PMB_, H-6 _Glc_), 3.67–3.48 (m, 5H, OCH*H*, H-3 _Glc_, H-6a _Gal_, H-2 _Gal_, H-3 _Gal_), 3.44–3.36 (m, 2H, H-5 _Glc_, H-2 _Glc_), 3.32 (dd, *J* = 9.2, 2.9 Hz, 1H, H-6b _Gal_), 3.22 (t, *J* = 6.9 Hz, 2H, CH_2_N_3_), 3.19–3.13 (m, 1H, H-5 _Gal_), 2.19 (br s, 2H, 2 × OH), 1.73–1.61 (m, 2H, CH_2_), 1.61–1.52 (m, 2H, CH_2_), 1.49–1.33 (m, 4H, 2 × CH_2_); ^13^C NMR (100 MHz, CDCl_3_) *δ* 159.53 (C _PMB_), 139.06 (C _Ph_), 138.77 (C _Ph_), 138.75 (C _Ph_), 138.42 (C _Ph_), 130.03 (C _PMB_), 129.57, 128.43, 128.39, 128.36, 128.08, 128.01, 127.90, 127.83, 127.72, 127.65, 127.62 (129.57–127.62, 20 × CH _Ph_, 2 × CH _PMB_), 114.01 (2 × CH _PMB_), 103.74 (C-1 _Glc_), 102.74 (C-1 _Gal_), 82.89 (C-3 _Glc_), 81.82 (C-2 _Glc_), 80.84 (C-3 _Gal_), 79.34 (C-2 _Gal_), 75.68 (CH_2 Bn_), 75.35 (CH_2 Bn_), 75.20 (C-5 _Glc_), 74.99 (CH_2 Bn_), 74.14 (C-5 _Gal_), 73.30 (CH_2 Bn_), 72.01 (CH_2 PMB_), 69.87 (OCH_2_), 68.37 (C-6 _Glc_), 67.31 (C-4 _Gal_), 62.46 (C-6 _Gal_), 55.41 (CH_3 PMB_), 51.49 (CH_2_N_3_), 29.74 (CH_2_), 28.91 (CH_2_), 26.65 (CH_2_), 25.87 (CH_2_); HRMS (ESI) *m/z* calcd for C_54_H_65_N_3_O_12_Na [M + Na]^+^: 970.4466; found 970.4470.

### 6-Azidohexyl *O*-[2-*O*-Benzyl-3-*O*-(4-Methoxylbenzyl)-6-*O*-Triisopropylsilyl-*β*-D-Galactopyranosyl]-(1→4)-2,3,6-Tri-*O*-Benzyl-*β*-D-Glucopyranoside (11)

Compound **10** (203 mg, 0.21 mmol, 1 eq.) and imidazole (58 mg, 0.86 mmol, 4 eq.) were dissolved in anhydrous dichloromethane (2 ml), and triisopropylsilyl chloride (137 μl, 0.64 mmol, 3 eq.) was added dropwise at 0°C. The ice bath was removed and stirred at room temperature for 25 h. The mixture was extracted with dichloromethane and 1N hydrochloric acid. The organic phases were dried over magnesium sulfate and concentrated in *vacuo*. The residue was chromatographed on silica gel [ethyl acetate/hexane = 1/7 (v/v)] to afford compound **11** (228 mg, 96%) as a white powder. R_f_ = 0.33 [ethyl acetate/hexane = 1/5 (v/v)]; ^1^H NMR (400 MHz, CDCl_3_) *δ* 7.42–6.82 (m, 24H, 20 × CH _Ph_, 4 × CH _PMB_), 4.99 (d, *J* = 10.8 Hz, 1H, C*H*H _Bn_), 4.88 (d, *J* = 11.0 Hz, 1H, C*H*H _Bn_), 4.81–4.69 (m, 4H, C*H*H _Bn_, 3 × CH*H*_Bn_), 4.67–4.59 (m, 2H, CH_2__PMB_), 4.56 (d, *J* = 12.1 Hz, 1H, C*H*H _Bn_), 4.44–4.32 (m, 3H, CH*H*_Bn_, H-1 _Gal_, H-1 _Glc_), 4.03–3.89 (m, 3H, OC*H*H, H-4 _Glc_, H-4 _Gal_), 3.86–3.77 (m, 5H, CH_3 PMB_, H-6a _Glc_, H-6a _Gal_), 3.75–3.68 (m, 1H, H-6b _Glc_), 3.68–3.61 (dd, *J* = 9.4, 4.9 Hz, 1H, H-6b _Gal_), 3.60–3.47 (m, 3H, OCH*H*, H-3 _Glc_, H-2 _Gal_), 3.44–3.30 (m, 3H, H-5 _Glc_, H-2 _Glc_, H-3 _Gal_), 3.21 (t, *J* = 6.9 Hz, 2H, CH_2_N_3_), 3.15 (dd, *J* = 8.4, 4.9 Hz, 1H, H-5 _Gal_), 1.72–1.50 (m, 4H, 2 × CH_2_), 1.46–1.32 (m, 4H, 2 × CH_2_), 1.10–0.94 (m, 21H, 6 × CH_3 TIPS_, 3 × CH _TIPS_); ^13^C NMR (100 MHz, CDCl_3_) *δ* 159.45 (C _PMB_), 139.22 (C _Ph_), 138.95 (C _Ph_), 138.89 (C _Ph_), 138.51 (C _Ph_), 130.35 (C _PMB_), 129.48, 128.39, 128.18, 128.01, 127.86, 127.80, 127.65, 127.61, 127.36 (129.48–127.36, 20 × CH _Ph_, 2 × CH _PMB_), 113.98 (2 × CH _PMB_), 103.76 (C-1 _Glc_), 102.73 (C-1 _Gal_), 83.11 (C-3 _Glc_), 81.92 (C-2 _Glc_), 81.14 (C-3 _Gal_), 79.76 (C-2 _Gal_), 75.49 (CH_2 Bn_), 75.29 (C-5 _Glc_, CH_2 Bn_), 75.02 (CH_2 Bn_), 74.21 (C-5 _Gal_), 73.29 (CH_2 Bn_), 71.88 (CH_2 PMB_), 69.85 (OCH_2_), 68.46 (C-6 _Glc_), 65.45 (C-4 _Gal_), 61.20 (C-6 _Gal_), 55.38 (CH_3 PMB_), 51.50 (CH_2_N_3_), 29.74 (CH_2_), 28.91 (CH_2_), 26.66 (CH_2_), 25.88 (CH_2_), 18.13 (3 × CH_3 TIPS_), 18.10 (3 × CH_3 TIPS_), 12.00 (3 × CH _TIPS_); HRMS (ESI) *m/z* calcd for C_63_H_85_N_3_O_12_SiNa [M + Na]^+^: 1126.5795; found 1126.5805.

### 6-Azidohexyl *O*-[2,4-Di-*O*-Benzyl-3-*O*-(4-Methoxylbenzyl)-6-*O*-Triisopropylsilyl-*β*-D-Galactopyranosyl]-(1→4)-2,3,6-Tri-*O*-Benzyl-*β*-D-Glucopyranoside (12)

Benzyl bromide (125 μl, 1.05 mmol, 5 eq.) was added to a prechilled reaction mixture of compound **11** (227 mg, 0.21 mmol, 1 eq.) and sodium hydrate (60% wt%, dispersion in mineral oil, 42 mg, 1.05 mmol, 5 eq.) in anhydrous dimethylformamide (2.5 ml) at 0°C. The solution was stirred for 10 min then was allowed to warm to room temperature and stirred for 7 h at room temperature. The reaction was quenched with methanol, extracted with ethyl acetate and water, dried over magnesium sulfate, filtered, and then concentrated. The residue was chromatographed on silica gel [ethyl acetate/hexane = 1/12 (v/v)] to afford compound **12** (219 mg, 89%) as a colorless oil. R_f_ = 0.66 [ethyl acetate/hexane = 1/5 (v/v)]; ^1^H NMR (400 MHz, CDCl_3_) *δ* 7.41–6.82 (m, 29H, 25 × CH _Ph_, 4 x CH _PMB_), 5.05 (d, *J* = 10.6 Hz, 1H, C*H*H _Bn_), 5.01 (d, *J* = 11.5 Hz, 1H, C*H*H _Bn_), 4.89 (d, *J* = 11.0 Hz, 1H, C*H*H _Bn_), 4.85–4.72 (m, 3H, C*H*H _Bn_, 2 × CH*H*_Bn_), 4.71–4.60 (m, 4H, CH_2__PMB_, 2 × CH*H*_Bn_), 4.56 (d, *J* = 12.1 Hz, 1H, C*H*H _Bn_), 4.44–4.33 (m, 3H, CH*H*_Bn_, H-1 _Gal_, H-1 _Glc_), 3.97–3.89 (m, 3H, OC*H*H, H-4 _Glc_, H-4 _Gal_), 3.88–3.69 (m, 7H, CH_3 PMB_, H-6 _Glc_, H-6a _Gal_, H-2 _Gal_), 3.63–3.47 (m, 3H, H-6b _Gal_, OCH*H*, H-3 _Glc_), 3.44–3.31 (m, 3H, H-5 _Glc_, H-2 _Glc_, H-3 _Gal_), 3.21 (m, 3H, CH_2_N_3_, H-5 _Gal_), 1.71–1.50 (m, 4H, 2 × CH_2_), 1.47–1.32 (m, 4H, 2 × CH_2_), 1.10–0.93 (m, 21H, 6 × CH_3 TIPS_, 3 × CH _TIPS_); ^13^C NMR (100 MHz, CDCl_3_) *δ* 159.25 (C _PMB_), 139.56, 139.15, 138.96, 138.55 (139.56–138.55, 5×C _Ph_), 130.89 (C _PMB_), 129.24, 128.39, 128.38, 128.32, 128.25, 128.17, 128.10, 127.99, 127.89, 127.85,127.63, 127.56, 127.48, 127.23 (129.24–127.23, 25 × CH _Ph_, 2 × CH _PMB_), 113.86 (2 × CH _PMB_), 103.79 (C-1 _Glc_), 102.97 (C-1 _Gal_), 83.24 (C-3 _Glc_), 82.38 (C-3 _Gal_), 81.87 (C-2 _Glc_), 80.25 (C-2 _Gal_), 75.66 (CH_2 Bn_), 75.37 (C-5 _Glc_), 75.27 (CH_2 Bn_), 75.08 (CH_2 Bn_), 74.78 (C-5 _Gal_), 74.69 (CH_2 Bn_), 73.64 (C-4 _Gal_), 73.31 (CH_2 Bn_), 72.42 (CH_2 PMB_), 69.84 (OCH_2_), 68.41 (C-6 _Glc_), 60.94 (C-6 _Gal_), 55.37 (CH_3 PMB_), 51.50 (CH_2_N_3_), 29.75 (CH_2_), 28.92 (CH_2_), 26.67 (CH_2_), 25.88 (CH_2_), 18.20 (3 × CH_3 TIPS_), 18.17 (3 × CH_3 TIPS_), 11.99 (3 × CH _TIPS_); HRMS (ESI) *m/z* calcd for C_70_H_91_N_3_O_12_SiNa [M + Na]^+^: 1216.6264; found 1216.6271.

### 3,4,6-Tri-*O*-Acetyl-2-Deoxy-2-(2,2,2-Trichloroethoxycarbonylamino)-D-Glucopyranosyl-Trichloroacetimidate (13)

To a solution of 3,4,6-tri-*O*-acetyl-2-deoxy-2-(2,2,2-trichloroethoxycarbonylamino)-D-glucopyranoside (150 mg, 0.31 mmol, 1.0 eq.) in anhydrous dichloromethane (3.0 ml) was added trichloroacetonitrile (0.13 ml, 1.25 mmol, 4.0 eq.) and 1,8-diazabicyclo[5.4.0]undec-7-ene (10 μl, 0.06 mmol, 0.2 eq.). After being stirred for 8 h, the solution was concentrated in *vacuo*. The residue was chromatographed on silica gel [ethyl acetate/hexane = 1/3 (v/v)] to afford compound **13** (149 mg, 77%) as a white solid. R_f_ = 0.74 [ethyl acetate/hexane = 1/1 (v/v)]; ^1^H NMR (400 MHz, CDCl_3_) *δ* 8.80 (s, 1H, NH _imidate_), 6.42 (d, *J* = 3.7 Hz, 1H, H-1), 5.39–5.29 (dd, *J* = 10.2, 9.8 Hz, 1H, H-3), 5.28–5.18 (m, 2H, NH _Troc_, H-4), 4.76–4.67 (m, 2H, CH_2 Troc_), 4.77–4.68 (m, 2H, CH_2 Troc_), 4.34–4.24 (m, 2H, H-2, H-6a), 4.18–4.08 (m, 2H, H-5, H-6b), 2.07 (m, 9H, 3 × CH_3 Ac_). The ^1^H NMR spectral data were consistent with those available in the literature (Mitachi et al., 2014).

### 6-Azidohexyl 3,4,6-Tri-*O*-Acetyl-2-Deoxy-2-(2,2,2-Trichloroethoxycarbonylamino)-*β*-D-Glucopyranosyl-(1→6)-2,4-Di-*O*-Benzyl-3-*O*-(-4-Methoxylbenzyl)-*β*-D-Galactopyranosyl-(1→4)-2,3,6-Tri-*O*-Benzyl-*β*-D-Glucopyranoside (14)

A mixture of compound **9a** (703 mg, 0.68 mmol, 1.0 eq.), compound **13** (1.27 g, 2.03 mmol, 3 eq.), and 4 Å molecular sieves (0.5 g) in anhydrous dichloromethane (11.5 ml) was cooled to −20°C. Trimethylsilyl trifluoromethanesulfonate (25 μl, 0.14 mmol, 0.2 eq.) was added to the solution and stirred for 40 min. The reaction was quenched by trimethylamine under an ice bath. The solution was concentrated in *vacuo*; the crude residue was diluted with a solution of ethyl acetate and 1N hydrochloric acid. The organic layer was separated and dried over magnesium sulfate, filtered, and then concentrated in *vacuo*. The residue was chromatographed on silica gel [ethyl acetate/hexane = 2/5 (v/v)] to afford compound **14** (639 mg, 63%; brsm 78%) as a colorless oil. R_f_0.80 (ethyl acetate/hexane = 1/1); ^1^H NMR (500 MHz, CDCl_3_) *δ* 7.57–6.83 (m, 29H, 25 × CH _Bn_, 4 × CH _PMB_), 5.97 (d, *J* = 10.0 Hz, 1H, TrocNH), 5.12 (d, *J* = 12.6 Hz, 1H, C*H*H _Bn_), 5.06 (d, *J* = 11 Hz, 1H, C*H*H _Bn_), 5.01 (d, *J* = 12.7 Hz, 1H, CH*H*_Bn_), 4.97–4.89 (m, 3H, CH_2__Bn_, C*H*H _Troc_), 4.88–4.80 (m, 3H, H-4 _GlcNHTroc_, CH_2__Bn_), 4.71–4.60 (m, 3H, CH_2 PMB_, H-3 _GlcNAc_), 4.53 (d, *J* = 12.1 Hz, 1H, C*H*H _Bn_), 4.47 (d, *J* = 11.0 Hz, 1H, CH*H*_Bn_), 4.45–4.38 (m, 2H, H-1 _Gal_, CH*H*_Bn_), 4.33 (d, *J* = 7.9 Hz, 1H, H-1 _Glc_), 4.23 (d, *J* = 12.3 Hz, 1H, CH*H*_Troc_), 4.11 (t, *J* = 9.3 Hz, 1H, H-4 _Glc_), 4.02 (dd, *J* = 12.3, 3.9 Hz, 1H, H-6a _GlcNHTroc_), 3.96 (d, *J* = 10.2 Hz, 1H, H-6a _Glc_), 3.91–3.84 (m, 1H, OC*H*H), 3.83–3.71 (m, 7H, CH_3 PMB_, H-2 _Glc_, H-2’, H-6b _GlcNHTroc_, H-2 _GlcNHTroc_), 3.70–3.62 (m, 4H, H-1 _GlcNHTroc_, H-6b _Glc_, H6 _Gal_), 3.61–3.53 (m, 2H, H-3 _Glc_, H-4 _Gal_), 3.49–3.42 (m, 1H, OCH*H*), 3.39–3.32 (m, 2H, H-3 _Gal_, H-5 _Gal_), 3.27 (d, *J* = 9.7 Hz, 1H, H-5 _Glc_), 3.22 (t, *J* = 6.9 Hz, 2H, CH_2_N_3_), 2.26 (d, *J* = 9.1 Hz, 1H, H-5 _GlcNHTroc_), 2.04 (s, 3H, CH_3 Ac_), 2.00 (s, 3H, CH_3 Ac_), 1.94 (s, 3H, CH_3 Ac_), 1.61–1.52 (m, 4H, 2 × CH_2_), 1.44–1.31 (m, 4H, 2 × CH_2_); ^13^C NMR (125 MHz, CDCl_3_) *δ* 170.93 (C _Ac_), 170.29 (C _Ac_), 169.42 (C _Ac_), 159.40 (C _PMB_), 154.81 (C _Troc_), 140.00 (C _Ph_), 139.31 (C _Ph_), 138.88 (C _Ph_), 138.71 (C _Ph_), 138.22 (C _Ph_), 130.51 (C _PMB_), 129.32, 128.80, 128.57, 128.48, 128.44, 128.39, 128.37, 128.23, 128.19, 127.99, 127.85, 127.80, 127.61, 127.56, 127.45 (129.32–127.45, 25 × CH _Ph_, 2 × CH _PMB_), 113.97 (2 × CH _PMB_), 104.25 (C-1 _Glc_), 103.26 (C-1 _Gal_), 102.48 (C-1 _GlcNHTroc_), 96.22 (CCl_3 Troc_), 85.56 (C-3 _Glc_), 82.49 (C-3 _Gal_), 81.60 (C-2 _Glc_), 79.88 (C-2 _Gal_), 76.61 (CH_2 Bn_), 76.42 (C-4 _Glc_), 75.43 (CH_2 Bn_), 75.22 (C-5 _Glc_), 75.06 (CH_2 Bn_), 74.81 (C-4 _Gal_), 74.70 (CH_2 Bn_), 74.14 (C-5 _Gal_), 73.84 (CH_2 Troc_), 73.02 (CH_2 PMB_), 72.83 (CH_2 Bn_), 72.57 (C-3 _GlcNHTroc_), 70.64 (C-5 _GlcNHTroc_), 70.21 (C-6 _Gal_), 69.76 (OCH_2_), 68.15 (C-4 _GlcNHTroc_), 67.90 (C-6 _Glc_), 61.82 (C-6 _GlcNHTroc_), 55.73 (C-2 _GlcNHTroc_), 55.42 (CH_3 PMB_), 51.52 (CH_2_N_3_), 29.80 (CH_2_), 28.91 (CH_2_), 26.66 (CH_2_), 25.91 (CH_2_), 20.89 (CH_3 Ac_), 20.78 (2×CH_3 Ac_); HRMS (ESI) *m/z* calcd for C_76_H_89_Cl_3_N_3_O_12_Na [M + Na]^+^: 1521.4977; found 1521.4979.

### 6-Azidohexyl 3,4,6-Tri-*O*-Acetyl-2-Acetamido-2-Deoxy-*β*-D-Glucopyranosyl-(1→6)-2,4-Di-*O*-Benzyl-3-*O*-(-4-Methoxylbenzyl)-*β*-D-Galactopyranosyl-(1→4)-2,3,6-Tri-*O*-Benzyl-*β*-D-Glucopyranoside (15)

Compound **14** (639 mg, 0.43 mmol, 1 eq.) was dissolved in a mixture of tetrahydrofuran/dioxane/water = 4:2:1 (v/v/v, 14 ml), and then lithium hydroxide (102 mg, 4.26 mmol, 10 eq.) was added. The mixture was stirred at room temperature for 21 h. The solution was neutralized by adding 1N hydrochloric acid and then concentrated in *vacuo*. The crude residue was treated with pyridine (20.7 ml) and acetic anhydride (1.8 ml, 19.17 mmol, 45 eq.). The mixture was stirred at room temperature for 24 h and then quenched with methanol under an ice bath. The solution concentrated in *vacuo*, the crude residue was diluted with a solution of ethyl acetate and 1N hydrochloric acid. The organic layer was separated and dried over magnesium sulfate, filtered, and then concentrated in *vacuo*. The residue was chromatographed on silica gel [ethyl acetate/hexane = 2/3 (v/v)] to afford compound **15** (344 mg, 59%, two steps) as white solid. R_f_ = 0.69 [ethyl acetate/hexane = 3/1 (v/v)]; ^1^H NMR (500 MHz, CDCl_3_) *δ* 7.44–6.83 (m, 29H, 25 × CH _Bn_, 4 × CH _PMB_), 5.67 (d, *J* = 9.3 Hz, 1H, NHAc), 5.12 (d, *J* = 11.7 Hz, 1H, C*H*H _Bn_), 5.02 (d, *J* = 11.0 Hz, 1H, C*H*H _Bn_), 5.00 (d, *J* = 11.0 Hz, 1H, C*H*H _Bn_), 4.94 (t, *J* = 10.0 Hz, 1H, H-4 _GlcNAc_), 4.90 (d, *J* = 11 Hz, 1H, CH*H*_Bn_), 4.84–4.73 (m, 4H, H-3 _GlcNAc_, CH*H*_Bn,_ CH_2__Bn_), 4.69–4.63 (m, 2H, CH_2 PMB_), 4.56–4.49 (m, 2H, C*H*H _Bn,_ CH*H*_Bn_), 4.43–4.36 (m, 3H, H-1 _Gal_, CH*H*_Bn_, H-1 _Glc_), 4.08 (d, *J* = 8.5 Hz, 1H, H-1 _GlcNAc_), 4.03 (dd, *J* = 19.3, 9.3 Hz, 1H, H-2 _GlcNAc_), 3.98–3.89 (m, 3H, OC*H*H, H-4 _Glc_, H-6a _GlcNAc_), 3.86 (dd, *J* = 10.7, 10.7 Hz, 1H, H-6a _Glc_), 3.81 (s, 3H, CH_3 PMB_), 3.76 (dd, *J* = 9.7, 8.0 Hz, 1H, H-2 _Gal_), 3.71–3.62 (m, 5H, H-6 _Gal_, H-6b _GlcNAc_, H-6b _Glc_, H-4 _Gal_), 3.59 (t, *J* = 9.2 Hz, 1H, H-3 _Glc_), 3.55–3.48 (m, 1H, OCH*H*), 3.45 (dd, *J* = 9.1, 7.8 Hz, 1H, H-2 _Glc_), 3.37–3.29 (m, 3H, H-3 _Gal_, H-5 _Gal_, H-5 _Glc_), 3.21 (t, *J* = 6.9 Hz, 2H, CH_2_N_3_), 2.45 (d, *J* = 9.8 Hz, 1H, H-5 _GlcNAc_), 2.01 (s, 3H, CH_3 Ac_), 1.98 (s, 3H, CH_3 Ac_), 1.94 (s, 3H, CH_3 Ac_), 1.91 (s, 3H, CH_3 Ac_), 1.69–1.63 (m, 2H, CH_2_), 1.57–1.51 (m, 2H, CH_2_), 1.44–1.36 (m, 4H, 2 × CH_2_); ^13^C NMR (125 MHz, CDCl_3_) *δ* 170.97 (C _Ac_), 170.86 (C _Ac_), 170.43 (C _Ac_), 169.30 (C _Ac_), 159.40 (C _PMB_), 139.67 (C _Ph_), 139.06 (C _Ph_), 138.89 (C _Ph_), 138.75 (C _Ph_), 138.07 (C _Ph_), 130.55 (C _PMB_), 129.30, 128.62, 128.48, 128.46, 128.38, 128.29, 128.23, 128.10, 127.90, 127.88, 127.84, 127.82, 127.76, 127.62, 127.54 (129.30–127.54, 25 × CH _Ph_, 2 × CH _PMB_), 113.99 (2 × CH _PMB_), 103.99 (C-1 _Glc_), 102.98 (C-1 _Gal_), 101.58 (C-1 _GlcNAc_), 84.33 (C-3 _Glc_), 82.51 (C-2 _Glc_), 82.32 (C-3 _Gal_), 79.79 (C-2 _Gal_), 76.67 (C-4 _Glc_), 76.38 (CH_2 Ph_), 75.38 (CH_2 Ph_), 75.02 (2×CH_2 Ph_), 74.97 (C-5 _Glc_), 74.82 (C-4 _Gal_), 74.61 (C-5 _Gal_), 73.45 (C-3 _GlcNAc_), 73.24 (CH_2 Ph_), 72.91 (CH_2 PMB_), 70.76 (C-5 _GlcNAc_), 69.89 (OCH_2_), 69.09 (C-6 _Gal_), 68.05 (C-4 _GlcNAc_), 67.83 (C-6 _Glc_), 61.68 (C-6 _GlcNAc_), 55.43 (CH_3 PMB_), 53.70 (C-2 _GlcNAc_), 51.49 (CH_2_N_3_), 29.72 (CH_2_), 28.92 (CH_2_), 26.65 (CH_2_), 25.83 (CH_2_), 23.71 (CH_3 Ac_), 20.88 (CH_3 Ac_), 20.83 (CH_3 Ac_), 20.73 (CH_3 Ac_); HRMS (ESI) *m/z* calcd for C_75_H_90_N_4_O_20_Na [M + Na]^+^: 1389.6041; found 1389.6033.

### 6-Azidohexyl 3,4,6-Tri-*O*-Acetyl-2-Acetamido-2-Deoxy-*β*-D-Glucopyranosyl-(1→6)-2,4-Di-*O*-Benzyl-*β*-D-Galactopyranosyl-(1→4)-2,3,6-Tri-*O*-Benzyl-*β*-D-Glucopyranoside (16)

Ceric ammonium nitrate (18 mg, 0.034 mmol, 2 eq.) was added to a solution of compound **15** (23 mg, 0.017 mmol, 1 eq.) in acetonitrile (332 μl) and water (33 μl). The reaction mixture was stirred at room temperature for 1 h. The solution concentrated in *vacuo*; the crude residue was diluted with a solution of dichloromethane and water. The organic layer was separated and dried over magnesium sulfate, filtered, and then concentrated in *vacuo*. The residue was chromatographed on silica gel [ethyl acetate/hexane = 7/4 (v/v)] to afford compound **16** (14 mg, 67%) as white solid. R_f_ = 0.18 [ethyl acetate/hexane = 3/2 (v/v)]; ^1^H NMR (500 MHz, CDCl_3_) *δ* 7.46–7.11 (m, 25H, 25 × CH _Ph_), 5.69 (d, *J* = 9.5 Hz, 1H, NHAc), 5.11 (d, *J* = 11.7 Hz, 1H, C*H*H _Bn_), 5.00 (d, *J* = 11.0 Hz, 1H, C*H*H _Bn_), 4.96 (t, *J* = 9.7 Hz, 1H, H-4 _GlcNAc_), 4.90 (d, *J* = 11.0 Hz, 1H, CH*H*_Bn_), 4.88–4.78 (m, 4H, H-3 _GlcNAc_, CH*H*_Bn_, 2 × C*H*H _Bn_), 4.67 (d, *J* = 11.5 Hz, 1H, CH*H*_Bn_), 4.58 (dd, *J* = 11.2, 3.0 Hz, 1H, CH*H*_Bn_), 4.59 (d, *J* = 11.1 Hz, 1H, C*H*H _Bn_), 4.45 (d, *J* = 12.3 Hz, 1H, CH*H*_Bn_), 4.41 (t, *J* = 8.2 Hz, 2H, H-1 _Gal_, H-1 _Glc_), 4.14 (d, *J* = 8.5 Hz, 1H, H-1 _GlcNAc_), 4.05 (t, *J* = 9.6 Hz, 1H, H-2 _GlcNAc_), 4.00–3.95 (m, 2H, H-6a _GlcNAc_, H-4 _Glc_), 3.95–3.90 (m, 1H, OC*H*H), 3.84 (dd, *J* = 10.8, 10.8 Hz, 1H, H-6a _Glc_), 3.76 (dd, *J* = 12.0, 12.0 Hz, 1H, H-6a _Gal_), 3.73–3.67 (m, 3H, H-6b _Gal_, H-6b _GlcNAc_, H-6b _Glc_), 3.66–3.50 (m, 4H, H-3 _Glc_, H-4 _Gal_, OCH*H*, H-2 _Gal_), 3.50–3.41 (m, 3H, H-5 _Gal_, H-2 _Glc_, H-3 _Gal_), 3.35 (d, *J* = 9.9 Hz, 1H, H-5 _Glc_), 3.21 (t, *J* = 6.9 Hz, 2H, CH_2_N_3_), 2.60–2,56 (m, 1H, H-5 _GlcNAc_), 2.24 (br s, 1H, OH), 2.02–1.90 (m, 12H, 4 × CH_3 Ac_), 1.68–1.61 (m, 2H, CH_2_), 1.59–1.52 (m, 2H, CH_2_), 1.46–1.34 (m, 4H, 2 × CH_2_); ^13^C NMR (125 MHz, CDCl_3_) *δ* 170.95 (C _Ac_), 170.90 (C _Ac_), 170.40 (C _Ac_), 169.29 (C _Ac_), 139.54 (C _Ph_), 139.01 (C _Ph_), 138.52 (C _Ph_), 138.35 (C _Ph_), 137.91 (C _Ph_), 128.68, 128.66, 128.56, 128.49, 128.46, 128.24, 128.16, 128.08, 128.04, 127.89, 127.76, 127.61 (128.68–127.61, 25 × CH _Ph_), 103.98 (C-1 _Glc_), 102.88 (C-1 _Gal_), 101.51 (C-1 _GlcNAc_), 84.18 (C-3 _Glc_), 82.52 (C-2 _Glc_), 80.10 (C-2 _Gal_), 76.62 (C-4 _Glc_), 76.42 (C-4 _Gal_), 76.31 (CH_2 Ph_), 75.45 (CH_2__Ph_), 75.11 (CH_2__Ph_), 75.00 (CH_2__Ph_), 74.94 (C-5 _Glc_), 74.78 (C-5 _Gal_), 74.36 (C-3 _Gal_), 73.35 (C-3 _GlcNAc_), 73.30 (CH_2__Ph_), 70.88 (C-5 _GlcNAc_), 69.91 (OCH_2_), 68.63 (C-6 _Gal_), 68.07 (C-4 _GlcNAc_), 67.87 (C-6 _Glc_), 61.70 (C-6 _GlcNAc_), 53.77 (C-2 _GlcNAc_), 51.48 (CH_2_N_3_), 29.71 (CH_2_), 28.91 (CH_2_), 26.64 (CH_2_), 25.83 (CH_2_), 23.68, 20.85, 20.82, 20.73 (23.68–20.73, 4 × CH_3 Ac_); HRMS (ESI) *m/z* calcd for C_67_H_82_N_4_O_19_Na [M + Na]^+^: 1269.5466; found 1269.5460.

### 6-Azidohexyl *O*-[3,4,6-Tri-*O*-Acetyl-2-Deoxy-2-(2,2,2-Trichloroethoxycarbonylamino)-*β*-D-Glucopyranosyl]-(1→3)-(2-acetamido-3,4,6-Tri-*O*-Acetyl-2-Deoxy-*β*-D-Glucopyranosyl)-(1→6)-(2,4-Di-*O*-Benzyl-*β*-D-Galactopyranosyl)-(1→4)-2,3,6-Tri-*O*-Benzyl-*β*-D-Glucopyranoside (17)

A mixture of compound **16** (151 mg, 0.12 mmol, 1.0 eq.), compound **13** (1.27 g, 2.03 mmol, 3 eq.), and 4 Å molecular sieves (0.150 g) in anhydrous dichloromethane (2.4 ml) was cooled to −20°C. The solution was added to trimethylsilyl trifluoromethanesulfonate (4.5 μl, 0.024 mmol, 0.2 eq.) and stirred for 3.5 h. The reaction was quenched by trimethylamine under an ice bath. The solution concentrated in *vacuo*; the crude residue was diluted with a solution of ethyl acetate and 1N hydrochloric acid. The organic layer was separated and dried over magnesium sulfate, filtered, and then concentrated in *vacuo*. The residue was chromatographed on silica gel [ethyl acetate/hexane = 2/1 (v/v)] to afford compound **17** (291 mg, 55%) as a colorless oil. R_f_ = 0.38 [ethyl acetate/hexane = 2/1 (v/v)]; ^1^H NMR (500 MHz, CDCl_3_) *δ* 7.54–7.05 (m, 25H, 25 × CH _Ph_), 5.68 (d, *J* = 9.4 Hz, 1H, NHAc), 5.10 (d, *J* = 11.7 Hz, 1H, C*H*H _Bn_), 5.06–4.97 (m, 3H, 2 × C*H*H _Bn_, H-4 _GlcNHTroc_), 4.97–4.85 (m, 3H, C*H*H _Bn_, CH*H*_Bn_, H-4 _GlcNAc_), 4.85–4.74 (m, 3H, CH*H*_Bn_, H-3 _GlcNAc_, H-3 _GlcNHTroc_), 4.72 (d, *J* = 8.2 Hz, 1H, H-1 _GlcNHTroc_), 4.67 (d, *J* = 12.2 Hz, 1H, C*H*H _Bn_), 4.64–4.55 (m, 2H, CH*H*_Bn_, C*H*H _Troc_), 4.54–4.44 (m, 2H, CH*H*_Troc_, CH*H*_Bn_), 4.44–4.39 (m, 2H, CH*H*_Bn_, H-1 _Gal_), 4.36 (d, *J* = 7.7 Hz, 1H, H-1 _Glc_), 4.28 (dd, *J* = 12.4, 4.9 Hz, 1H, H-6b _GlcNHTroc_), 4.22 (dd, *J* = 12.2, 1.8 Hz, 1H, H-6a _GlcNHTroc_), 4.07 (d, *J* = 8.6 Hz, 1H, H-1 _GlcNAc_), 4.03–3.95 (m, 3H, H-6b _GlcNAc_, H-2 _GlcNAc_, H-4 _Glc_), 3.94–3.85 (m, 1H, OC*H*H), 3.85–3.62 (m, 9H, H-6 _Glc_, H-2 _GlcNHTroc_, H-2 _Gal_, H-6 _Gal_, H-6a _GlcNAc_, H-4 _Gal_, H-5 _GlcNHTroc_), 3.61–3.54 (m, 2H, H-3 _Glc_, H-3 _Gal_), 3.54–3.40 (m, 3H, OCH*H*, H-5 _Gal_, H-2 _Glc_), 3.26 (d, *J* = 10.0 Hz, 1H, H-5 _Glc_), 3.23–3.16 (m, 2H, CH_2_N_3_), 2.42–2.35 (m, 1H, H-5 _GlcNAc_), 2.06–2.02 (m, 9H, 3 × CH_3__Ac_), 2.00–1.92 (m, 12H, 4 × CH_3__Ac_), 1.68–1.58 (m, 2H, CH_2_), 1.58–1.49 (m, 2H, CH_2_), 1.46–1.32 (m, 4H, 2 × CH_2_); ^13^C NMR (125 MHz, CDCl_3_) *δ* 170.98 (C _Ac_), 170.79 (C _Ac_), 170.68 (C _Ac_), 170.55 (C _Ac_), 170.41 (C _Ac_), 169.53 (C _Ac_), 169.29 (C _Ac_), 154.21 (C _Troc_), 139.59 (C _Ph_), 139.05 (C _Ph_), 138.75 (C _Ph_), 138.65 (C _Ph_), 138.06 (C _Ph_), 128.90, 128.73, 128.51, 128.48, 128.40, 128.34, 128.19, 128.15, 128.05, 128.03, 127.93, 127.74, 127.61, 126.92 (128.90–126.92, 25 × CH _Ph_), 103.98 (C-1 _Glc_), 102.64 (C-1 _Gal_), 101.80 (C-1 _GlcNHTroc_), 101.44 (C-1 _GlcNAc_), 95.56 (CCl_3_), 84.37 (C-3 _Glc_), 82.45 (C-2 _Glc_), 80.61 (C-3 _Gal_, C-2 _Gal_), 76.76 (C-4 _Gal_), 76.43 (CH_2__Bn_), 76.28 (C-4 _Glc_), 75.36 (CH_2__Bn_), 74.94 (2×CH_2__Bn_), 74.84 (C-5 _Glc_), 74.52 (CH_2__Troc_), 74.41 (C-5 _Gal_), 73.49 (CH_2__Bn_), 73.42 (C-3 _GlcNAc_), 72.07 (C-3 _GlcNHTroc_, C-5 _GlcNHTroc_), 70.74 (C-5 _GlcNAc_), 69.88 (OCH_2_), 68.79 (C-6 _Gal_, C-4 _GlcNHTroc_), 68.03 (C-4 _GlcNAc_), 67.75 (C-6 _Glc_), 62.20 (C-6 _GlcNHTroc_), 61.69 (C-6 _GlcNAc_), 56.38 (C-2 _GlcNHTroc_), 53.85 (C-2 _GlcNAc_), 51.48 (CH_2_N_3_), 29.68 (CH_2_), 28.90 (CH_2_), 26.63 (CH_2_), 25.81 (CH_2_), 23.74 (CH_3 Ac_), 20.95 (CH_3 Ac_), 20.89 (CH_3 Ac_), 20.83 (CH_3 Ac_), 20.76 (CH_3 Ac_), 20.75 (CH_3 Ac_), 20.68 (CH_3 Ac_); HRMS (ESI) *m/z* calcd for C_82_H_100_N_5_O_28_Cl_3_Na [M + Na]^+^: 1730.5513; found 1730.5508.

## Conclusion

In summary, we achieved the first chemoenzymatic synthesis of LNH by using selectively enzymatic glycosylation on the core tetrasaccharide as part of a substrate-controlled glycan extension strategy. This approach employed a branched tetrasaccharide scaffold in which a *β*-GlcNAc at the C6′-OH and a *β*-GlcNH_2_ at the C3′-OH of the lactoside were assembled. The inherent substrate preference of **HP0826** led to the enzymatic *β*1,4-galactosylation of GlcNAc at the C6-arm, and subsequent enzymatic *β*1,3-galactosylation on the C3-arm by **WbgO** completed the LNH synthesis. We expect that sufficient quantities of branched HMOs will increase the number of practical applications of these critical biomolecules. Our strategy represents a promising approach for preparative-scale production and has a high potential for diversification to achieve a panel of fucosylated or sialylated branched HMOs. We intend to develop a library of structurally complex asymmetrically branched HMOs and bioactivity evaluations in the future.

## Data Availability

The original contributions presented in the study are included in the article/Supplementary Material, further inquiries can be directed to the corresponding author.
