# Supplementary material for: Chemoenzymatic Synthesis of Asymmetrically Branched Human Milk Oligosaccharide Lacto-N-Hexaose
Source: Front Chem. 2022 May 31;10:905105. doi: 10.3389/fchem.2022.905105 (PMC9194828; doi:10.3389/fchem.2022.905105)
Supplement: Supplementary file 1 [file DataSheet1.pdf]

Supplementary Material

Chemoenzymatic Synthesis of Asymmetrically Branched Human Milk Oligosaccharide Lacto-*N*-hexaose

Kai-Eng Ooi,[1] Xiu-Wen Zhang,[1] Cheng-Yu Kuo,[1] Ying-Jia Liu,[1] and Ching-Ching Yu[1,2]*

[1] Department of Chemistry and Biochemistry, National Chung Cheng University, 168 University Road, Min-Hsiung, Chiayi 62102, Taiwan

[2] Institute of Biological Chemistry, Academia Sinica, 128, Academia Road Sec. 2, Nankang, Taipei 11529, Taiwan

*To whom correspondence should be addressed.

E-mail: checcyu@ccu.edu.tw

Tel: +886-5-2729287; Fax: +886-5-2721040

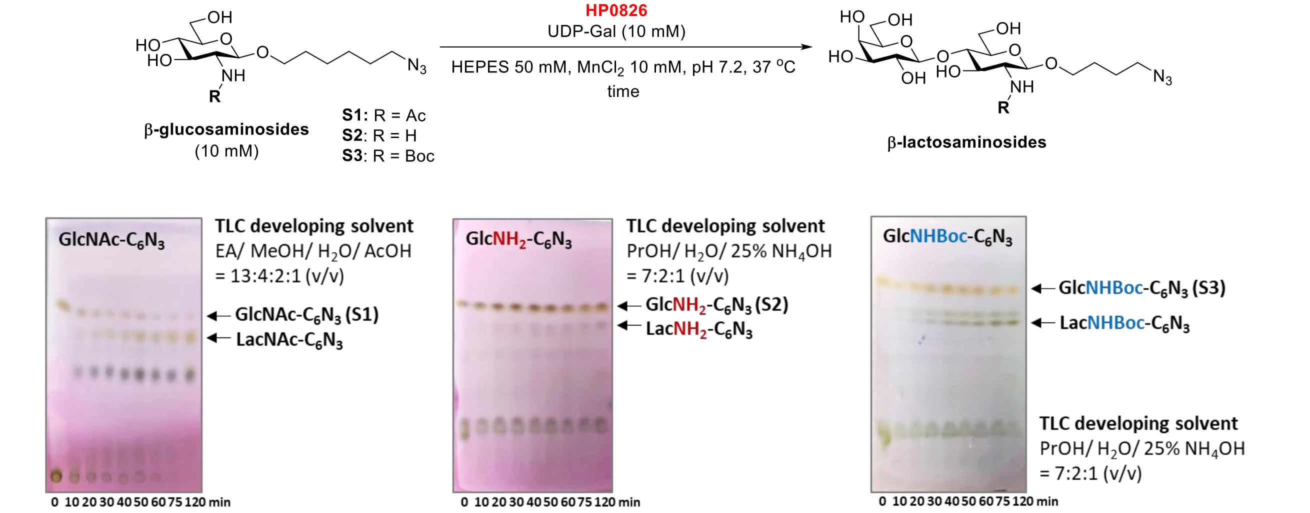

Supplementary Figure 1. Evaluation of HP0826 activity on varying β-glucosaminosides. Reaction of HP0826- catalyzed β1,4-galactosylation on β-glucosaminosides with different *N*-modifications (top); TLC analysis of HP0826-catalyzed β1,4-galactosylation on different substrates (GlcNAc-C_6N_3 (**S1**), $GlcNH_2$-C_6N_3 (**S2**) and GlcNHBoc-C_6N_3 (**S3**)) in the presence of 1 equivalent of UDP-Gal (bottom).

Synthetic Procedures and Characterization

Synthesis of β-glucosaminosides with N-modification

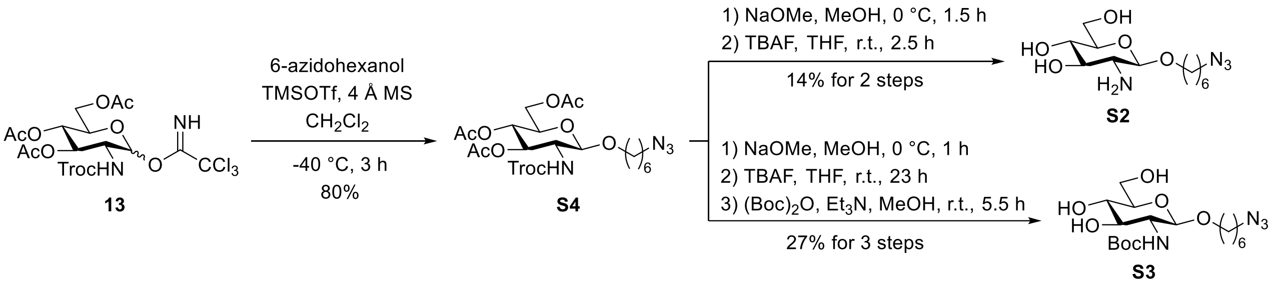

Supplementary Scheme 1. Synthesis of β-glucosaminosides.

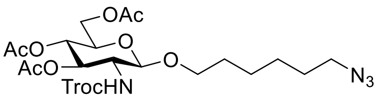

6-Azidohexyl 3,4,6-tri-*O*-acetyl-2-deoxy-2-(2,2,2-trichloroethoxycarbonylamino)-β-D-glucopyranoside (S4). A mixture of compound **13** (520 mg, 0.83 mmol, 1.0 eq.), 6-azido-1-hexanol (348 μL, 2.49 mmol, 3 eq.) and 4 Å molecular sieves (0.8 g) in anhydrous dichloromethane (8.3 mL) was cooled to −40 °C. The solution was added trimethylsilyl trifluoromethanesulfonate (93 μL, 0.54

mmol, 0.65 eq.) and stirred for 3 h. The reaction was quenched by trimethylamine under ice bath. The solution concentrated in *vacuo*, the crude residue was diluted with a solution of ethyl acetate and 1N hydrochloric acid. The organic layer was separated and dried over magnesium sulfate, filtered, and then concentrated in *vacuo*. The residue was chromatographed on silica gel (ethyl acetate/ hexane = 2/5 (v/v)) to afford compound **S4** (402 mg, 80%) as the white powder. R_f = 0.41 (ethyl acetate/ dichloromethane = 1/5 (v/v)); ^{1}H NMR (500 MHz, $CDCl_3$) δ 5.34-5.22 (m, 1H, H-3), 5.05 (t, *J* = 9.8 Hz, 1 H, H-4), 4.76 (d, *J* = 11.9 Hz, 1 H, C*H*H $_{Troc}$), 4.66 (d, *J* = 12.1 Hz, 1 H, CH*H* $_{Troc}$), 4.62 (d, *J* = 8.2 Hz, 1 H, H-1), 4.26 (dd, *J* = 12.3, 4.8 Hz, 1 H, H-6b), 4.12 (dd, *J* = 12.3, 2.2 Hz, 1 H, H-6a), 3.90-3.84 (m, 1H, OC*H*H), 3.72-3.66 (m, 1H, H-5), 3.65-3.56 (m, 1H, H-2), 3.51-3.44 (m, 1H, OCH*H*), 3.25 (t, *J* = 6.9 Hz, 1 H, CH_2N_3), 2.07 (s, 3 H, $CH_{3\ Ac}$), 2.01 (s, 3 H, $CH_{3\ Ac}$), 1.63-1.52 (m, 4 H, 2×CH_2), 1.40-1.30 (m, 4 H, 2×CH_2); ^{13}C NMR (125 MHz, $CDCl_3$) δ 170.84 (C_{Ac}), 170.79 (C_{Ac}), 169.61 (C_{Ac}), 154.16 (C_{Troc}), 100.91 (C-1), 95.59 (CCl_3), 74.56 ($CH_{2\ Troc}$), 72.04 (C-3), 71.87 (C-5), 70.11 (OCH_2), 68.87 (C-4), 62.24 (C-6), 56.44 (C-2), 51.43 (CH_2N_3), 29.38 (CH_2), 28.82 (CH_2), 26.49 (CH_2), 25.51 (CH_2), 20.86 ($CH_{3\ Ac}$), 20.77 ($CH_{3\ Ac}$), 20.74 ($CH_{3\ Ac}$); HRMS (ESI) m/z calcd for $C_{21}H_{31}N_4O_{10}Cl_3Na$ $[M+Na]^+$: 627.0998; found 627.1007.

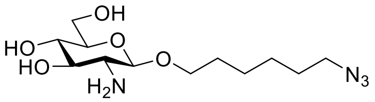

6-Azidohexyl 2-deoxy-2-amine-β-D-glucopyranoside (S2). To a solution of **S4** (73.6 mg, 0.12 mmol, 1.0 eq.) in anhydrous methanol (2.4 mL) was added sodium methoxide (1.9 mg, 0.036 mmol, 0.3 eq.). After being stirred for 1.5 h, the reaction was quenched by 1N hydrochloric acid and concentrated in *vacuo.* The residue was purified by Chromabond® C18 ec column to afford the crude product. The resulting crude was dissolved in tetrahydrofuran (1.4 mL). 1.0 M tetrabutylammonium fluoride in tetrahydrofuran solution (340 μL, 0.34 mmol) was added under an ice bath, and the reaction was allowed to warm up to room temperature, stirred for 2.5 h. The solution was concentrated then purified by Chromabond® C18 ec column to afford **S2** (5.3 mg, 14% for two steps) as the white powder. R_f = 0.51 (ethyl acetate/ methanol/ water = 7/2/1 (v/v/v)); ^{1}H NMR (500 MHz, D_2O) δ 4.75 (d, *J* = 8.5 Hz, 1 H, H-1), 4.00-3.92 (m, 2H, H-6b, OC*H*H), 3.78 (dd, *J* = 12.4, 5.3 Hz, 1 H, H-6a), 3.75-3.67 (m, 2H, OCH*H*, H-3), 3.56-3.45 (m, 2H, H-5, H-4), 3.35 (t, *J* = 6.9 Hz, 1 H, CH_2N_3), 3.04 (dd, *J* = 10.6, 8.6 Hz, 1 H, H-2), 1.73-1.60 (m, 4 H, 2×CH_2), 1.47-1.35 (m, 4 H, 2×CH_2); ^{13}C NMR (125 MHz, D_2O) δ 100.43 (C-1), 77.86 (C-5), 73.72 (C-3), 72.33 (OCH_2), 71.48 (C-4), 62.11 (C-6), 57.45 (C-2), 52.81 (CH_2N_3), 30.20 (CH_2), 29.53 (CH_2), 27.33 (CH_2), 26.26 (CH_2); HRMS (ESI) m/z calcd for $C_{14}H_{24}N_4O_5Na$ $[M+Na]^+$: 327.1644; found 327.1644.

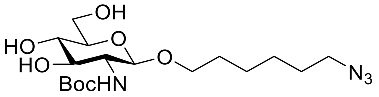

6-Azidohexyl 2-deoxy-2-(*t*-butyloxycarbonylamino)-β-D-glucopyranoside (S3). To a solution of **S4** (66.1 mg, 0.11 mmol, 1.0 eq.) in anhydrous methanol (2.2 mL) was added sodium methoxide (1.8 mg, 0.033 mmol, 0.3 eq.). After being stirred for 1 h, the reaction was quenched by 1N hydrochloric acid and concentrated in *vacuo.* The residue was purified by Chromabond® C18 ec column to afford the

crude product. The resulting crude was dissolved in tetrahydrofuran (1.8 mL). 1.0 M tetrabutylammonium fluoride in tetrahydrofuran solution (440 μL, 0.44 mmol, 4 eq.) was added under an ice bath, and the reaction was allowed to warm up to room temperature, stirred for 23 h. The solution was concentrated in *vacuo* to afford the crude compound. The resulting crude was added methanol (2.2 mL) and stirred in an ice bath for 5 min, followed by addition of triethylamine (15 μL, 0.11 mmol). Di-*tert*-butyldicarbonate (39 μL, 0.17 mmol, 1.5 eq.) was added dropwise, and the solution was stirred at room temperature for 5.5 h. Then, the solution was concentrated in *vacuo* and purified by Chromabond® C18 ec column (methanol/ water = 11/2 (v/v)) to afford the **S3** (12 mg, 27% for three steps) as the white powder. R_f = 0.77 (ethyl acetate/ methanol/ water = 7/2/1 (v/v/v)); ^{1}H NMR (400 MHz, D_2O/ DMSO-d_6 = 9/1 (v/v)) δ 4.46 (d, *J* = 8.5 Hz, 1H, H-1), 3.99-3.87 (m, 2H, H-6b, OC*H*H), 3.78-3.69 (m, 1 H, H-6a), 3.66-3.56 (m, 1H, OCH*H*), 3.54-3.46 (m, 1H, H-5), 3.45-3.38 (m, 2H, H-4, H-3), 3.38-3.27 (m, 2H, CH_2N_3, H-2), 1.69-1.53 (m, 4 H, 2×CH_2), 1.53-1.44 (m, 9H, 3×$CH_{3\,Boc}$), 1.44-1.34 (m, 4 H, 2×CH_2); ^{13}C NMR (100 MHz, D_2O/ DMSO-d_6 = 9/1 (v/v)) δ 159.28 (C_{Boc}), 103.13 (C-1), 82.16 ($C(CH_3)_3$), 77.37 (C-5), 75.33 (C-3), 71.67 (C-4, OCH_2), 62.38 (C-6), 58.41 (C-2), 52.67 (CH_2N_3), 30.19 (CH_2), 29.60 (CH_2), 29.41 (3×$CH_{3\,Boc}$), 27.30 (CH_2), 26.30 (CH_2); HRMS (ESI) m/z calcd for $C_{17}H_{32}N_4O_7Na$ $[M+Na]^+$: 427.2168; found 427.2170.

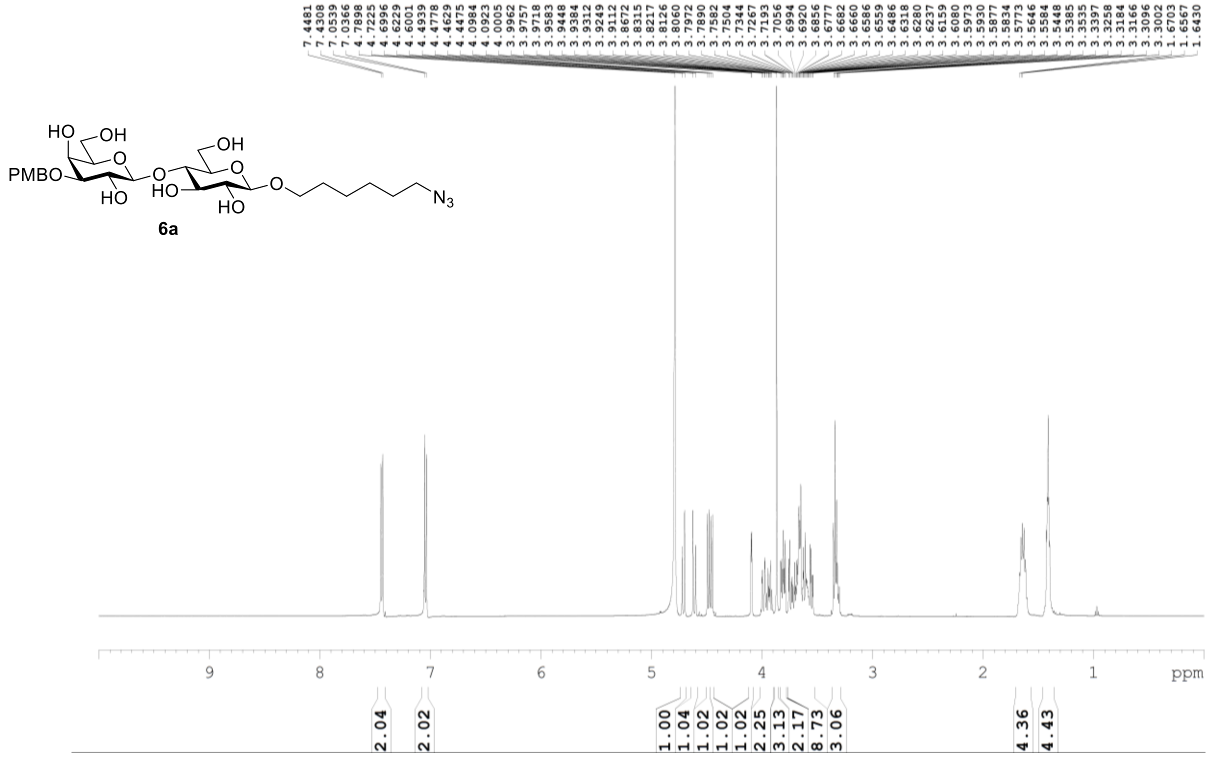

^{1}H NMR spectrum of **6a**, recorded at 500 MHz in D_2O. Residual water signal was used as reference (δ = 4.79 ppm).

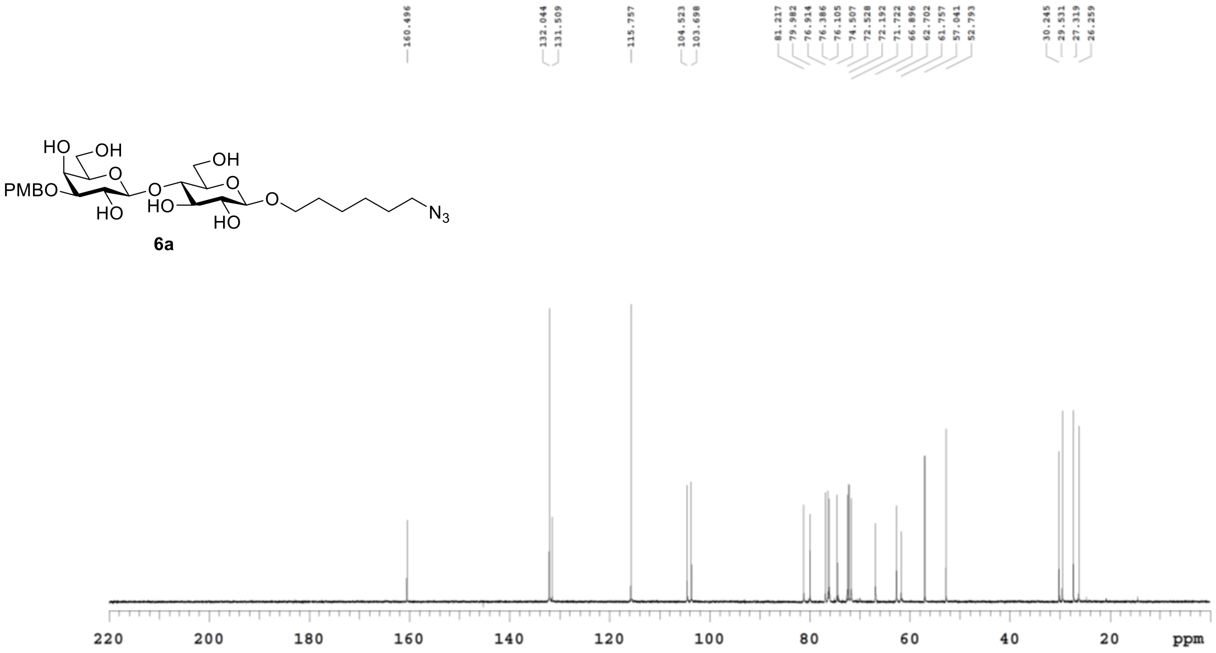

^{13}C NMR spectrum of **6a**, recorded at 125 MHz in D_2O.

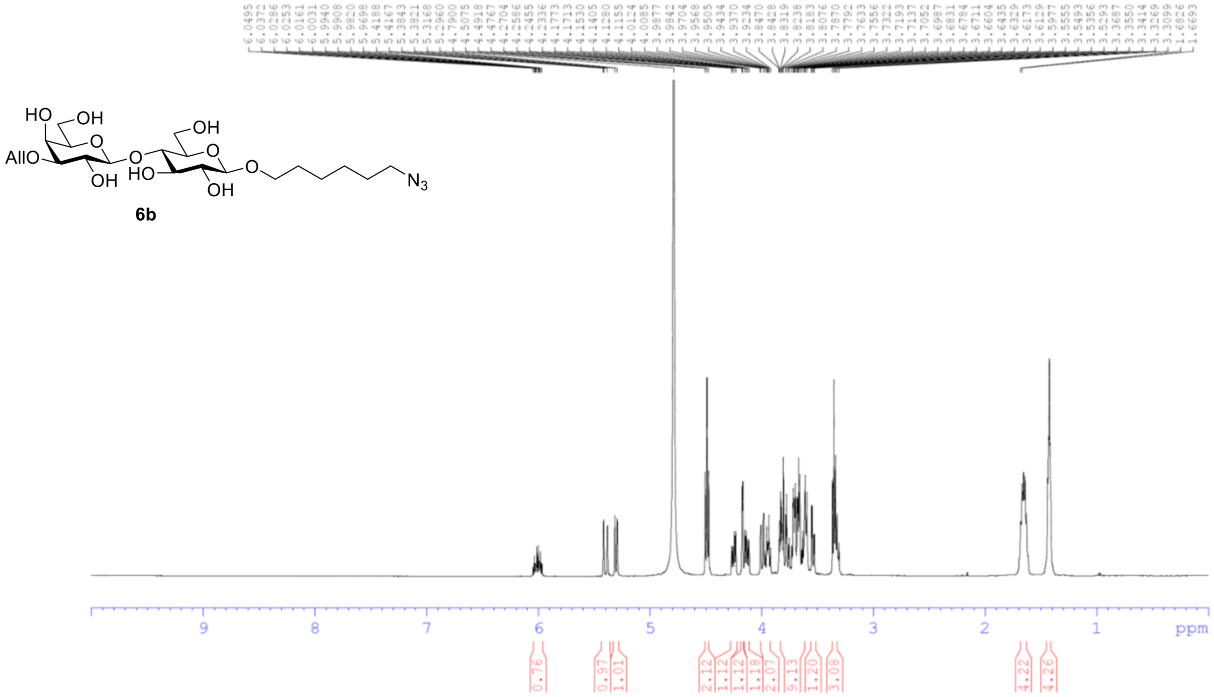

^{1}H NMR spectrum of **6b**, recorded at 500 MHz in D_2O. Residual water signal was used as reference (δ = 4.79 ppm).

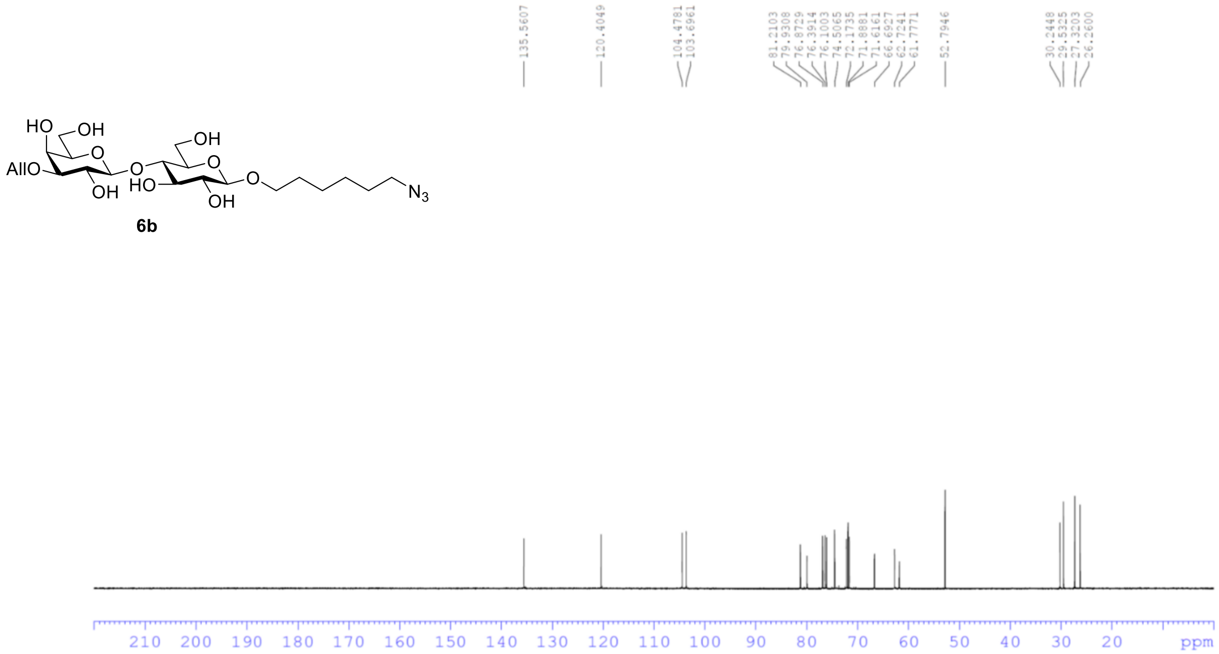

^{13}C NMR spectrum of **6b**, recorded at 125 MHz in D_2O.

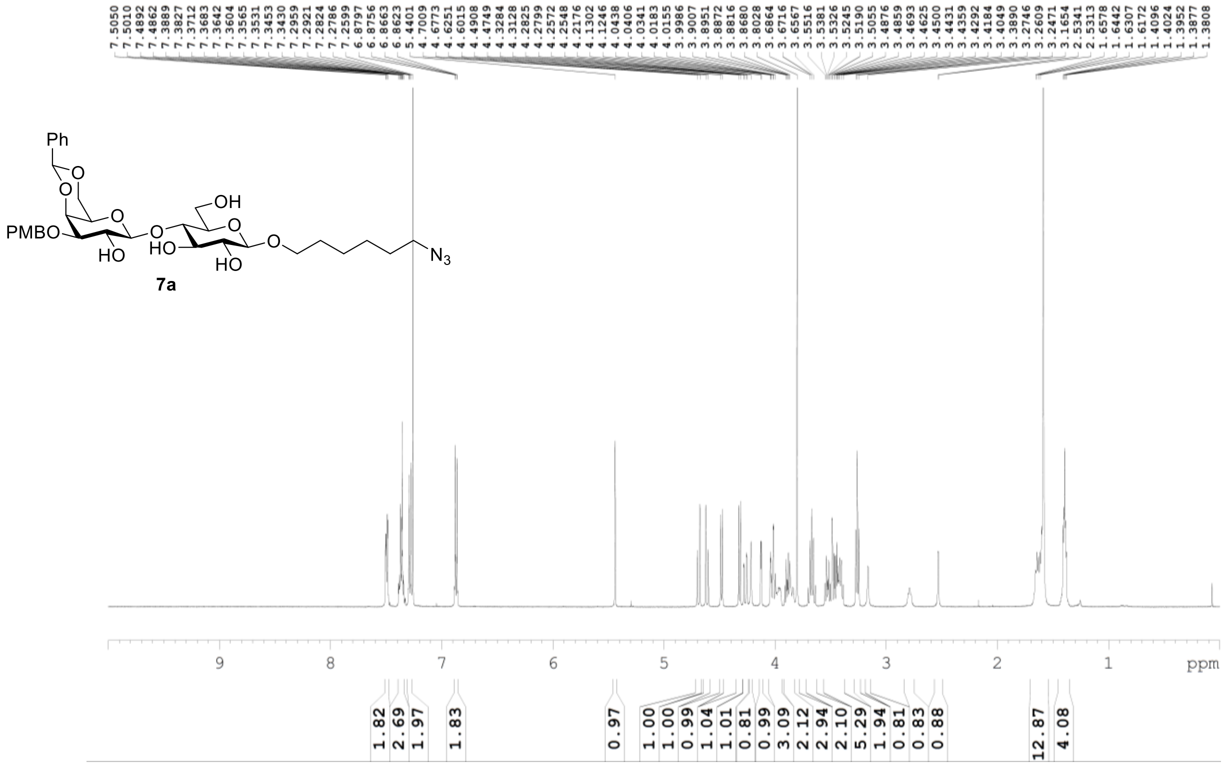

^{1}H NMR spectrum of **7a**, recorded at 500 MHz in $CDCl_3$ (δ = 7.26 ppm).

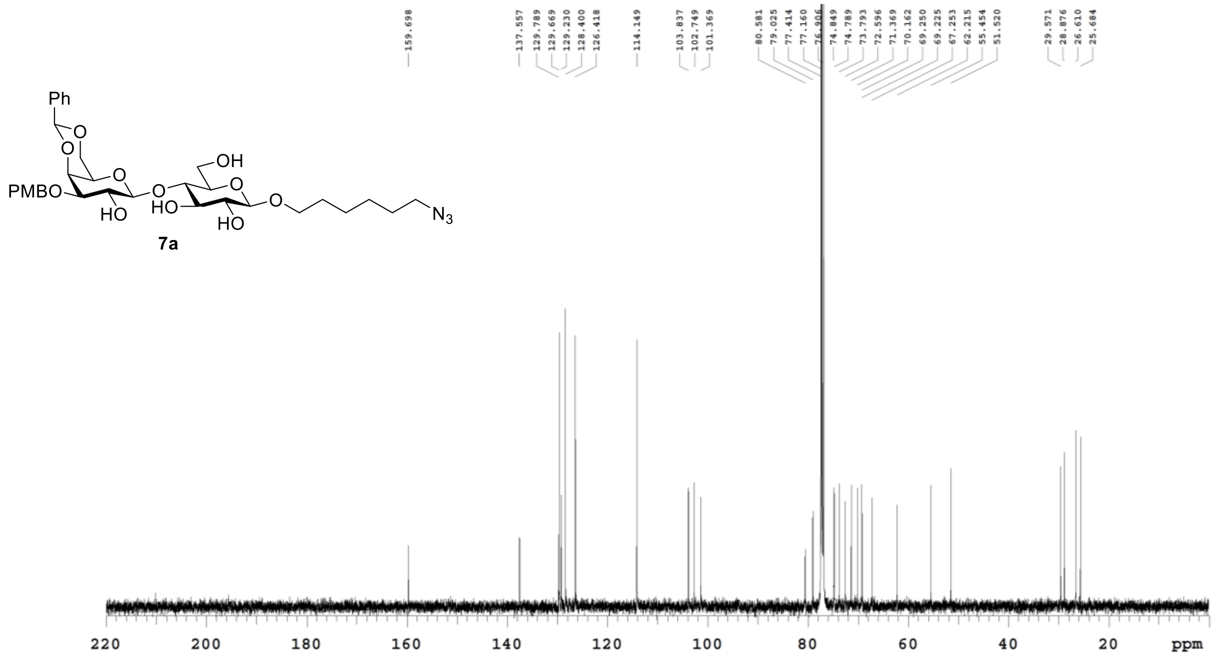

^{13}C NMR spectrum of **7a**, recorded at 125 MHz in $CDCl_3$ (δ = 77.16 ppm).

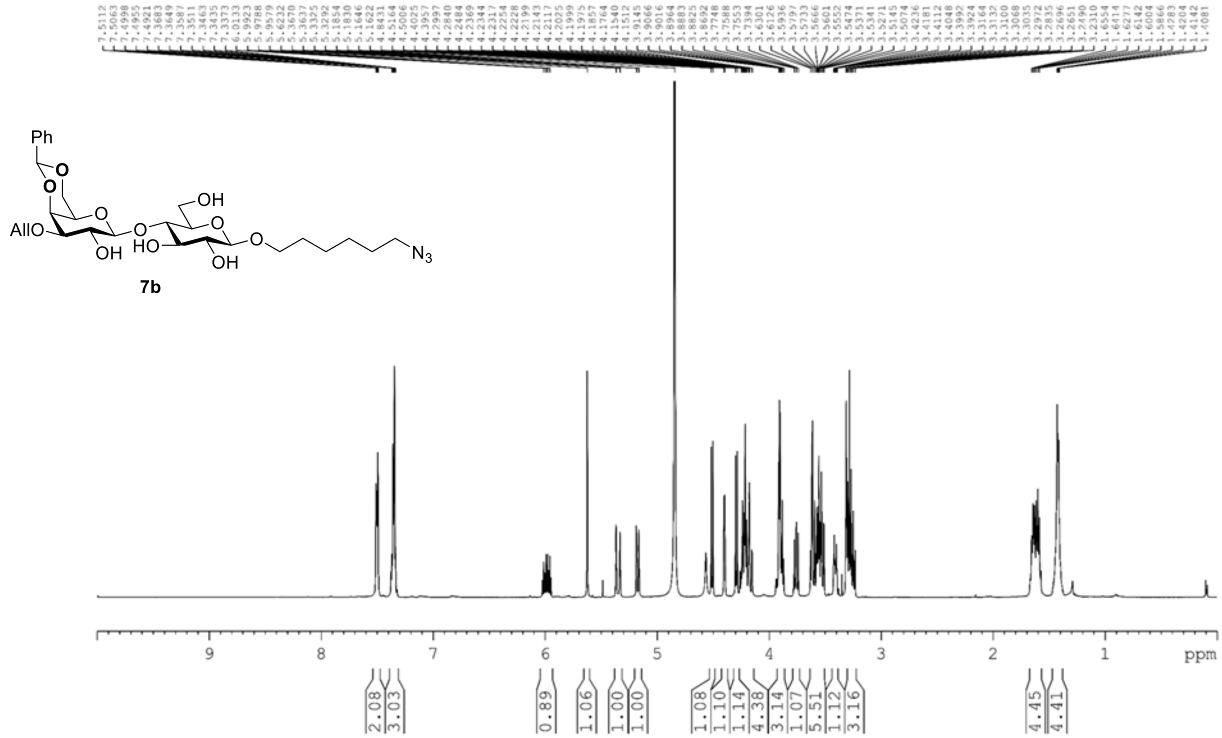

^{1}H NMR spectrum of **7b**, recorded at 500 MHz in CD_3OD (δ = 3.31 ppm).

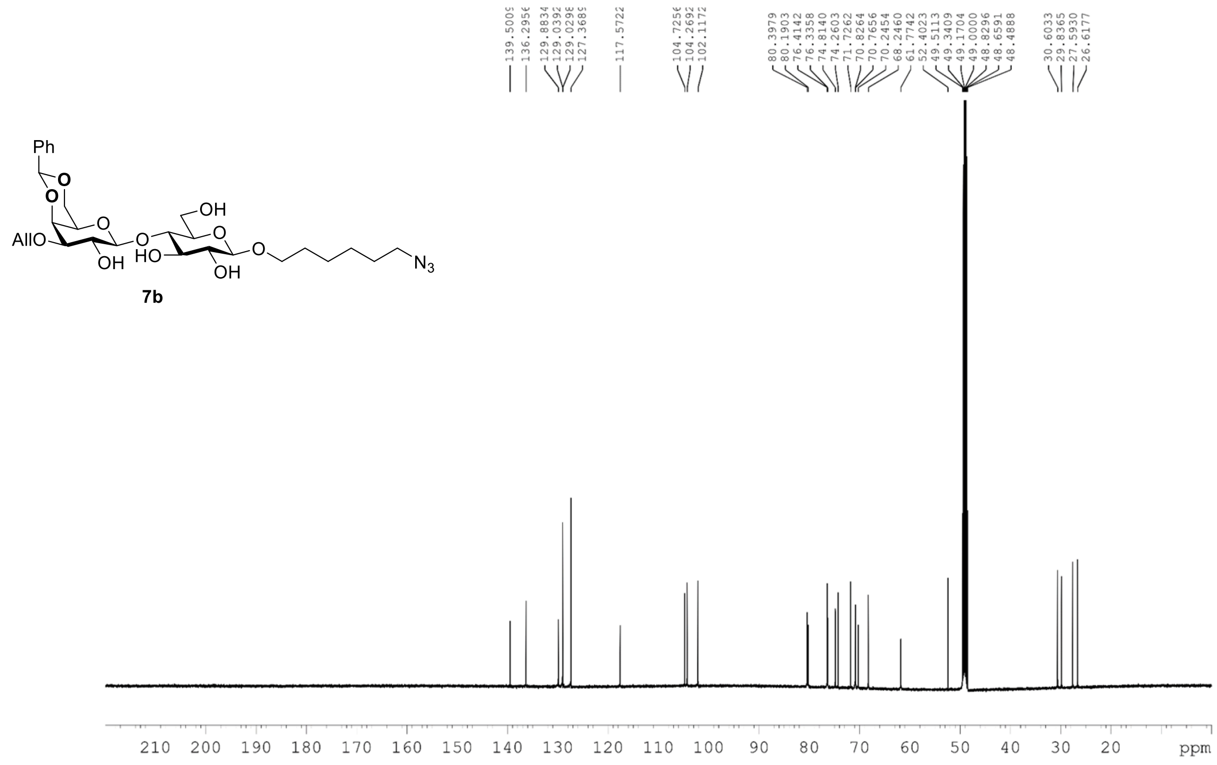

^{13}C NMR spectrum of **7b**, recorded at 125 MHz in CD_3OD (δ = 49.00 ppm).

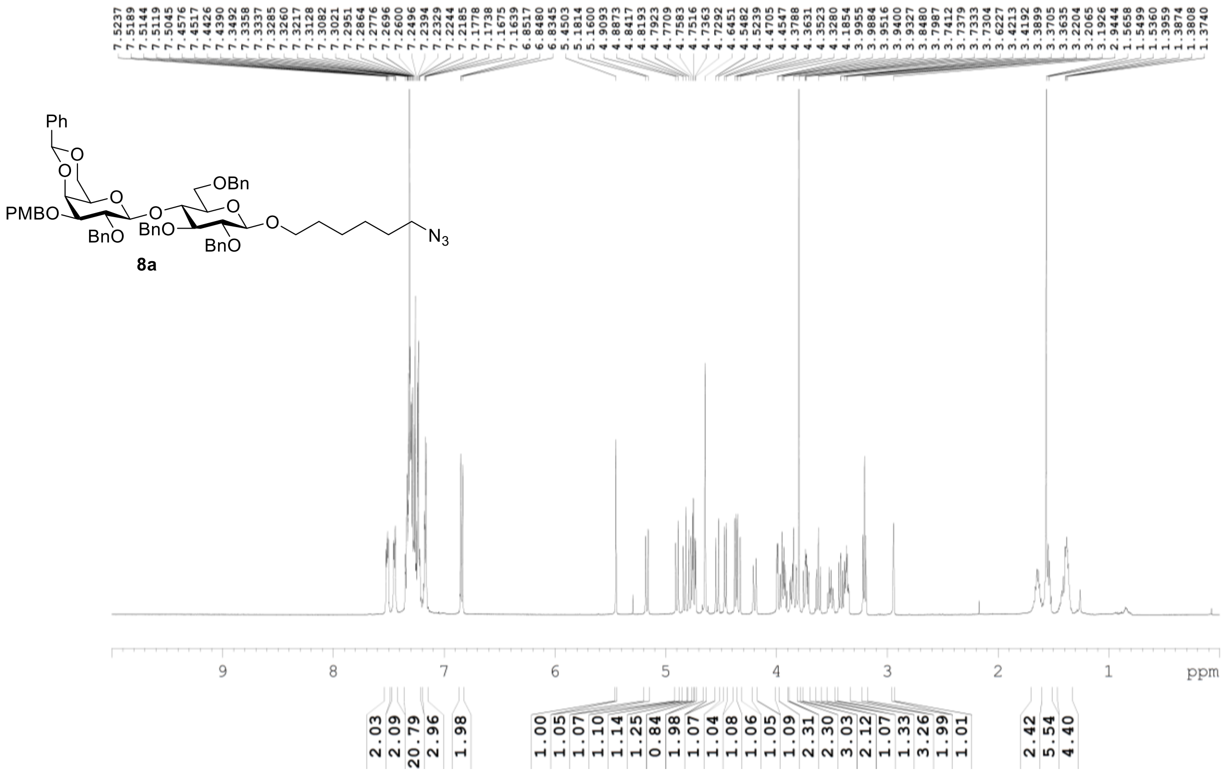

^{1}H NMR spectrum of **8a**, recorded at 500 MHz in $CDCl_3$ (δ = 7.26 ppm).

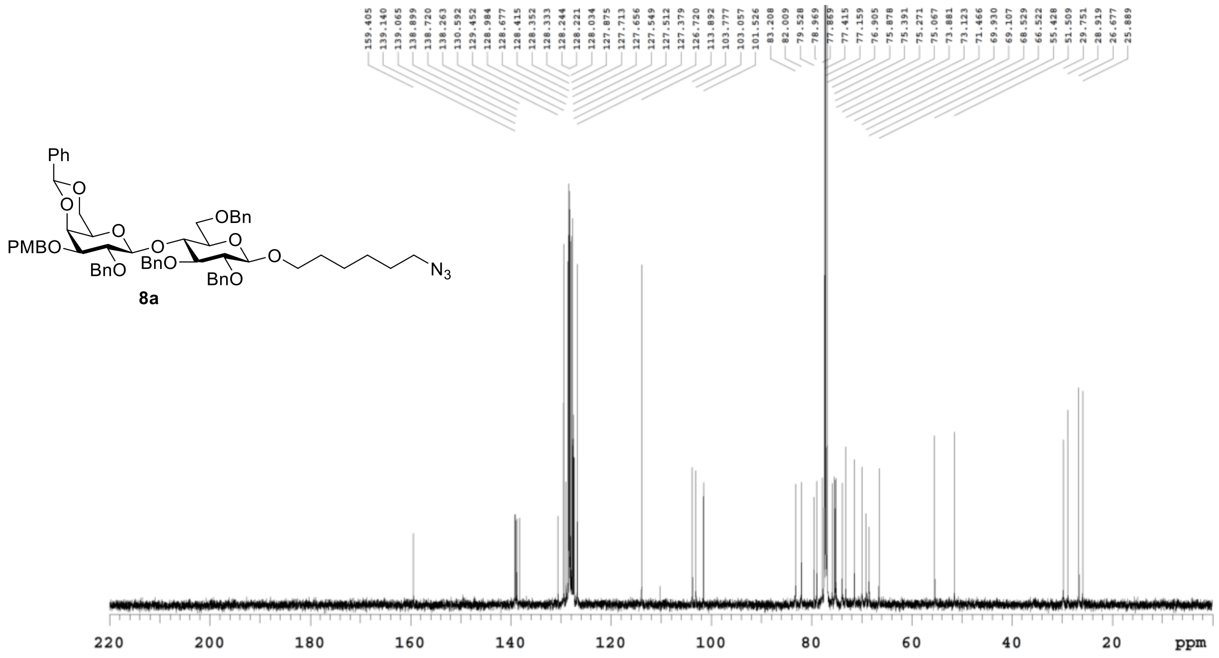

^{13}C NMR spectrum of **8a**, recorded at 125 MHz in $CDCl_3$ (δ = 77.16 ppm).

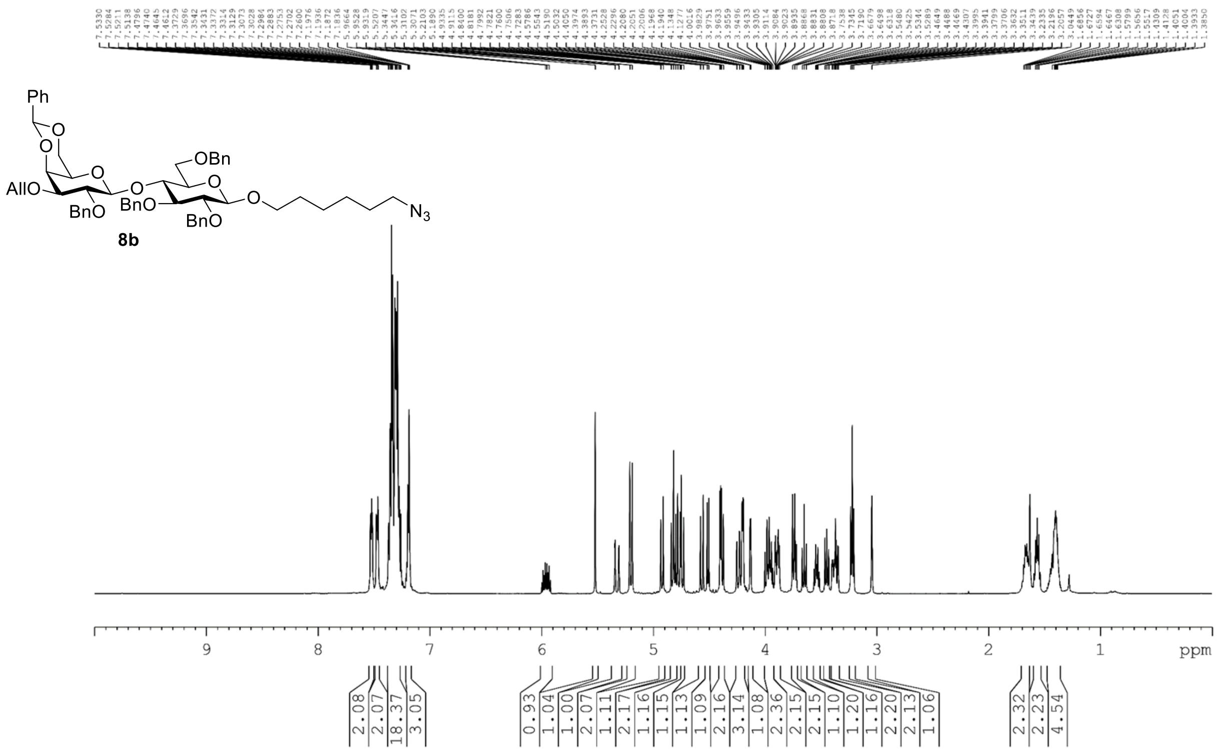

^{1}H NMR spectrum of **8b**, recorded at 500 MHz in $CDCl_3$ (δ = 7.26 ppm).

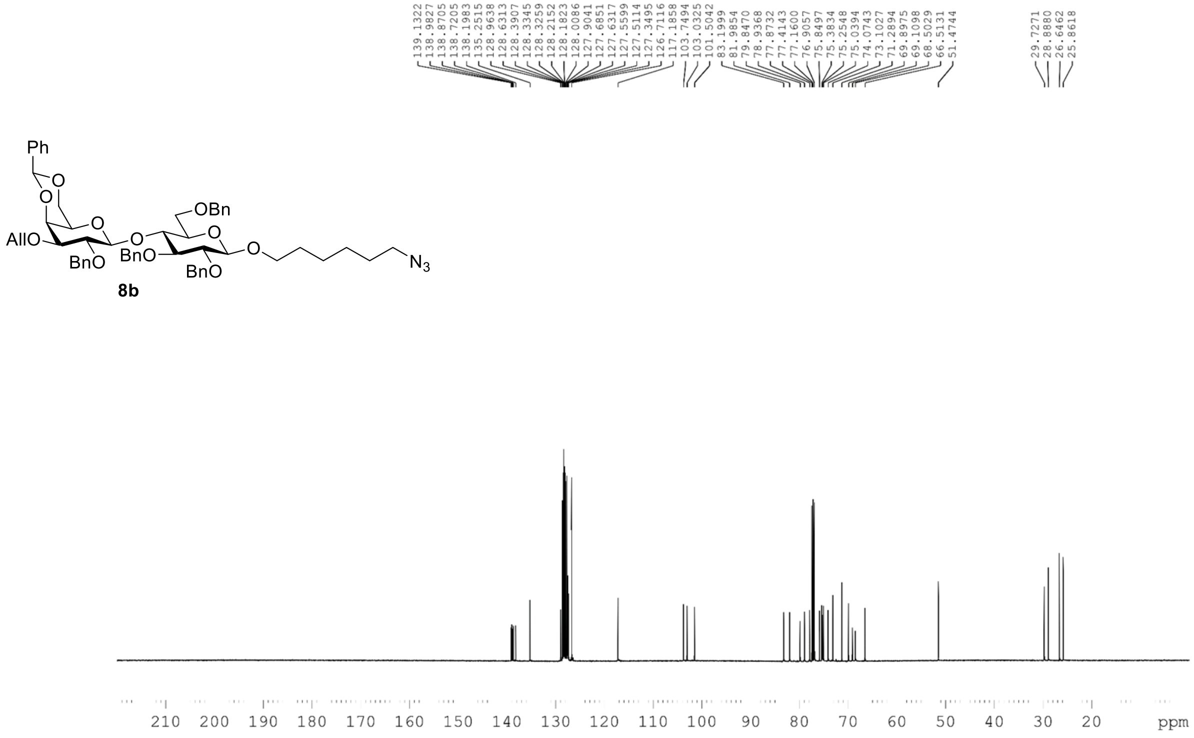

^{13}C NMR spectrum of **8b**, recorded at 125 MHz in $CDCl_3$ (δ = 77.16 ppm).

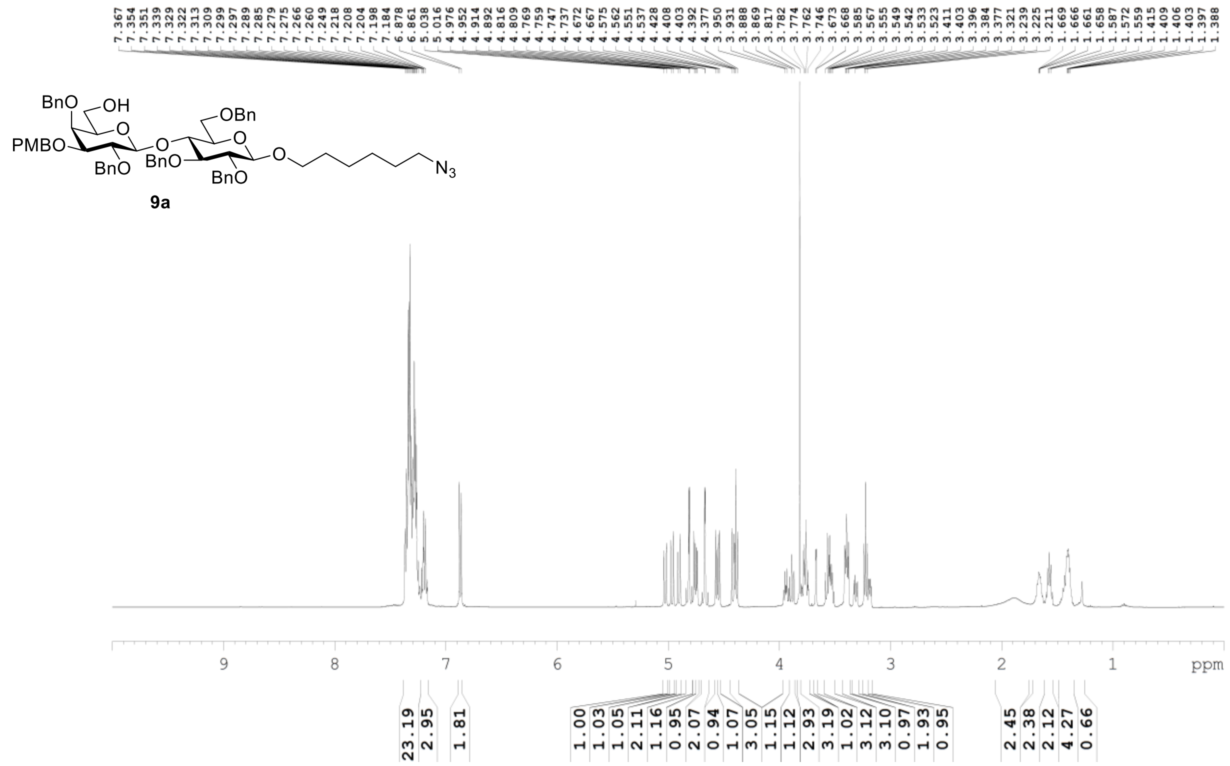

^{1}H NMR spectrum of **9a**, recorded at 500 MHz in $CDCl_3$ (δ = 7.26 ppm).

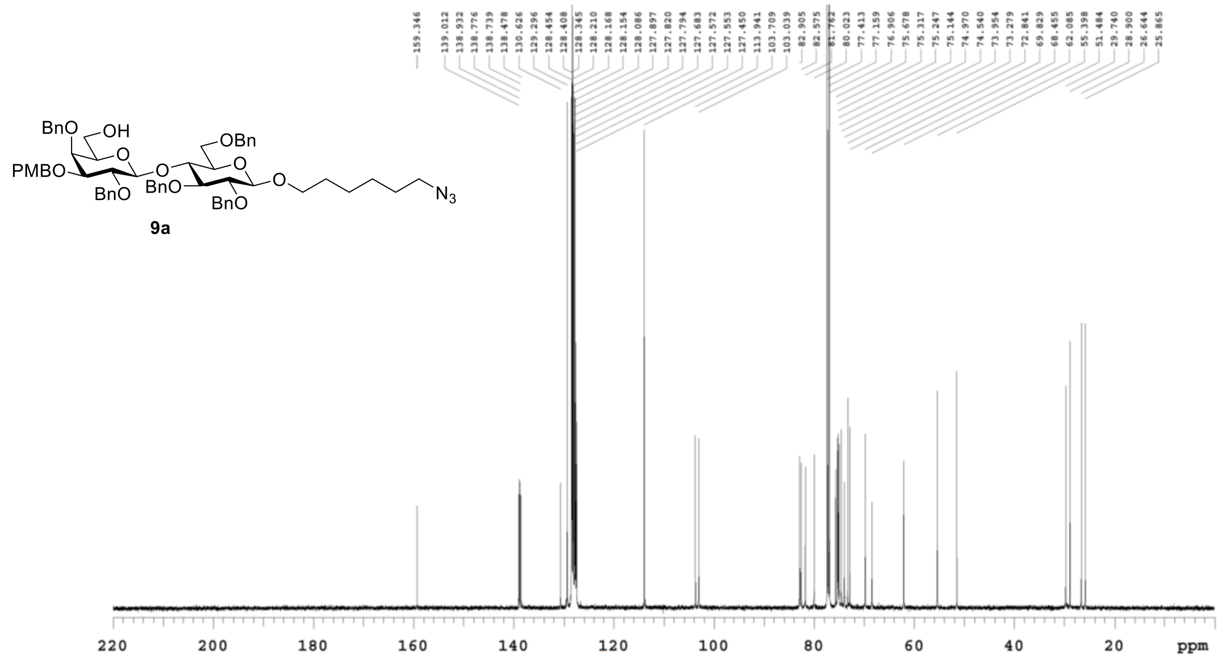

^{13}C NMR spectrum of **9a**, recorded at 125 MHz in $CDCl_3$ (δ = 77.16 ppm).

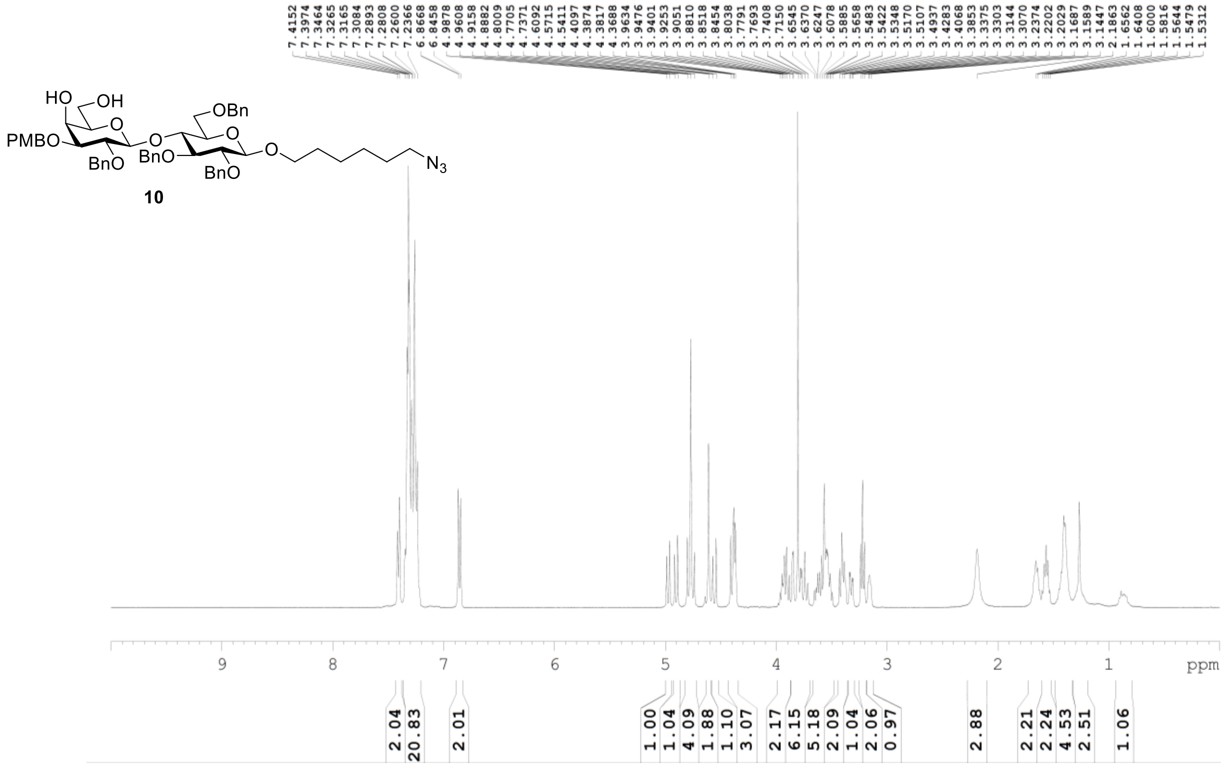

^{1}H NMR spectrum of **10**, recorded at 400 MHz in $CDCl_3$ (δ = 7.26 ppm).

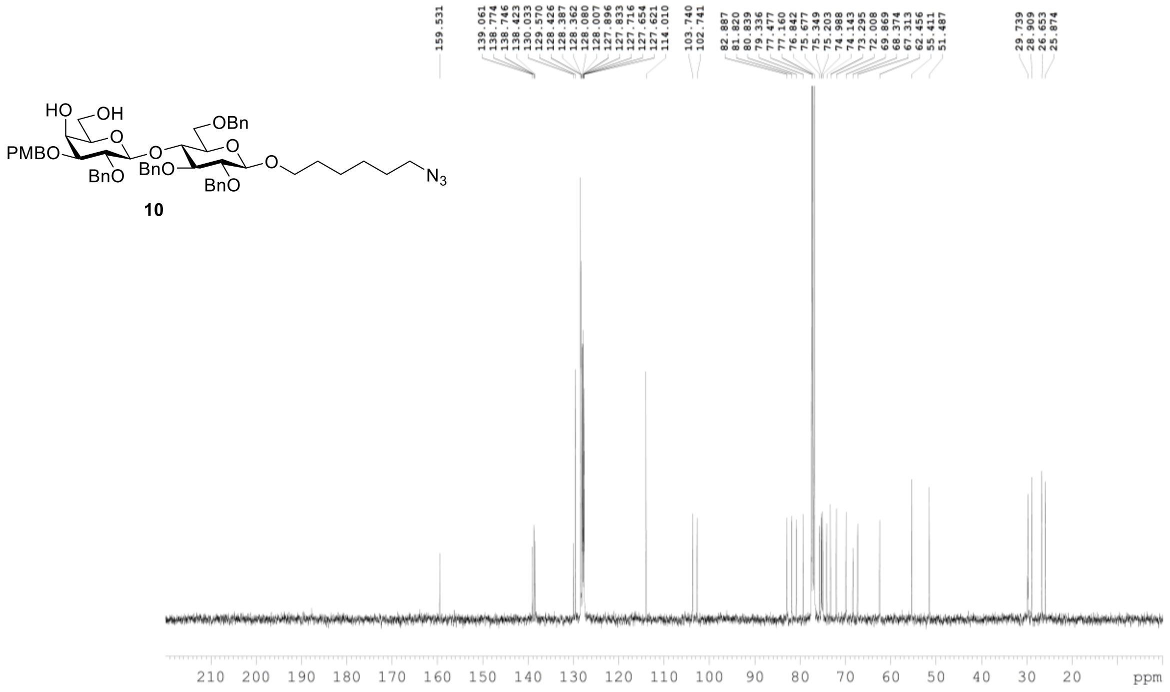

^{13}C NMR spectrum of **10**, recorded at 100 MHz in $CDCl_3$ (δ = 77.16 ppm).

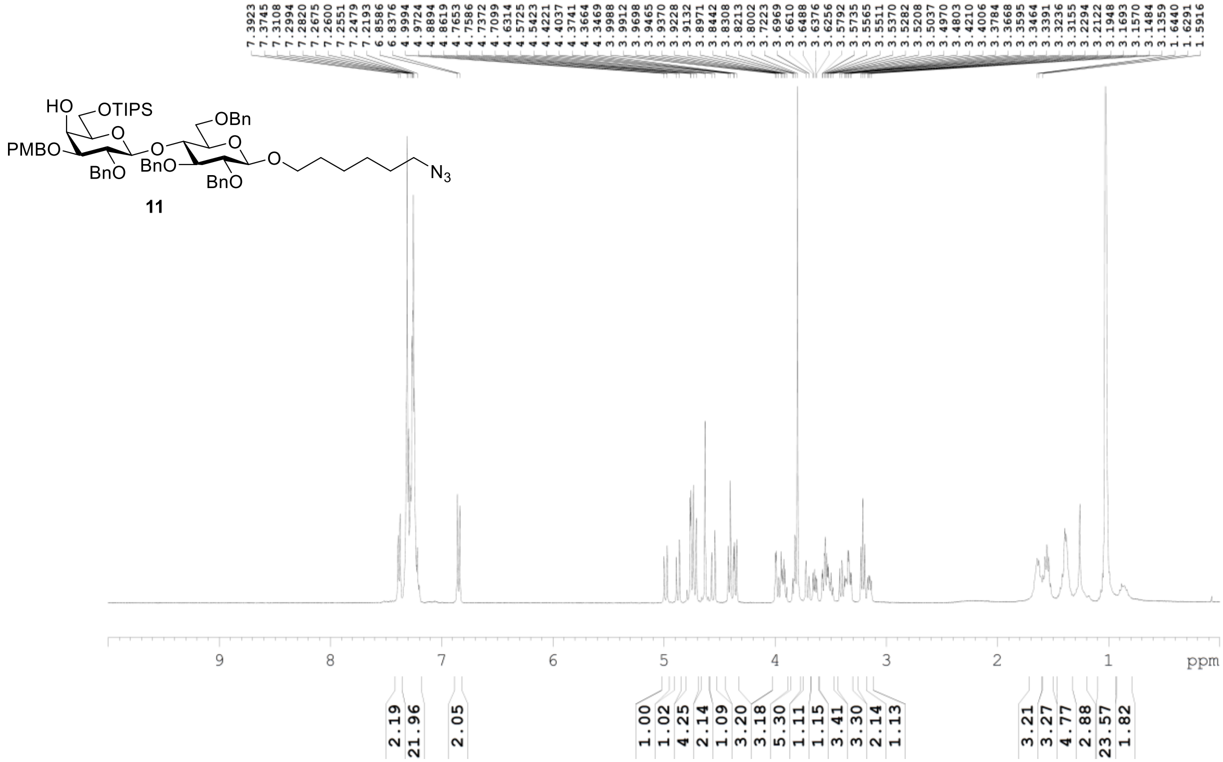

^{1}H NMR spectrum of **11**, recorded at 400 MHz in $CDCl_3$ (δ = 7.26 ppm).

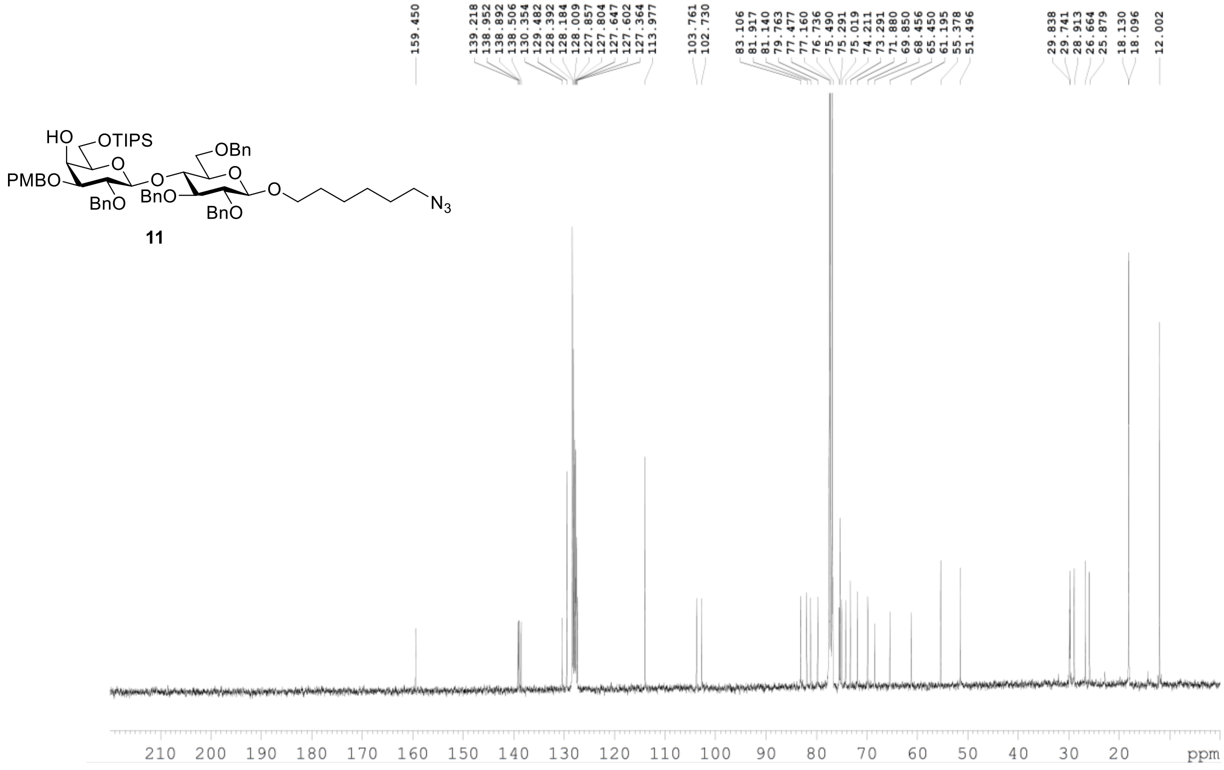

^{13}C NMR spectrum of **11**, recorded at 100 MHz in $CDCl_3$ (δ = 77.16 ppm).

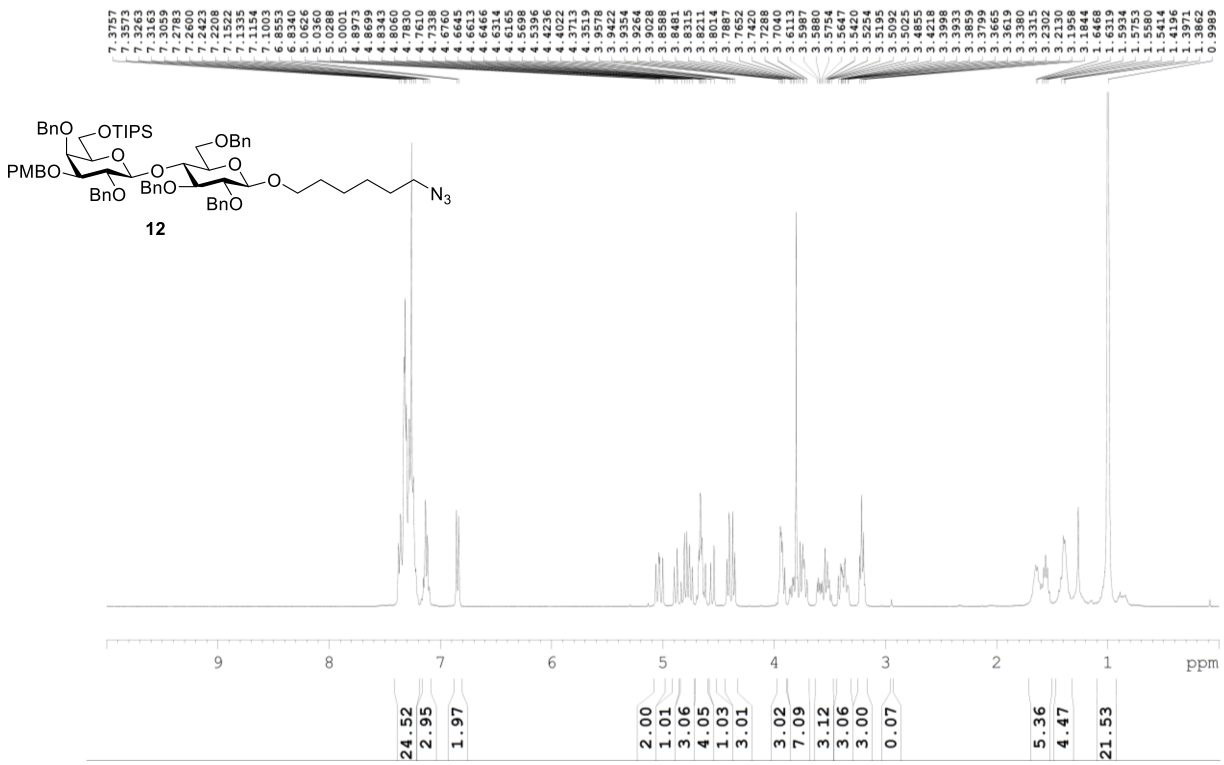

^{1}H NMR spectrum of **12**, recorded at 400 MHz in $CDCl_3$ (δ = 7.26 ppm).

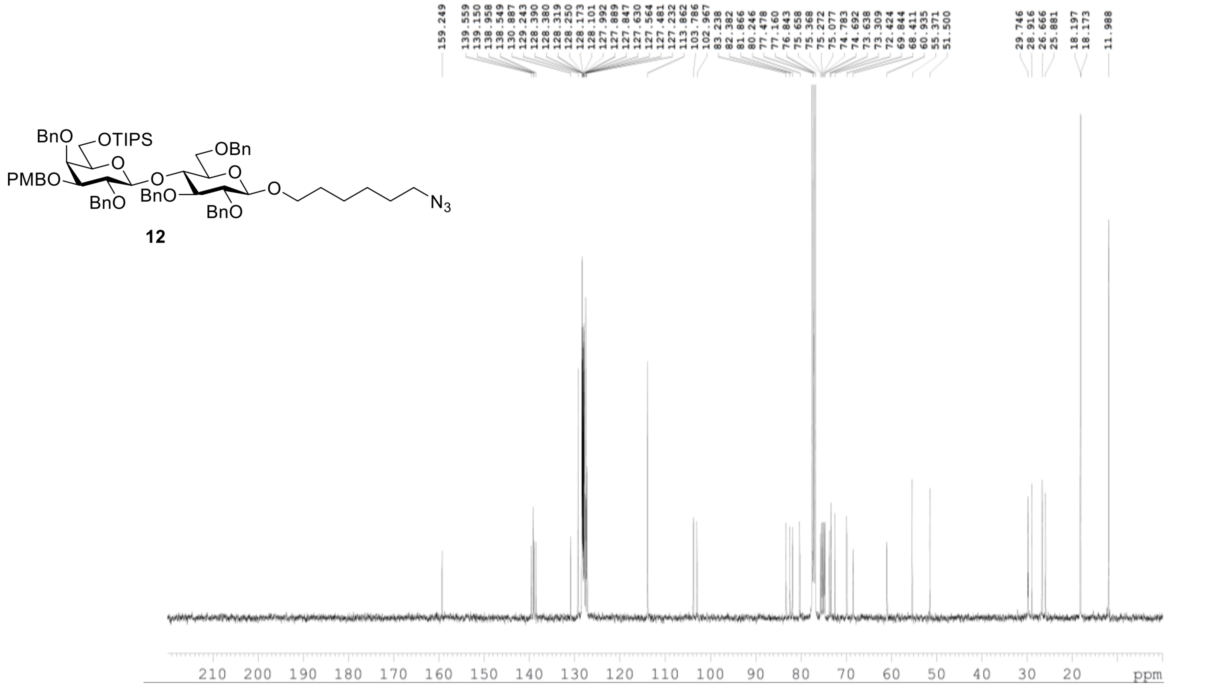

^{13}C NMR spectrum of **12**, recorded at 100 MHz in $CDCl_3$ (δ = 77.16 ppm).

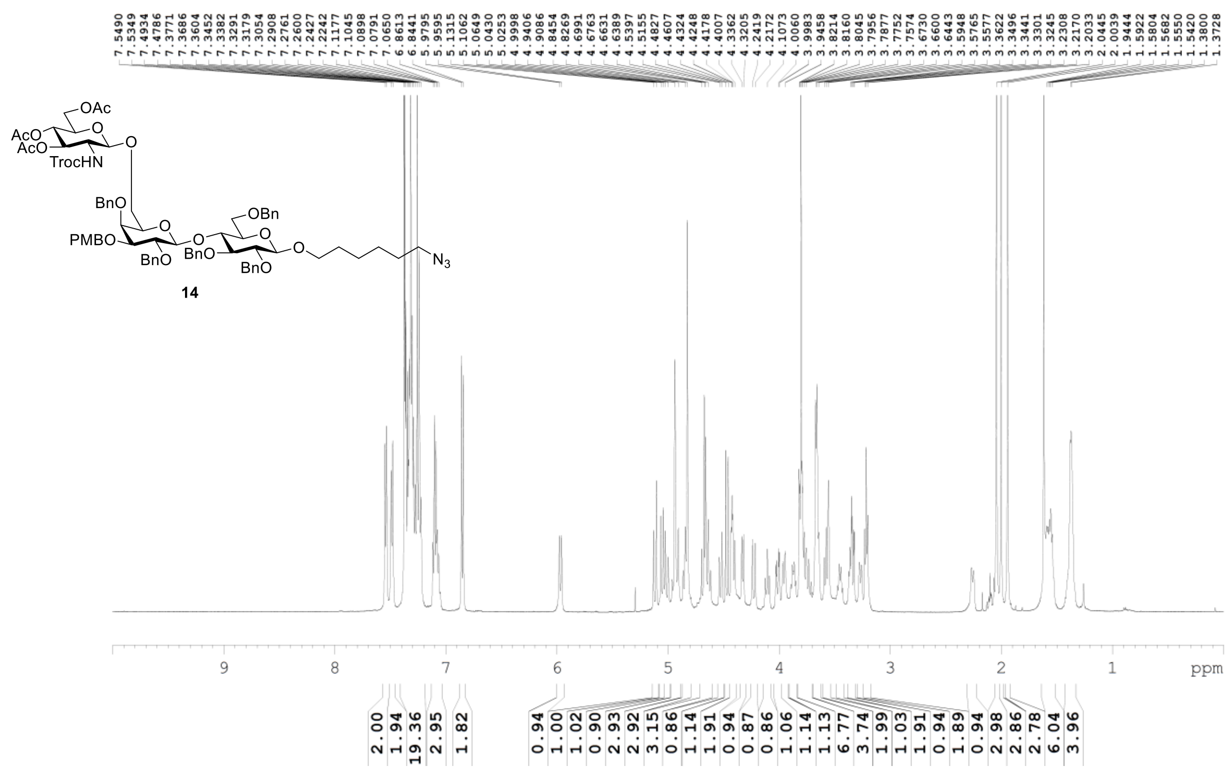

^{1}H NMR spectrum of **14**, recorded at 500 MHz in $CDCl_3$ (δ = 7.26 ppm).

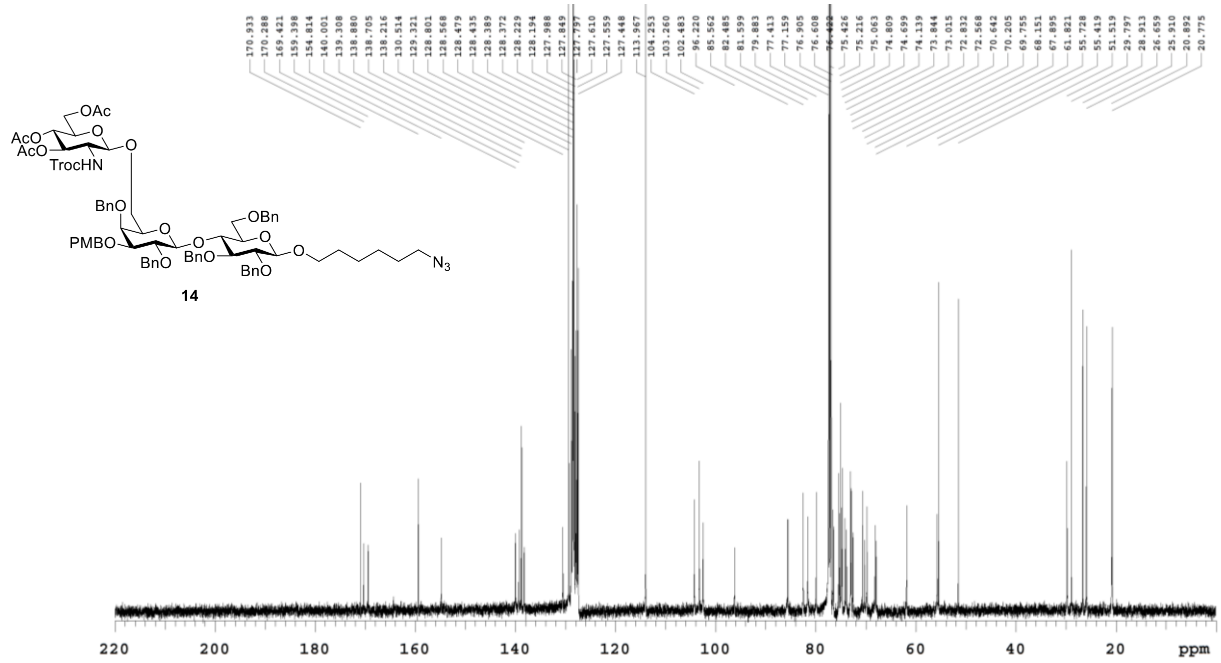

^{13}C NMR spectrum of **14**, recorded at 125 MHz in $CDCl_3$ (δ = 77.16 ppm).

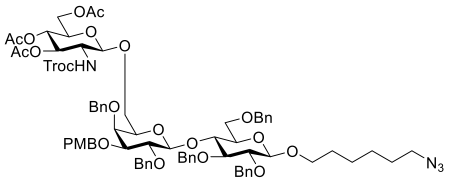

14

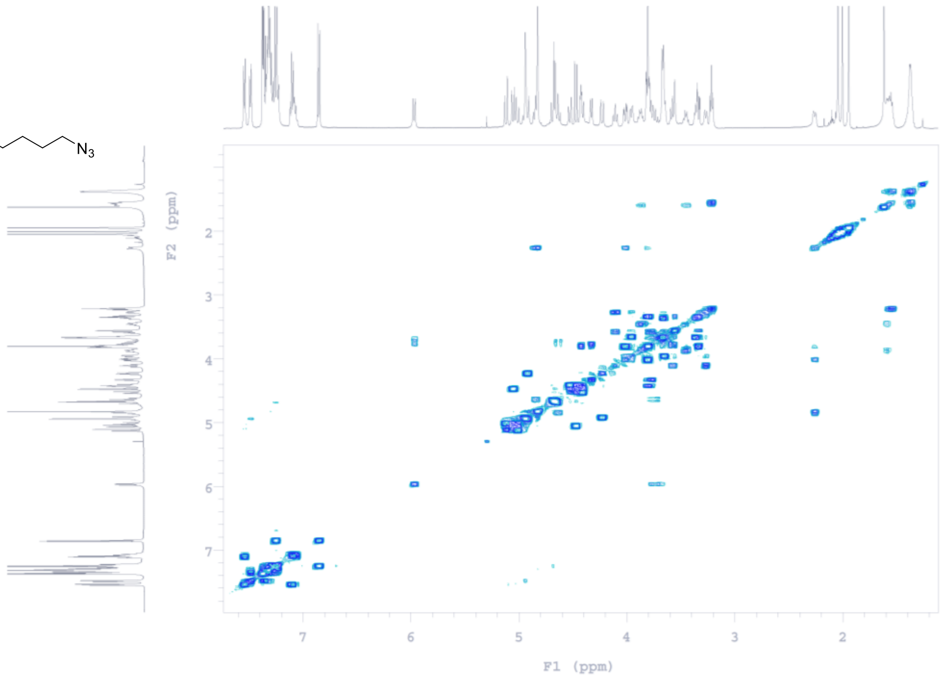

COSY NMR spectrum of **14**, recorded at 500/125 MHz in $CDCl_3$.

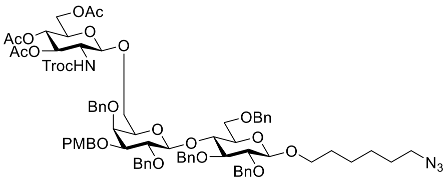

14

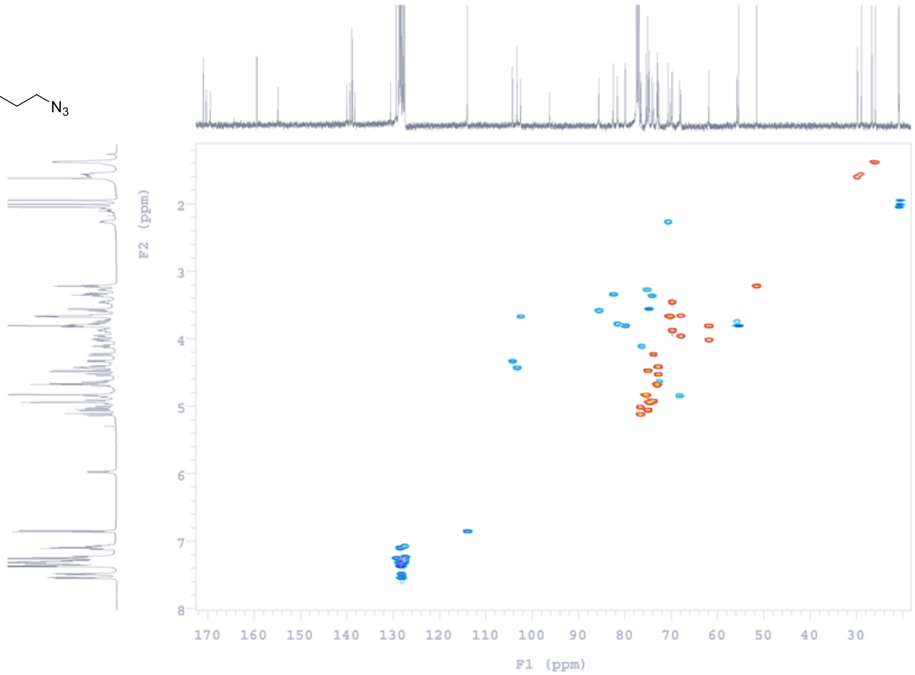

HSQC NMR spectrum of **14**, recorded at 500/125 MHz in $CDCl_3$.

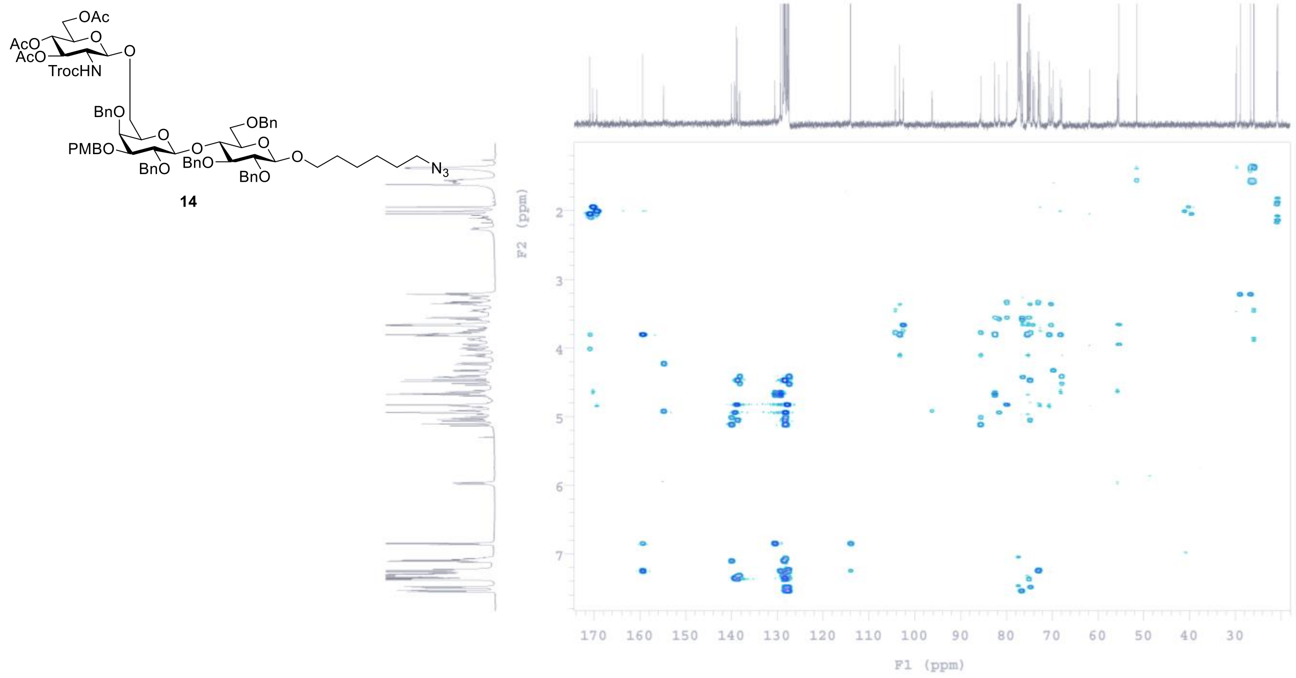

HMBC NMR spectrum of **14**, recorded at 500/125 MHz in $CDCl_3$.

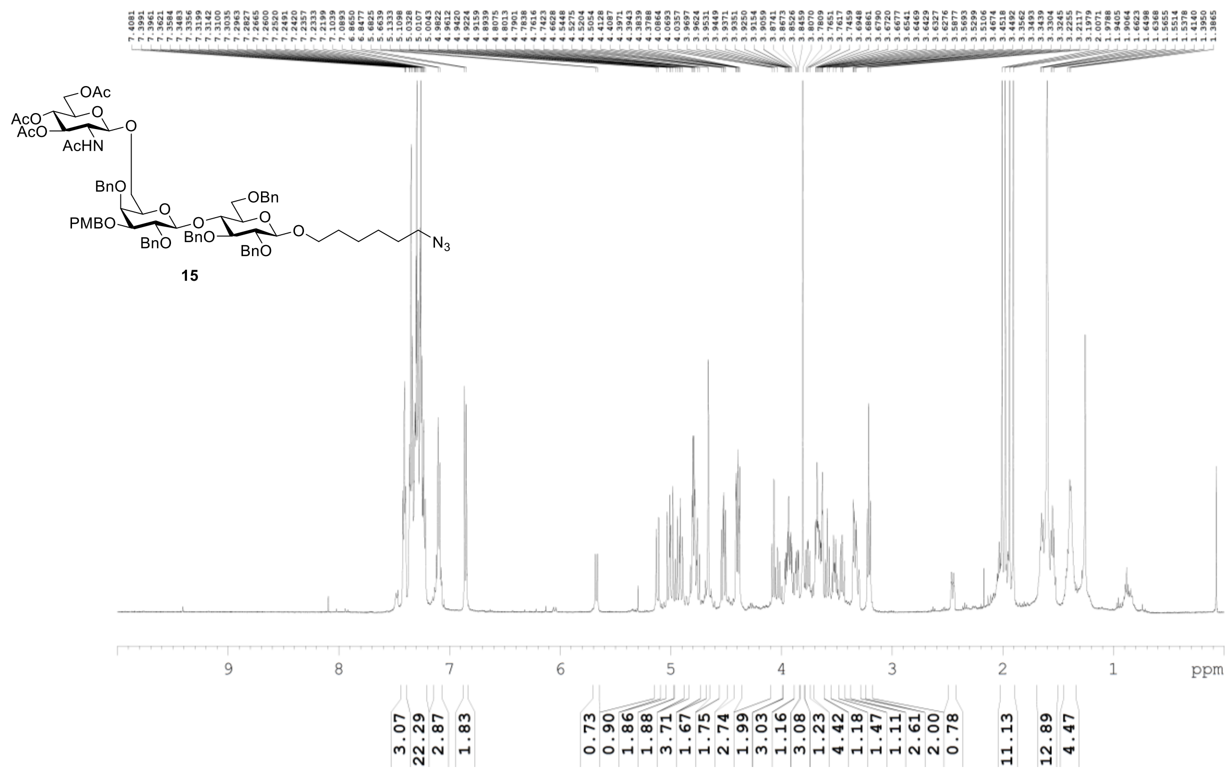

^{1}H NMR spectrum of **15**, recorded at 500 MHz in $CDCl_3$ ($\delta = 7.26$ ppm).

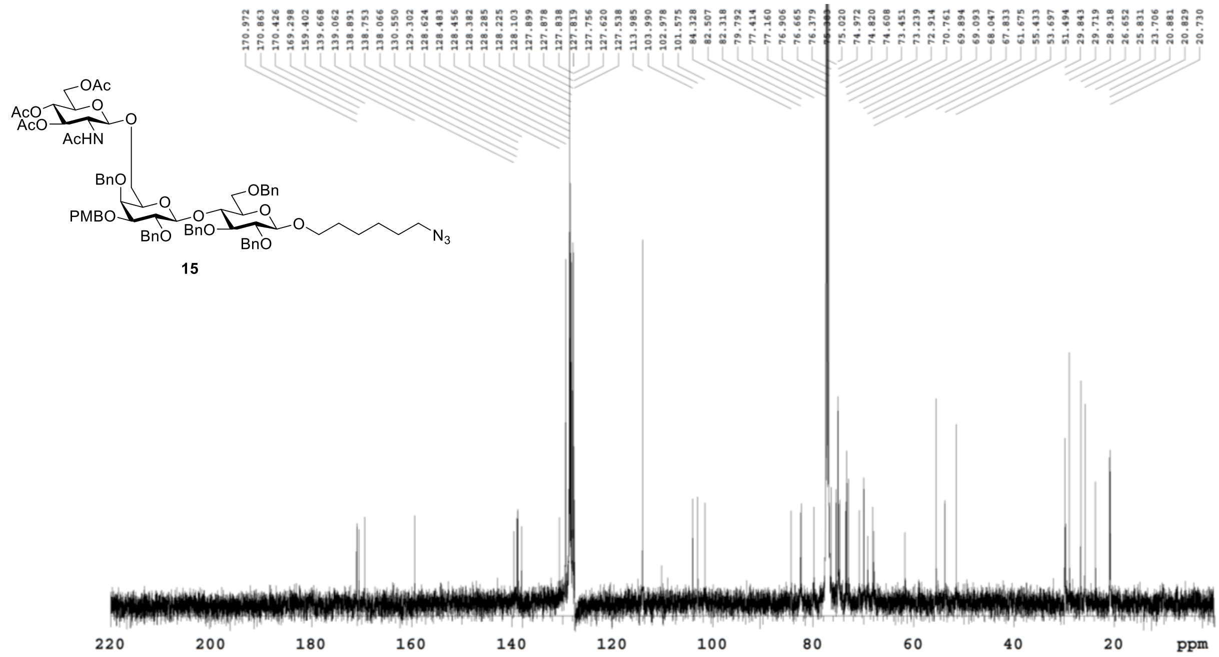

^{13}C NMR spectrum of **15**, recorded at 125 MHz in $CDCl_3$ ($\delta = 77.16$ ppm).

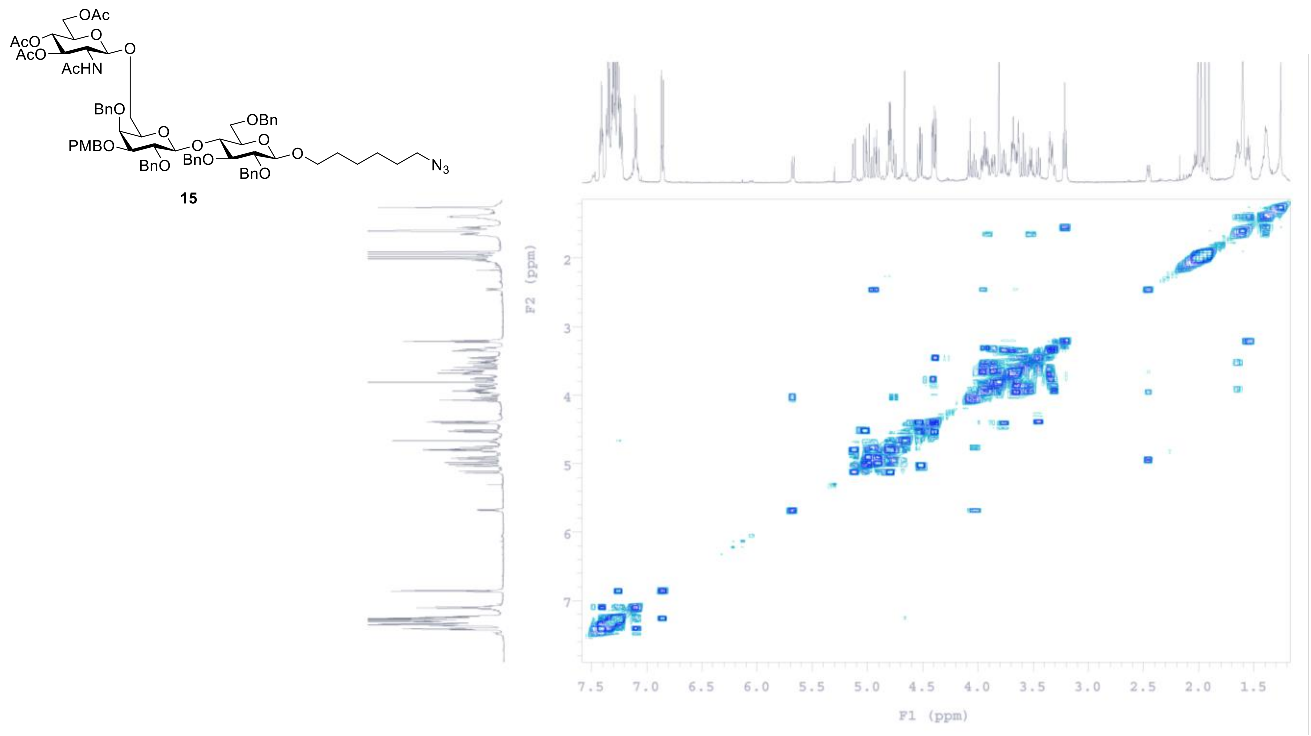

COSY NMR spectrum of **15**, recorded at 500/125 MHz in $CDCl_3$.

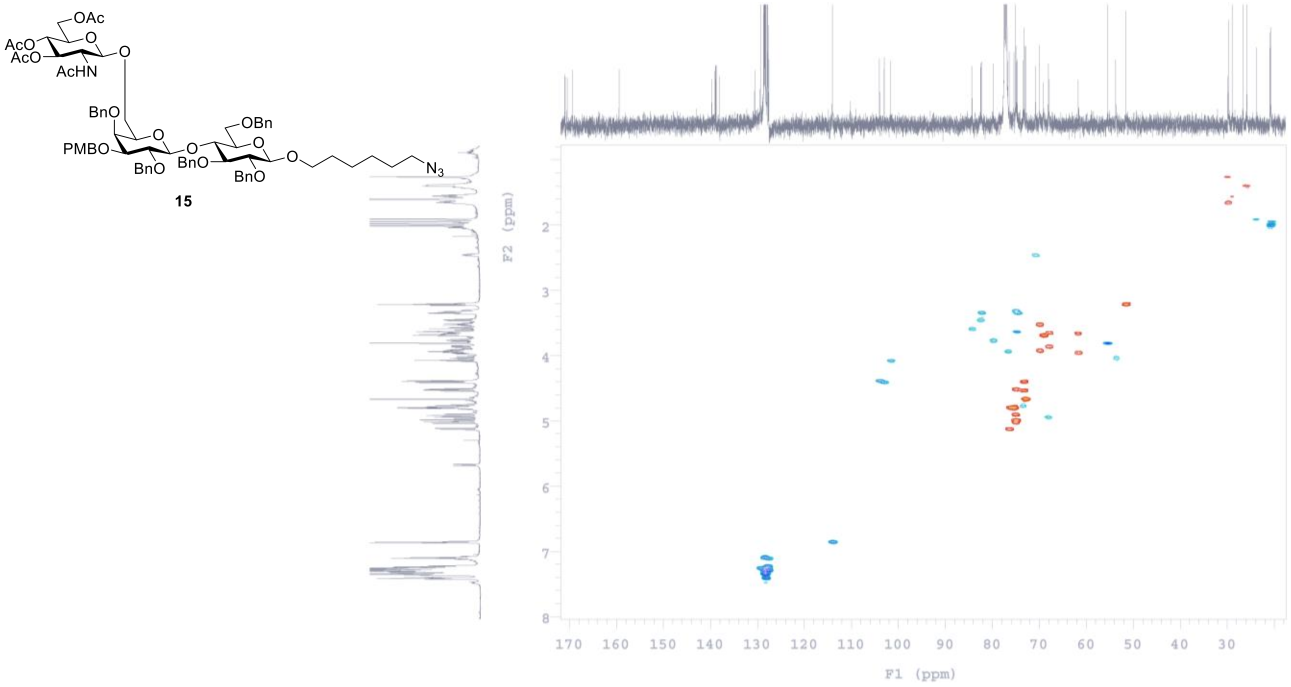

HSQC NMR spectrum of **15**, recorded at 500/125 MHz in $CDCl_3$.

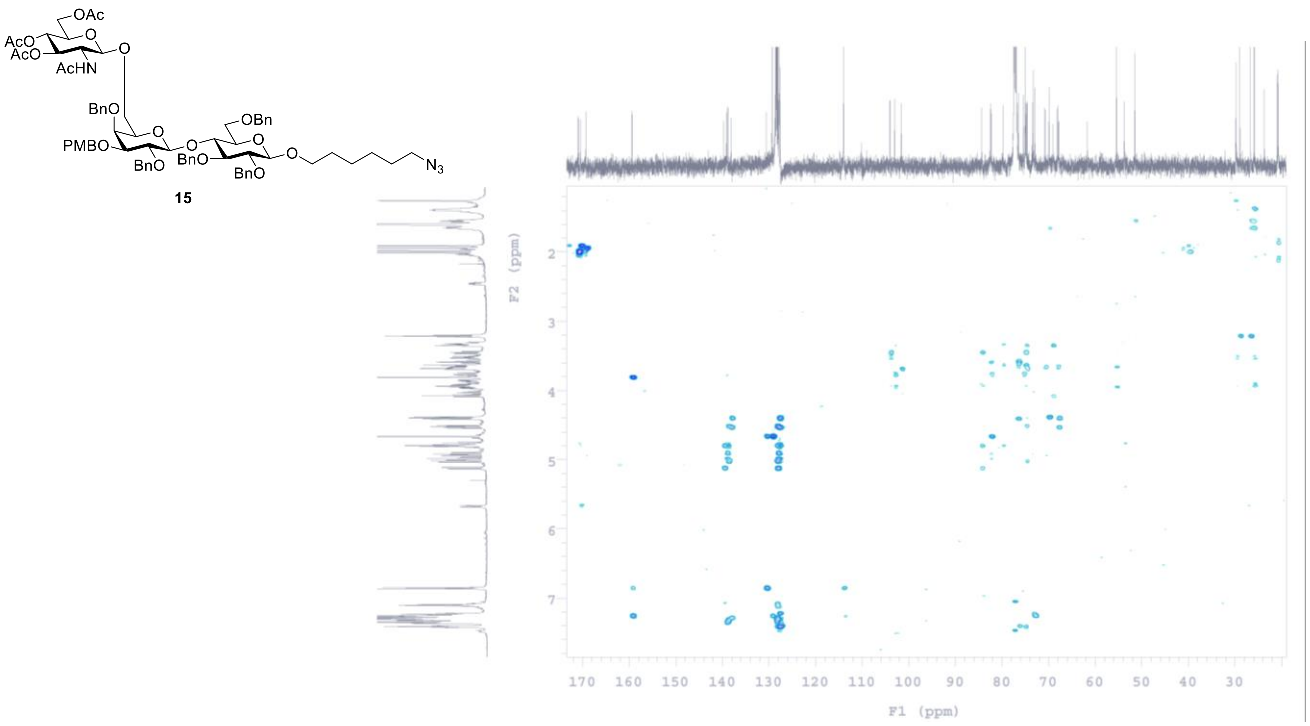

HMBC NMR spectrum of **15**, recorded at 500/125 MHz in $CDCl_3$.

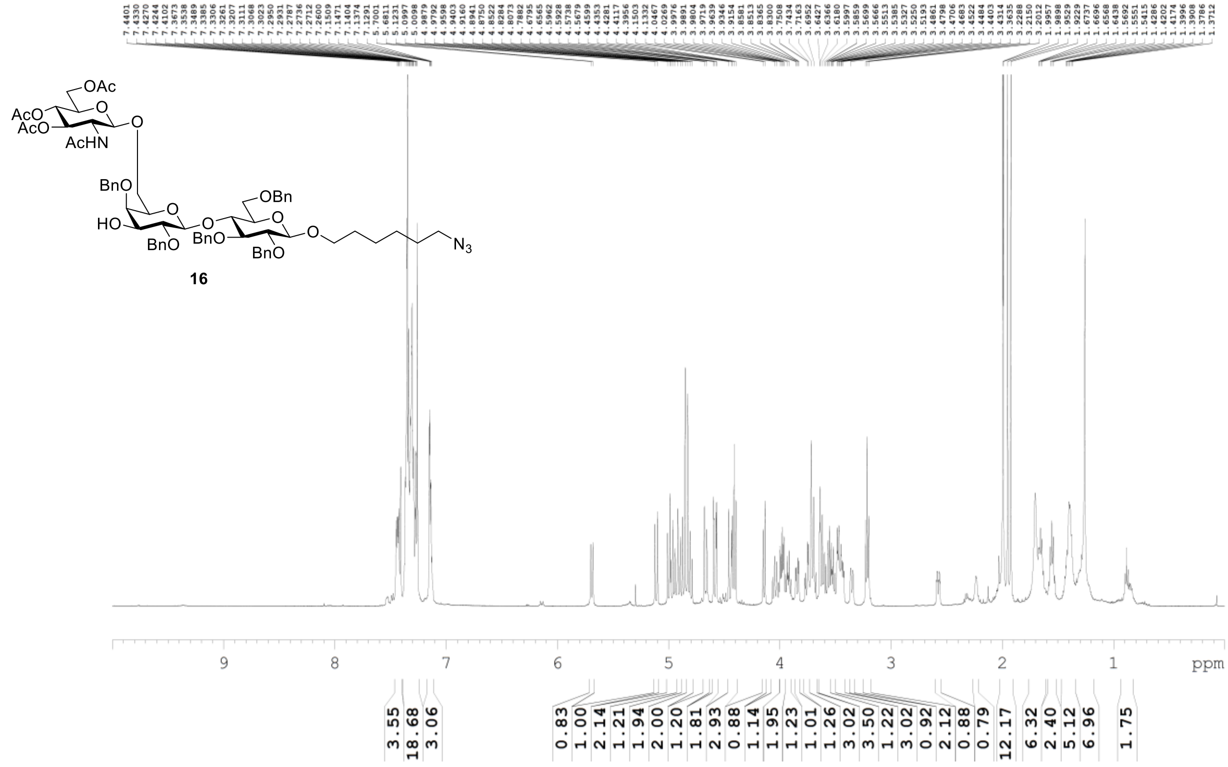

^{1}H NMR spectrum of **16**, recorded at 500 MHz in $CDCl_3$ (δ = 7.26 ppm).

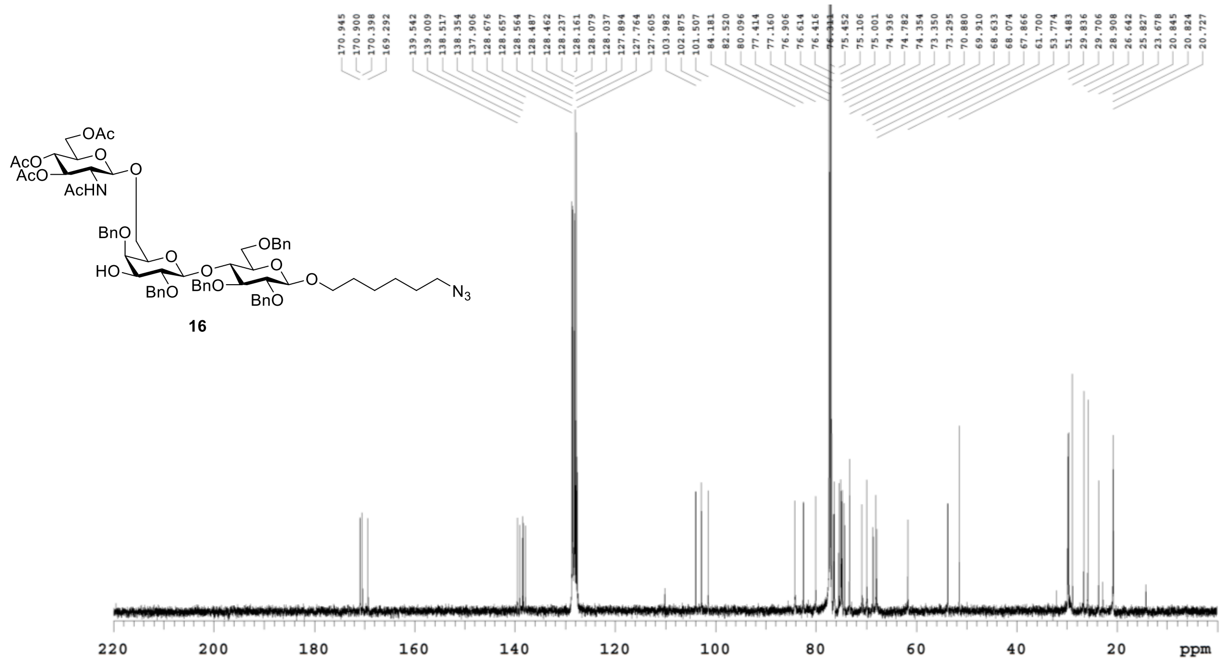

^{13}C NMR spectrum of **16**, recorded at 125 MHz in $CDCl_3$ (δ = 77.16 ppm).

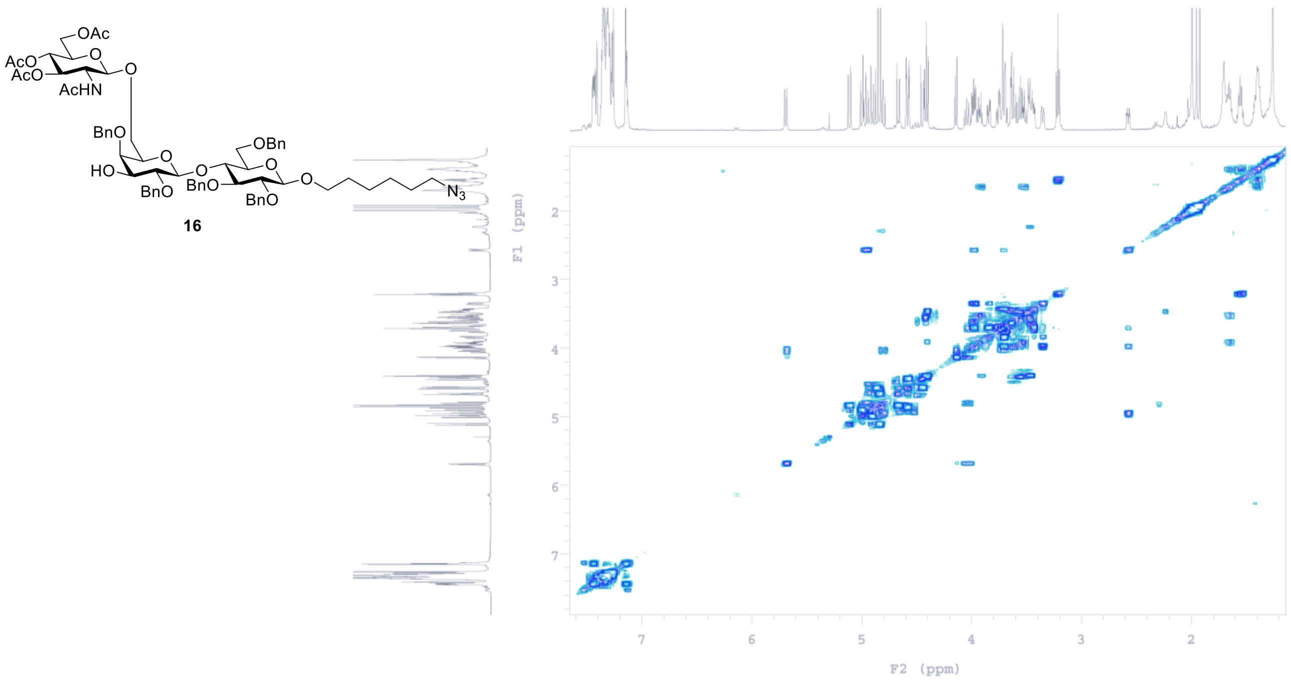

COSY NMR spectrum of **16**, recorded at 500/125 MHz in $CDCl_3$.

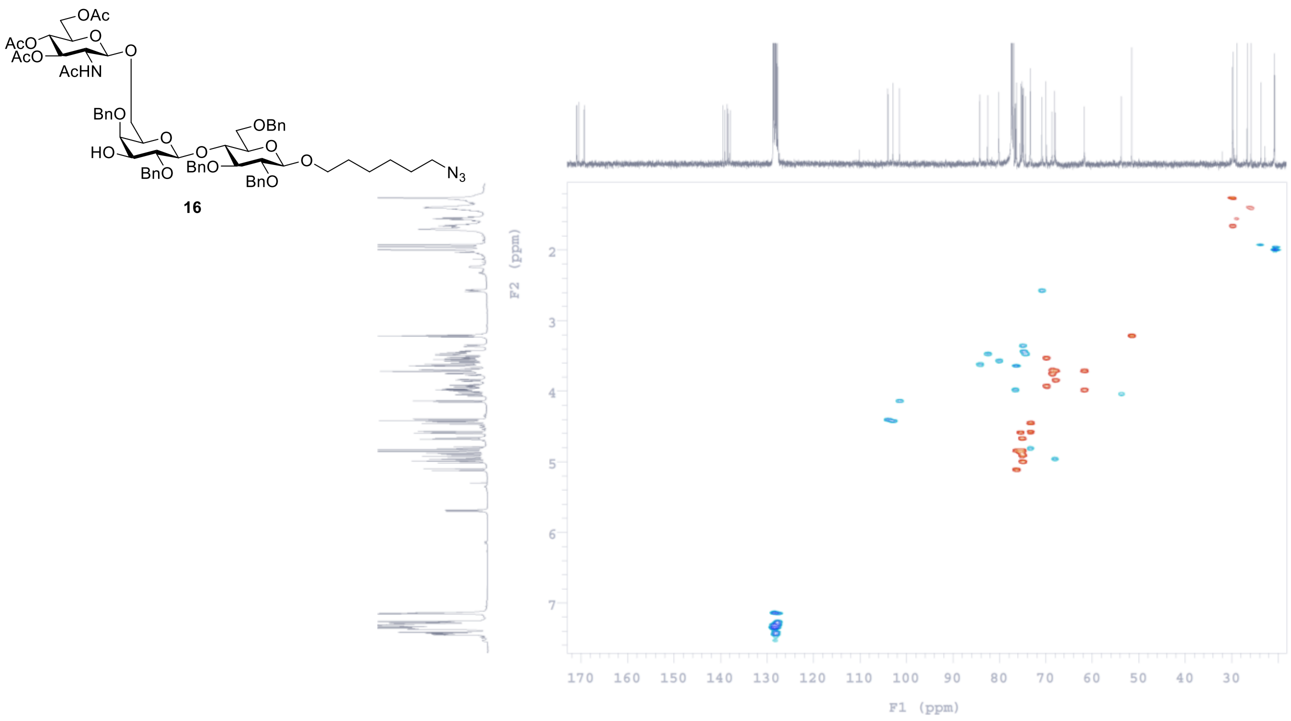

HSQC NMR spectrum of **16**, recorded at 500/125 MHz in $CDCl_3$.

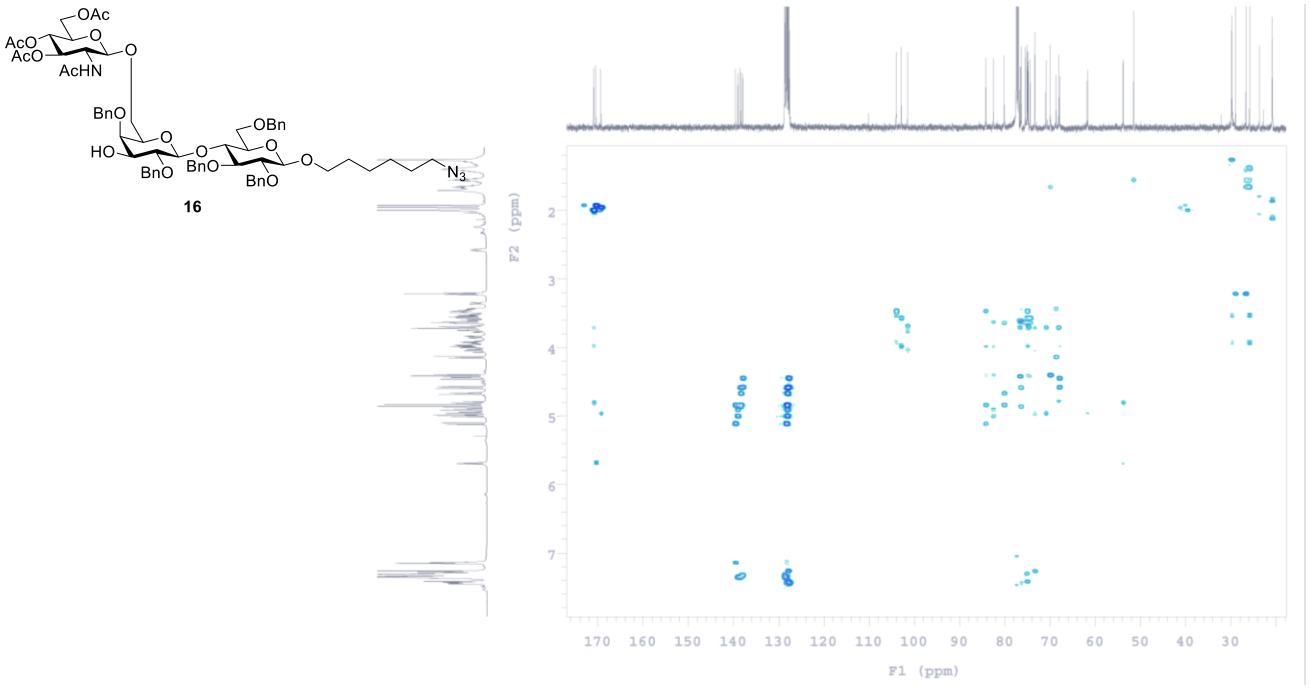

HMBC NMR spectrum of **16**, recorded at 500/125 MHz in $CDCl_3$.

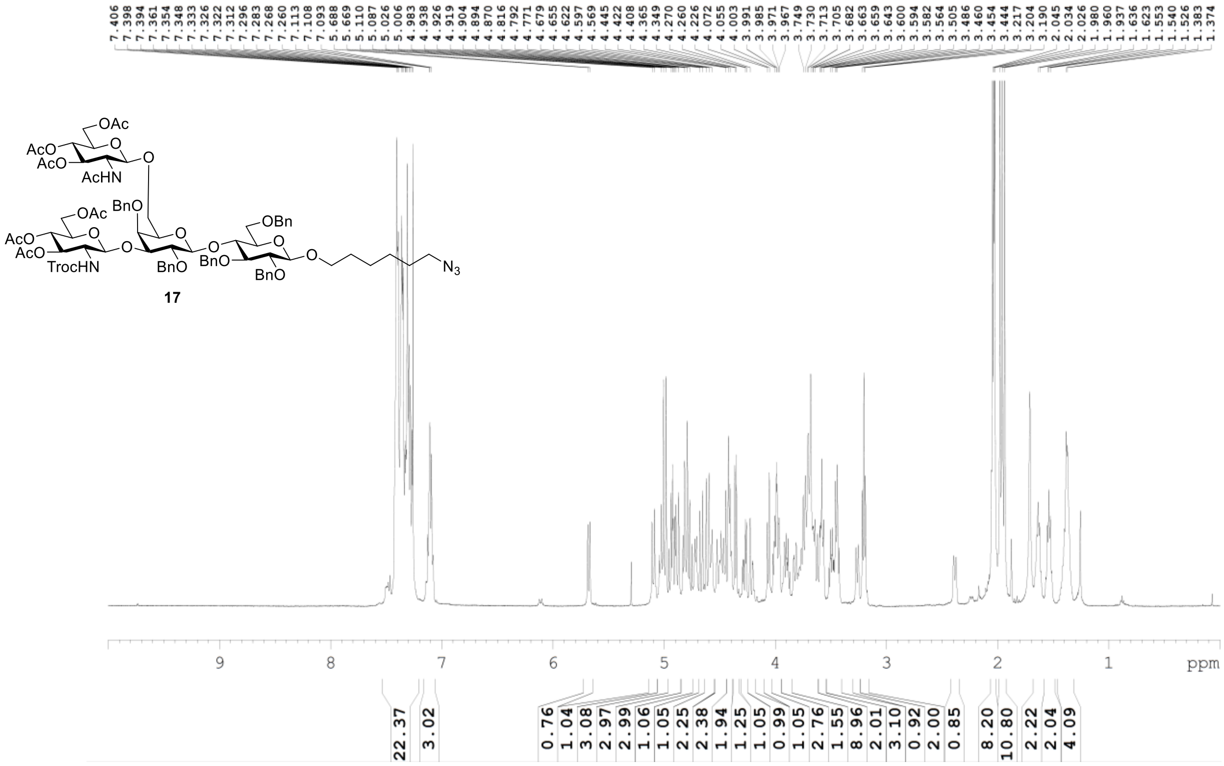

^{1}H NMR spectrum of **17**, recorded at 500 MHz in $CDCl_3$ (δ = 7.26 ppm).

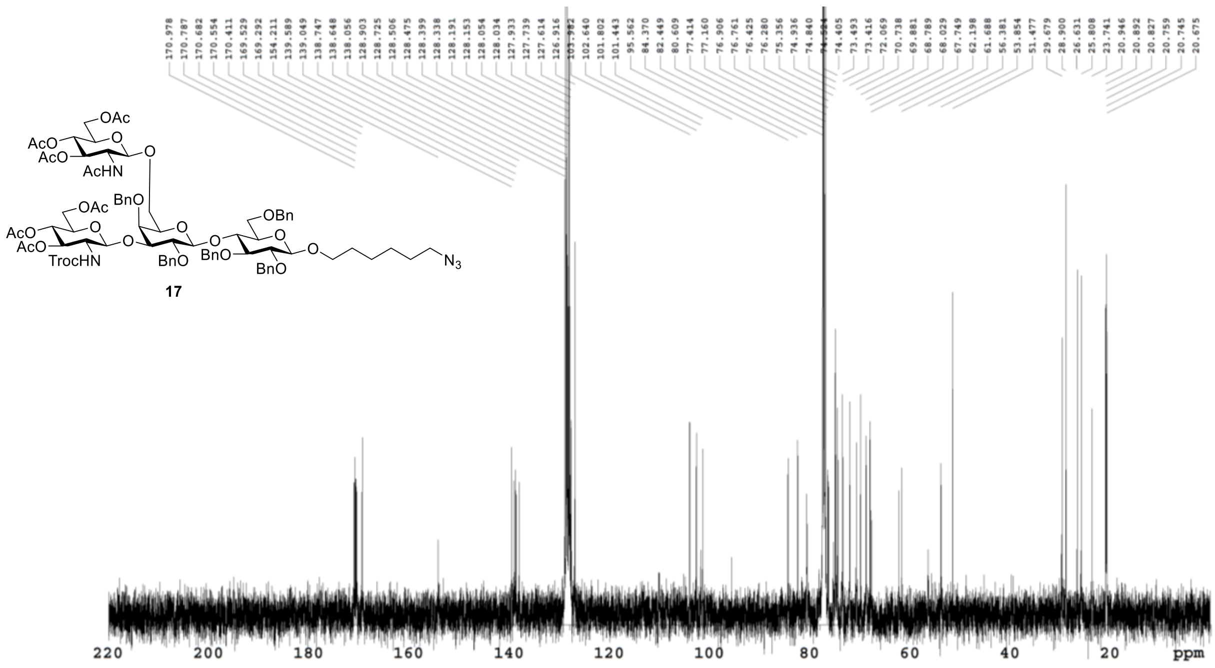

^{13}C NMR spectrum of **17**, recorded at 125 MHz in $CDCl_3$ (δ = 77.16 ppm).

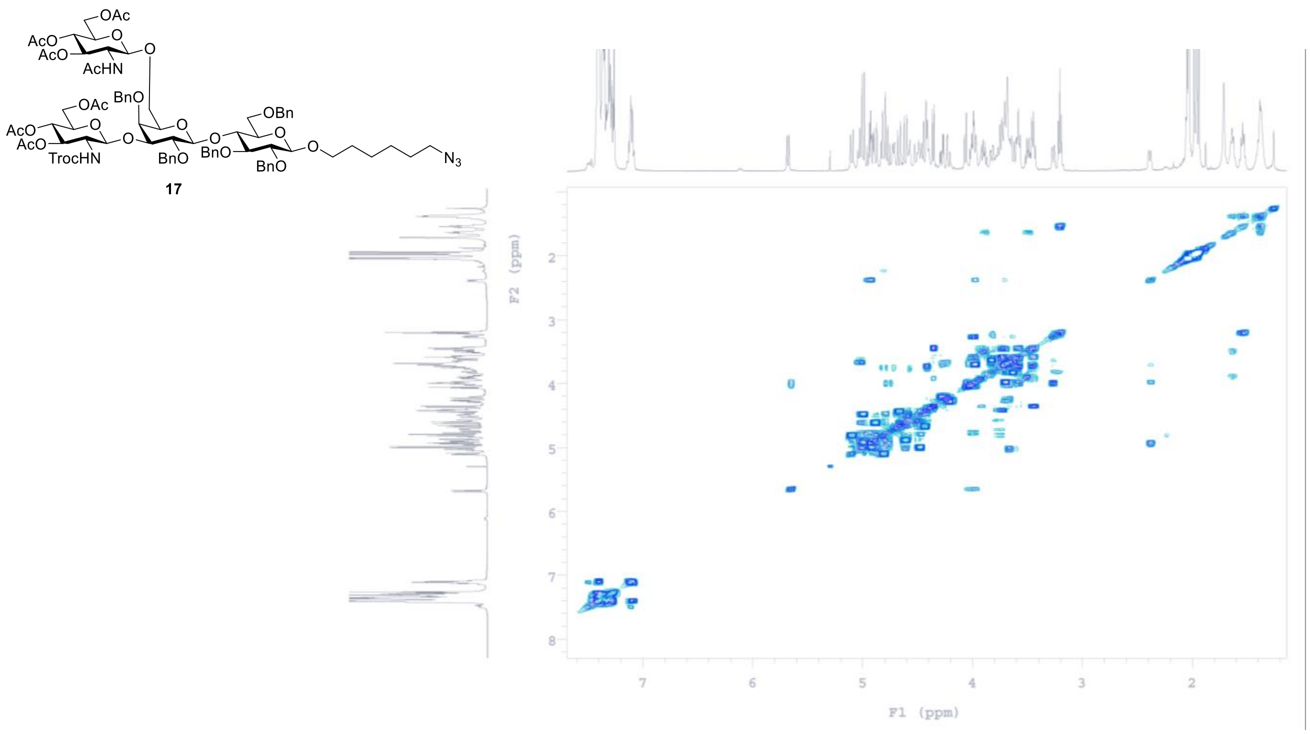

COSY NMR spectrum of **17**, recorded at 500/125 MHz in $CDCl_3$.

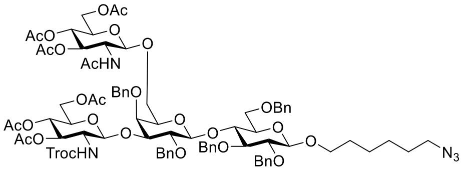

17

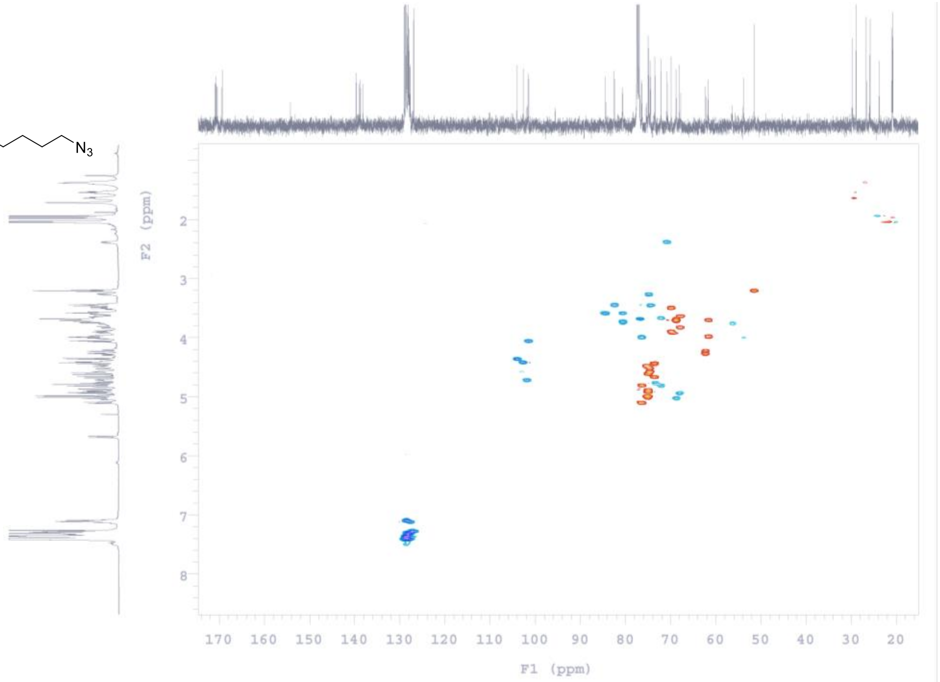

HSQC NMR spectrum of **17**, recorded at 500/125 MHz in $CDCl_3$.

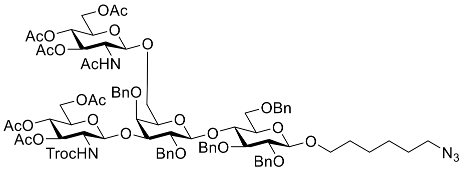

17

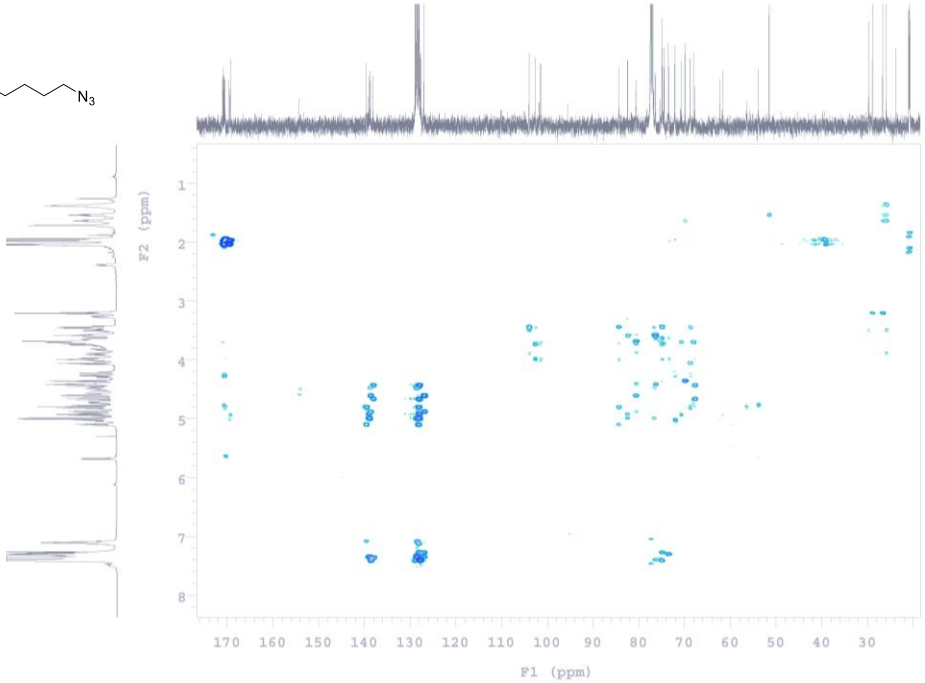

HMBC NMR spectrum of **17**, recorded at 500/125 MHz in $CDCl_3$.

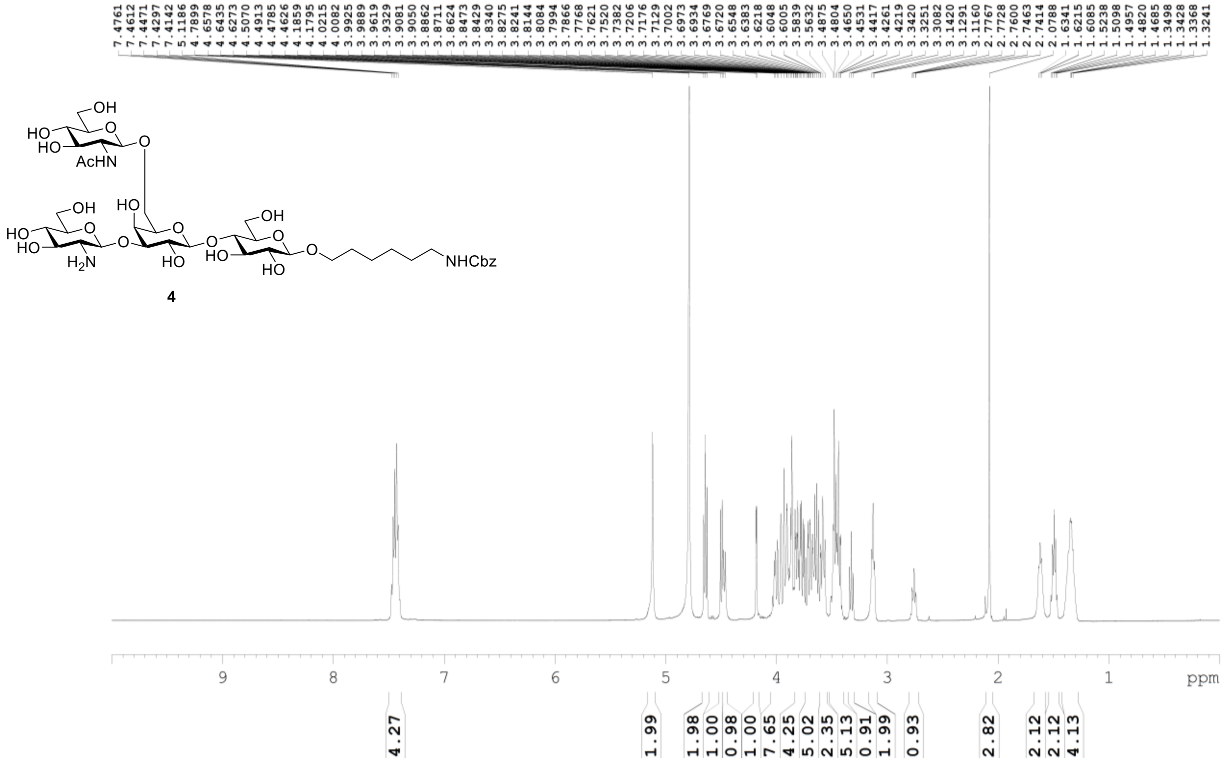

^{1}H NMR spectrum of **4**, recorded at 500 MHz in D_2O. Residual water signal was used as reference (δ = 4.79 ppm).

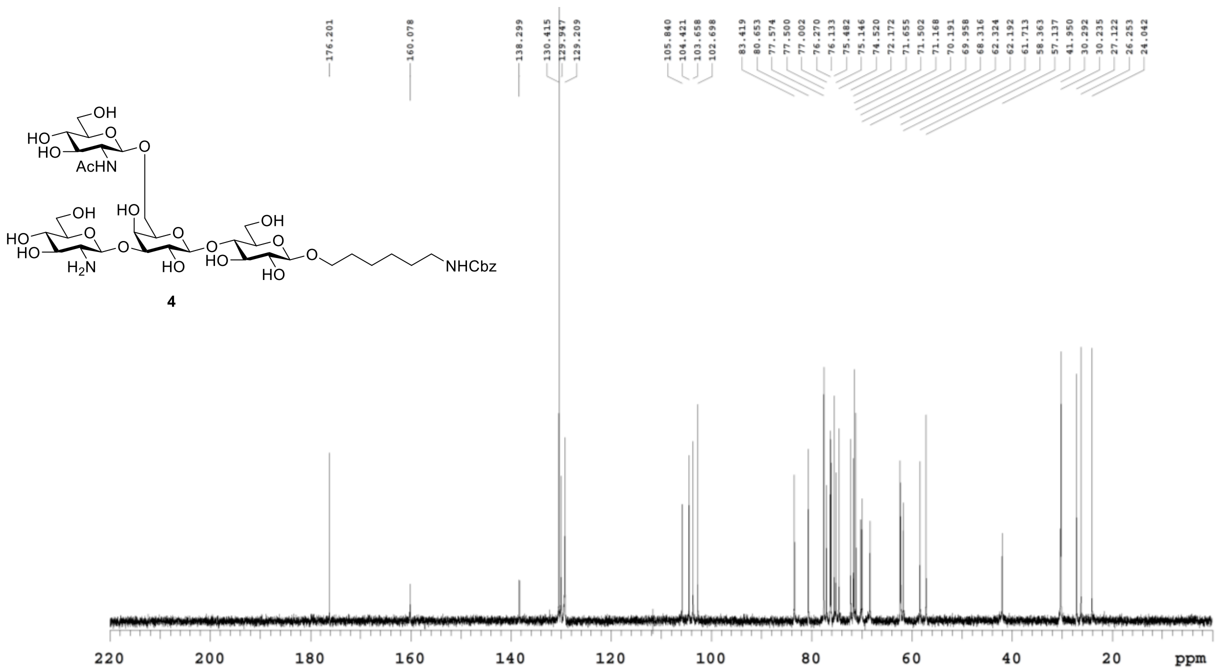

^{13}C NMR spectrum of **4**, recorded at 125 MHz in D_2O.

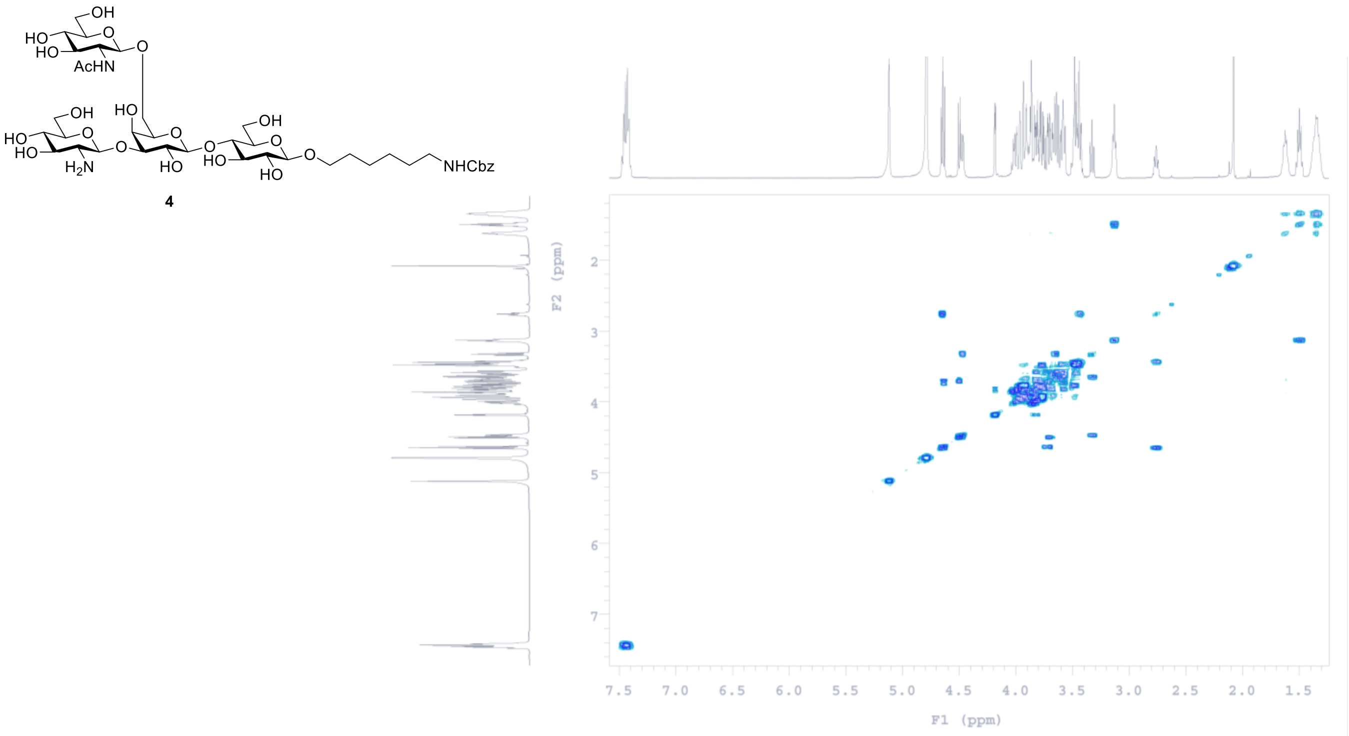

COSY NMR spectrum of **4**, recorded at 500/125 MHz in D_2O.

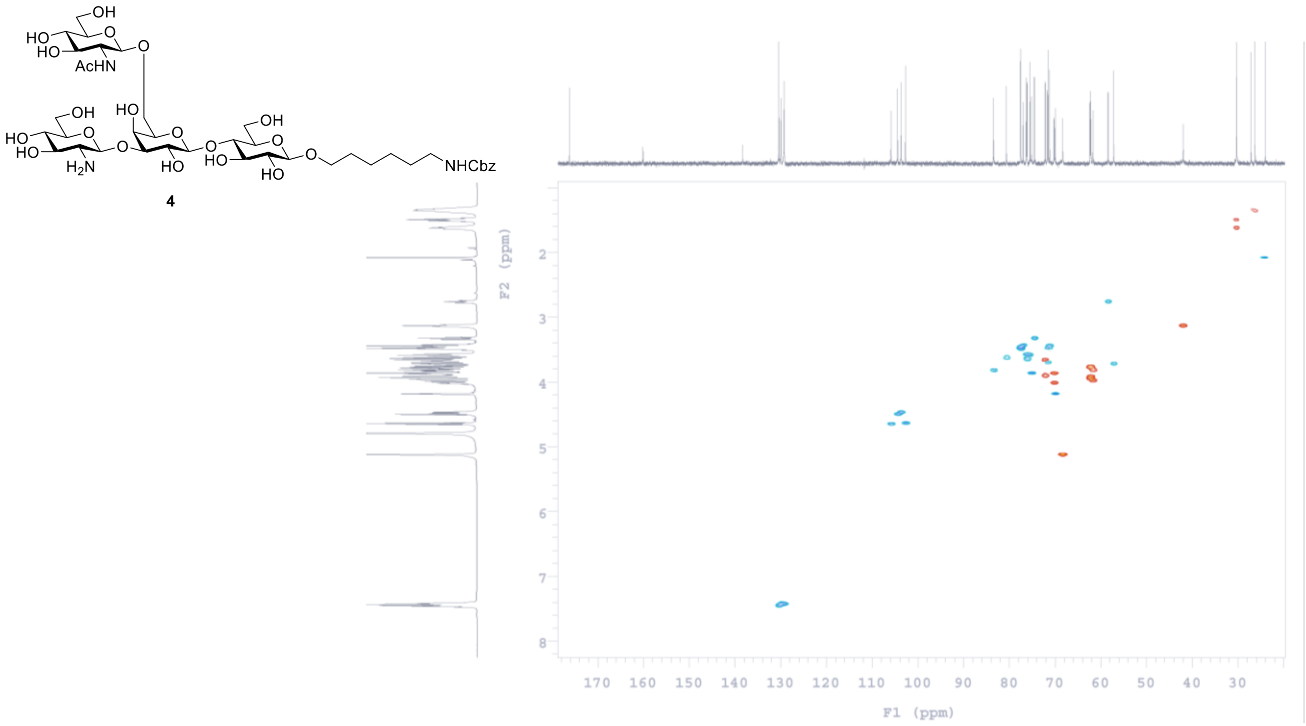

HSQC NMR spectrum of **4**, recorded at 500/125 MHz in D_2O.

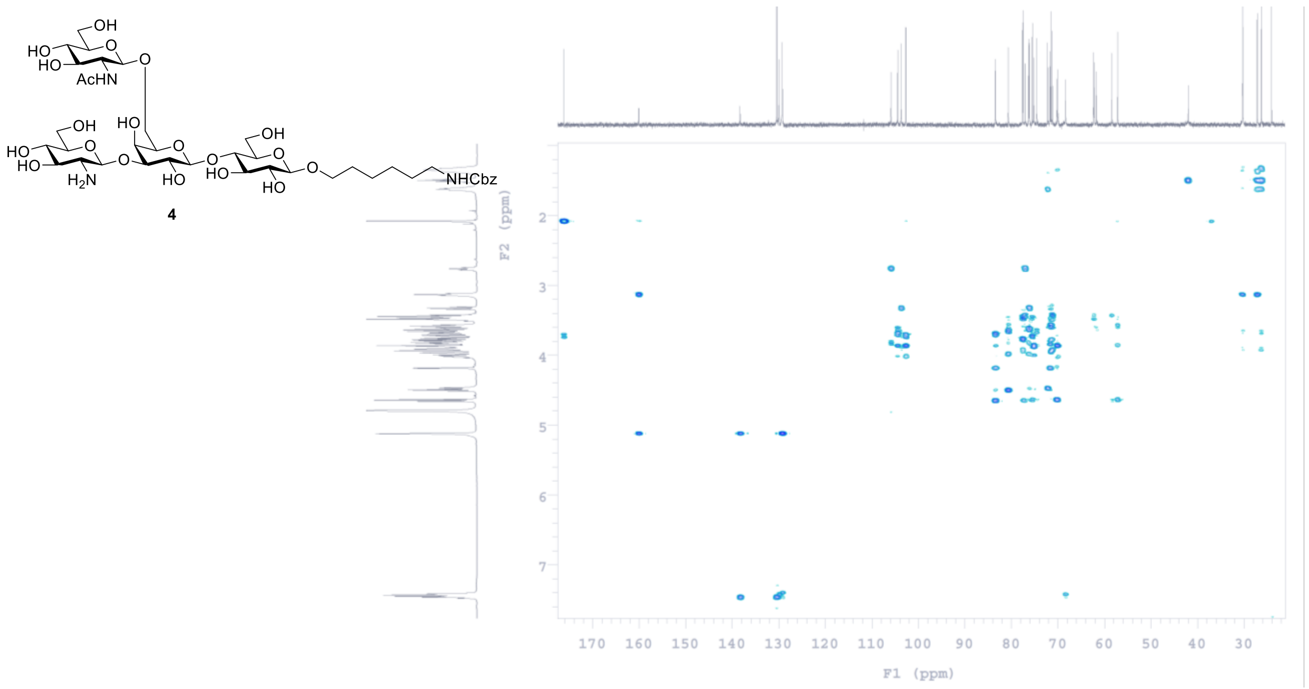

HMBC NMR spectrum of **4**, recorded at 500/125 MHz in D_2O.

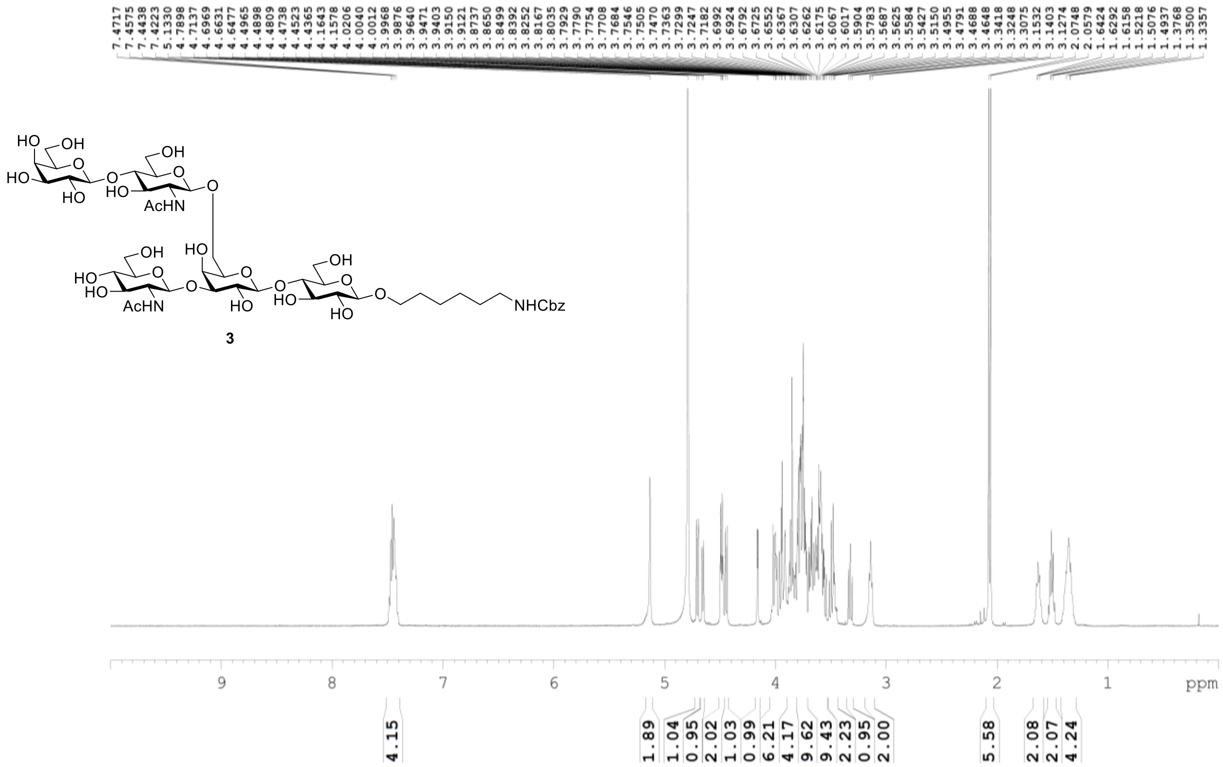

^{1}H NMR spectrum of **3**, recorded at 500 MHz in D_2O. Residual water signal was used as reference (δ = 4.79 ppm).

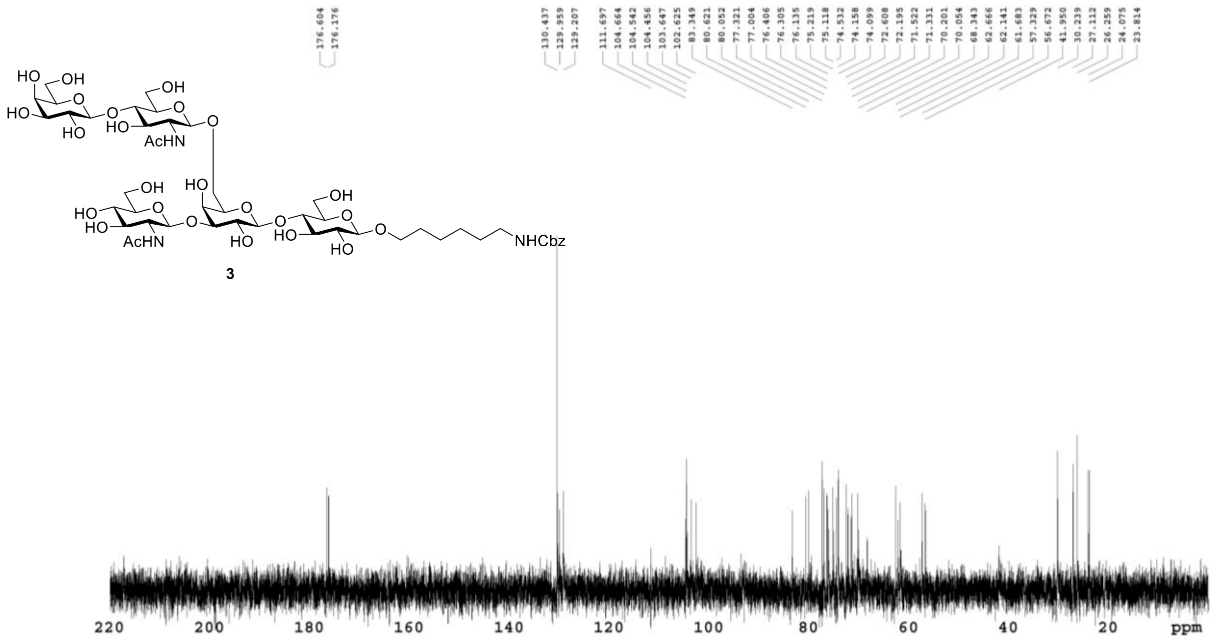

^{13}C NMR spectrum of **3**, recorded at 125 MHz in D_2O.

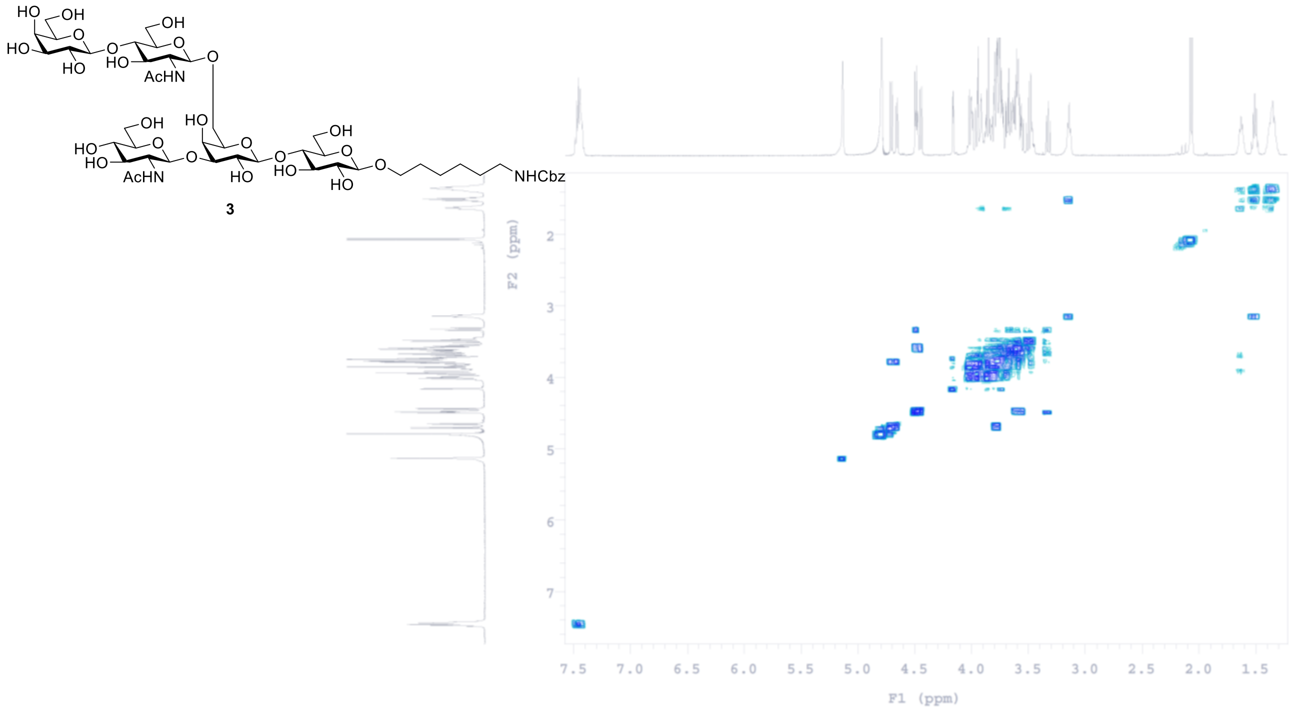

COSY NMR spectrum of **3**, recorded at 500/125 MHz in D_2O.

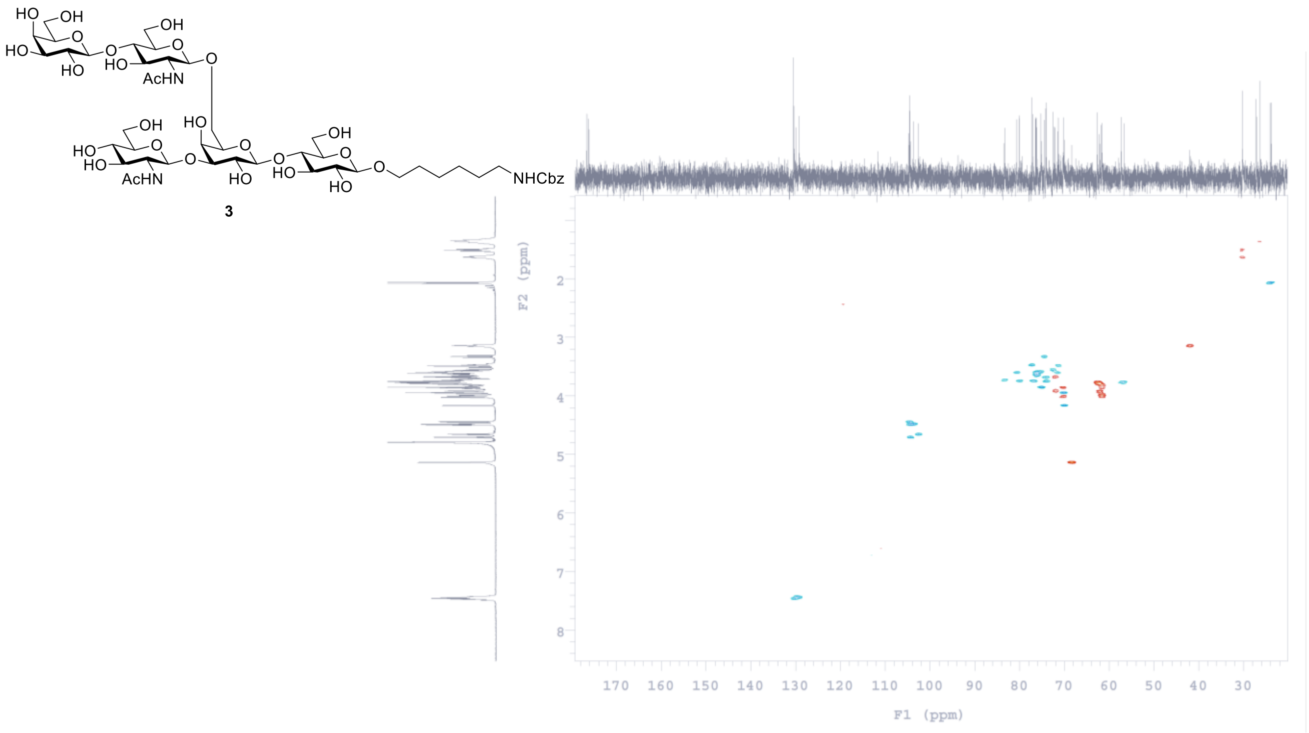

HSQC NMR spectrum of **3**, recorded at 500/125 MHz in D_2O.

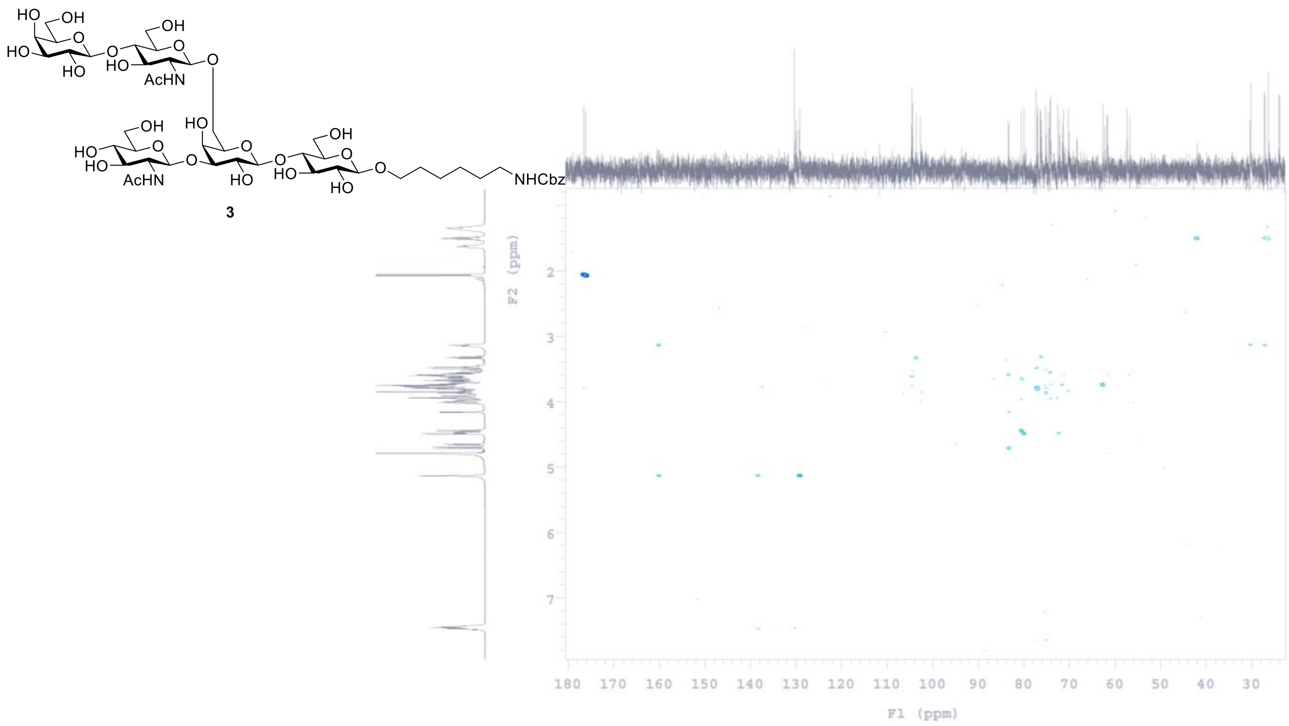

HMBC NMR spectrum of **3**, recorded at 500/125 MHz in D_2O.

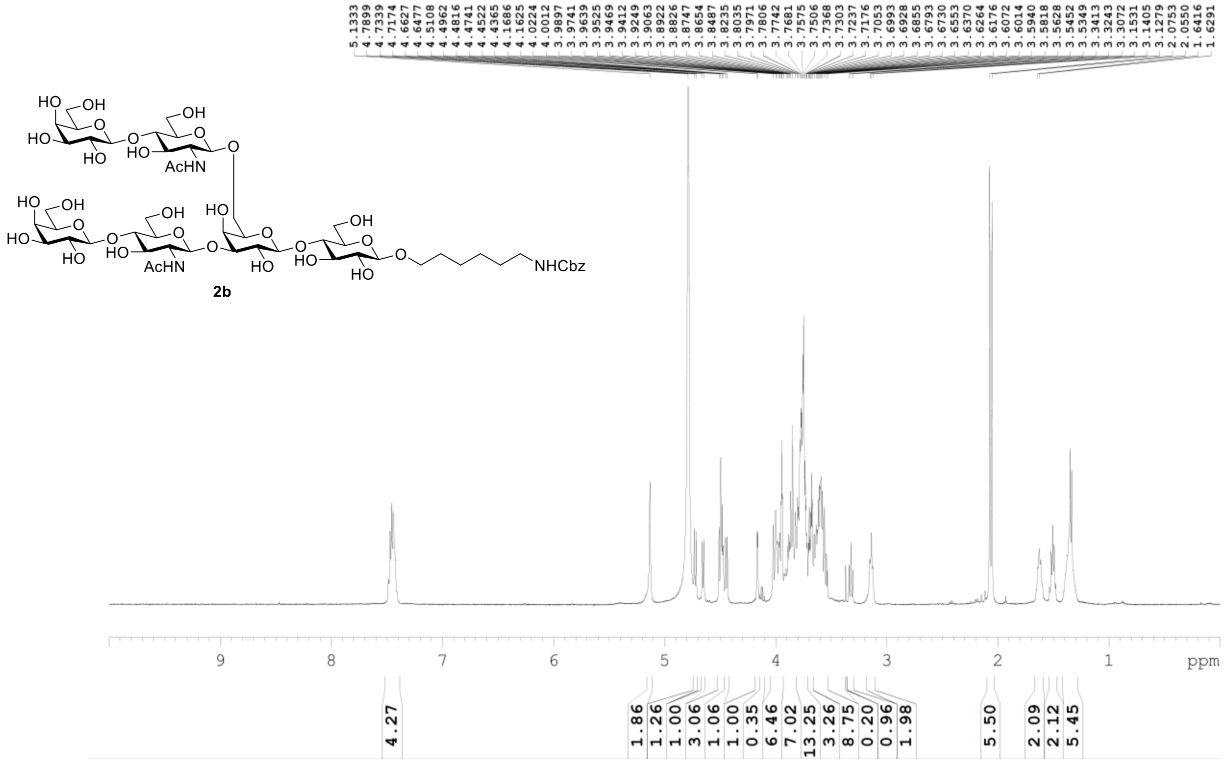

^{1}H NMR spectrum of **2b**, recorded at 500 MHz in D_2O. Residual water signal was used as reference (δ = 4.79 ppm).

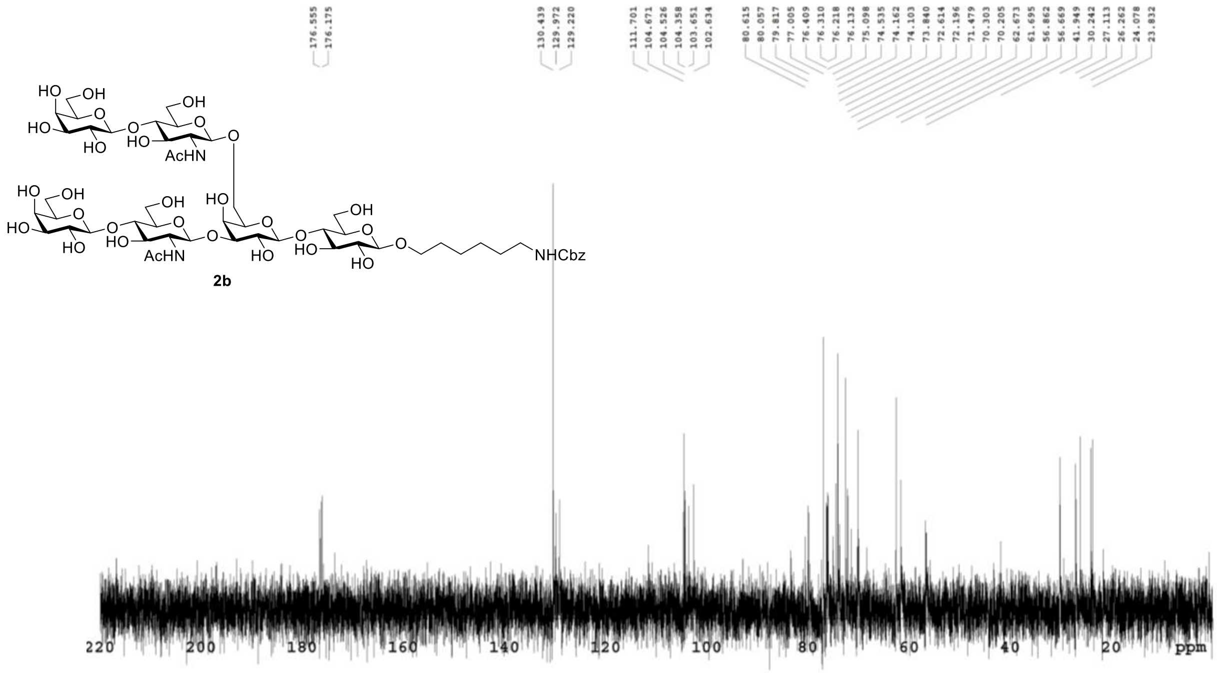

^{13}C NMR spectrum of **2b**, recorded at 125 MHz in D_2O.

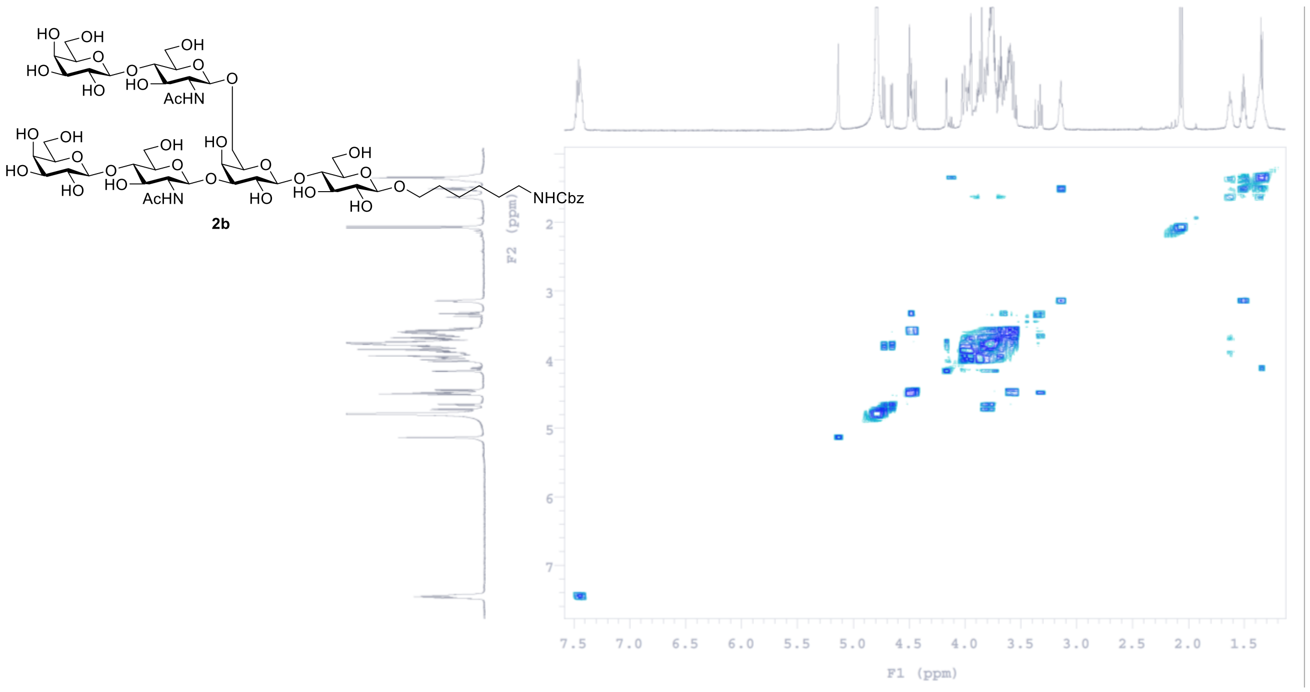

COSY NMR spectrum of **2b**, recorded at 500/125 MHz in D_2O.

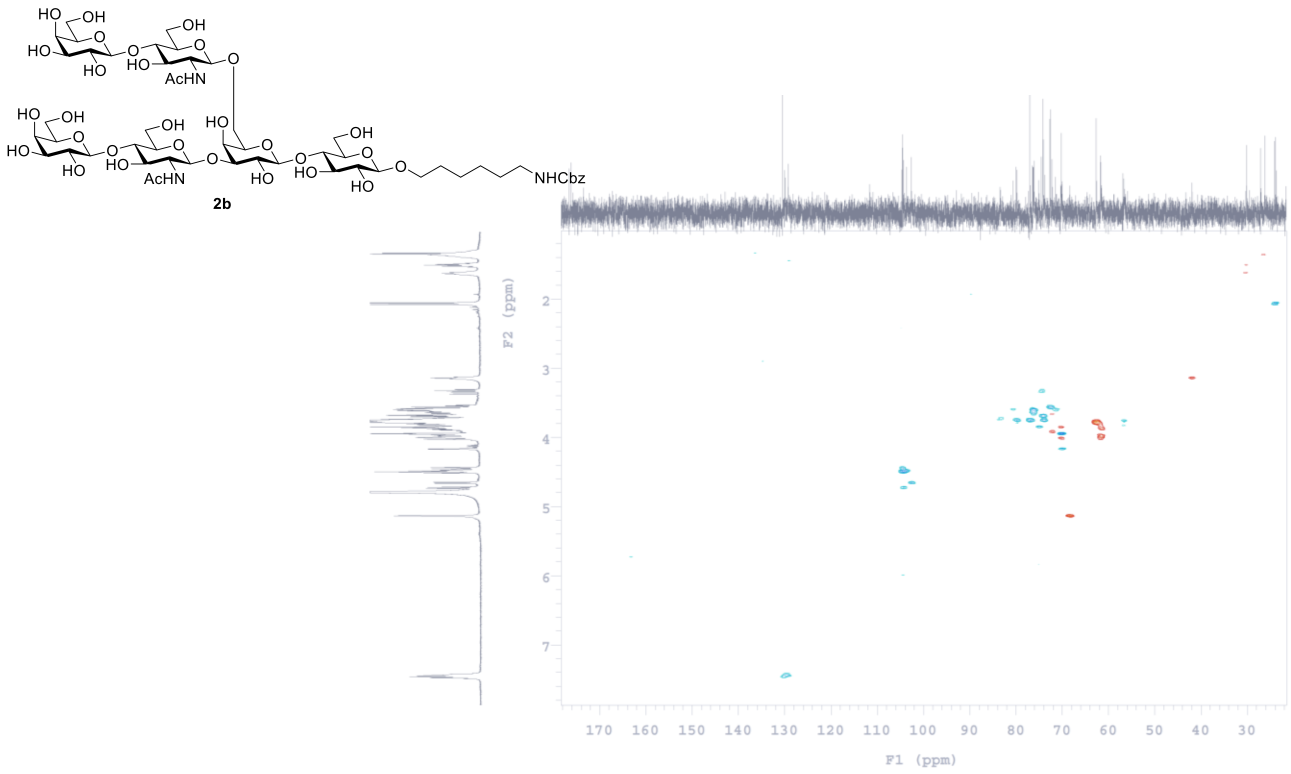

HSQC NMR spectrum of **2b**, recorded at 500/125 MHz in D_2O.

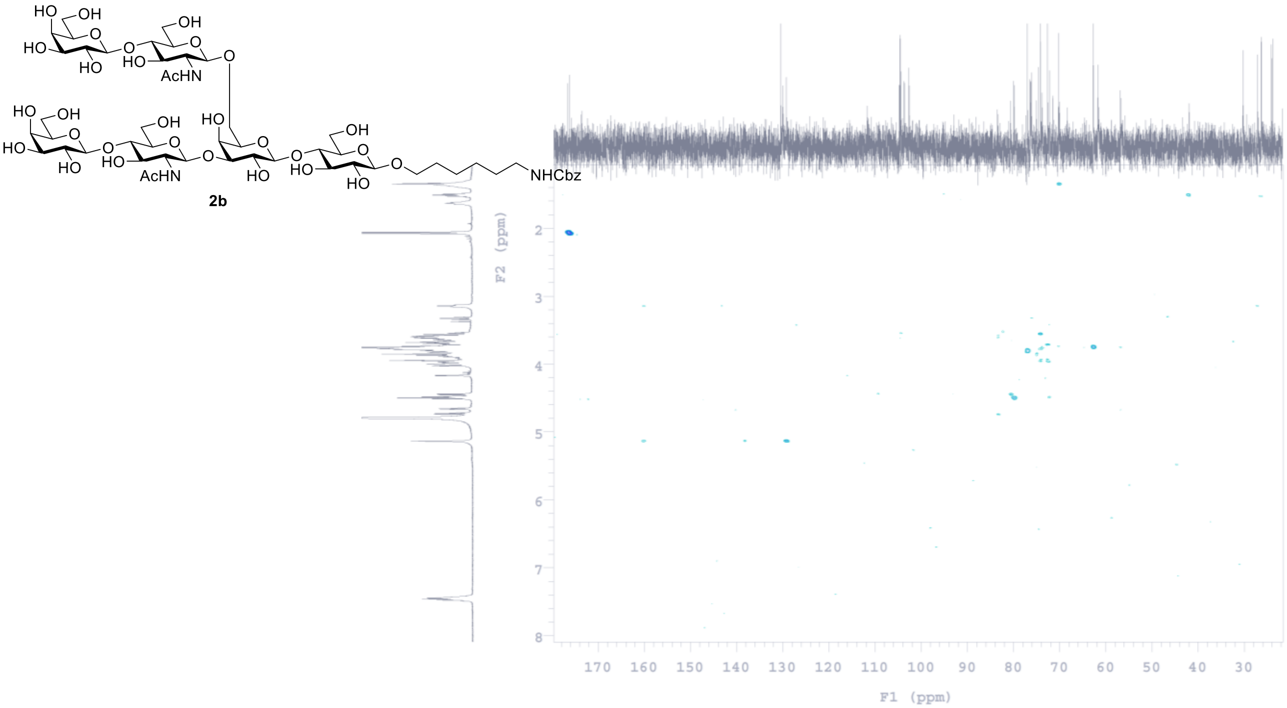

HMBC NMR spectrum of **2b**, recorded at 500/125 MHz in D_2O.

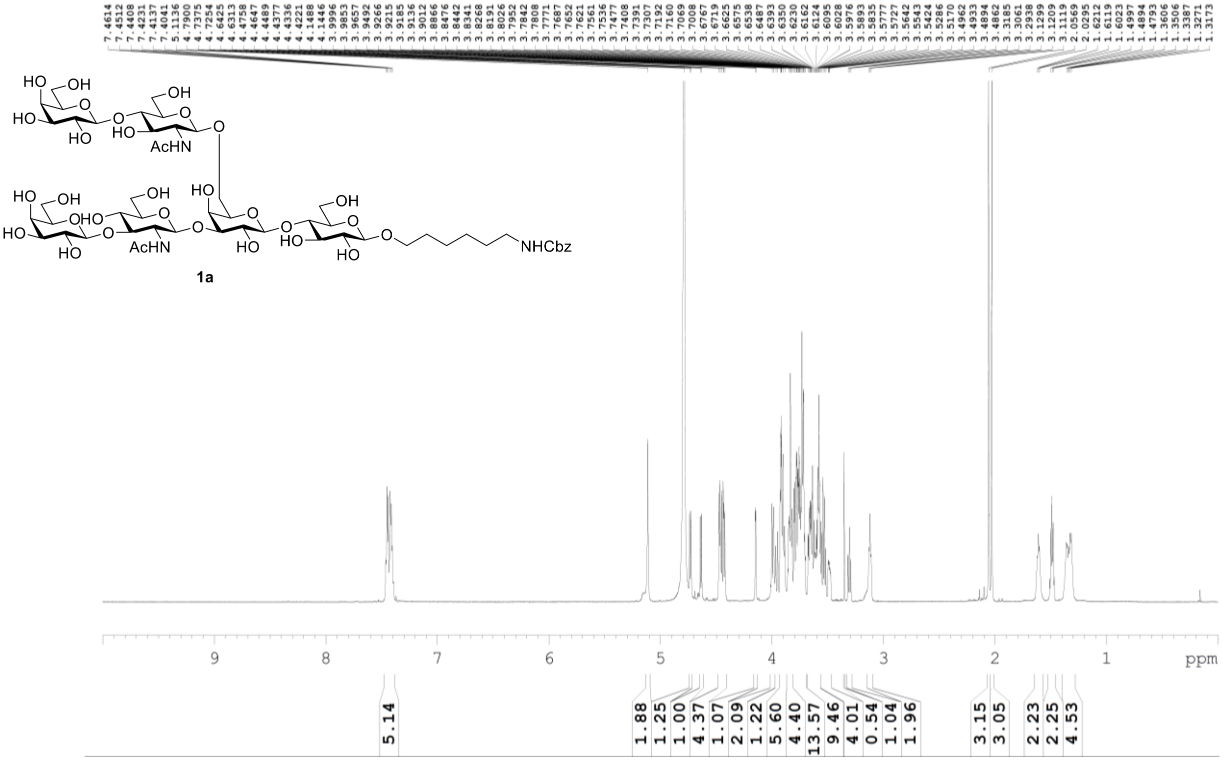

^{1}H NMR spectrum of **1a**, recorded at 700 MHz in D_2O. Residual water signal was used as reference (δ = 4.79 ppm).

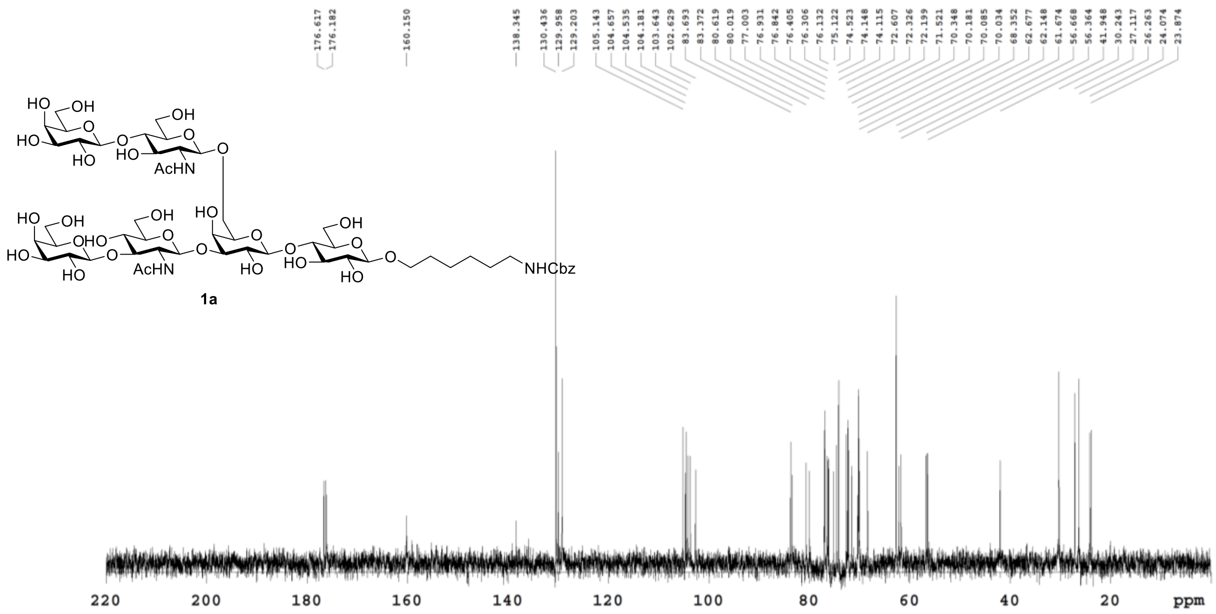

^{13}C NMR spectrum of **1a**, recorded at 175 MHz in D_2O.

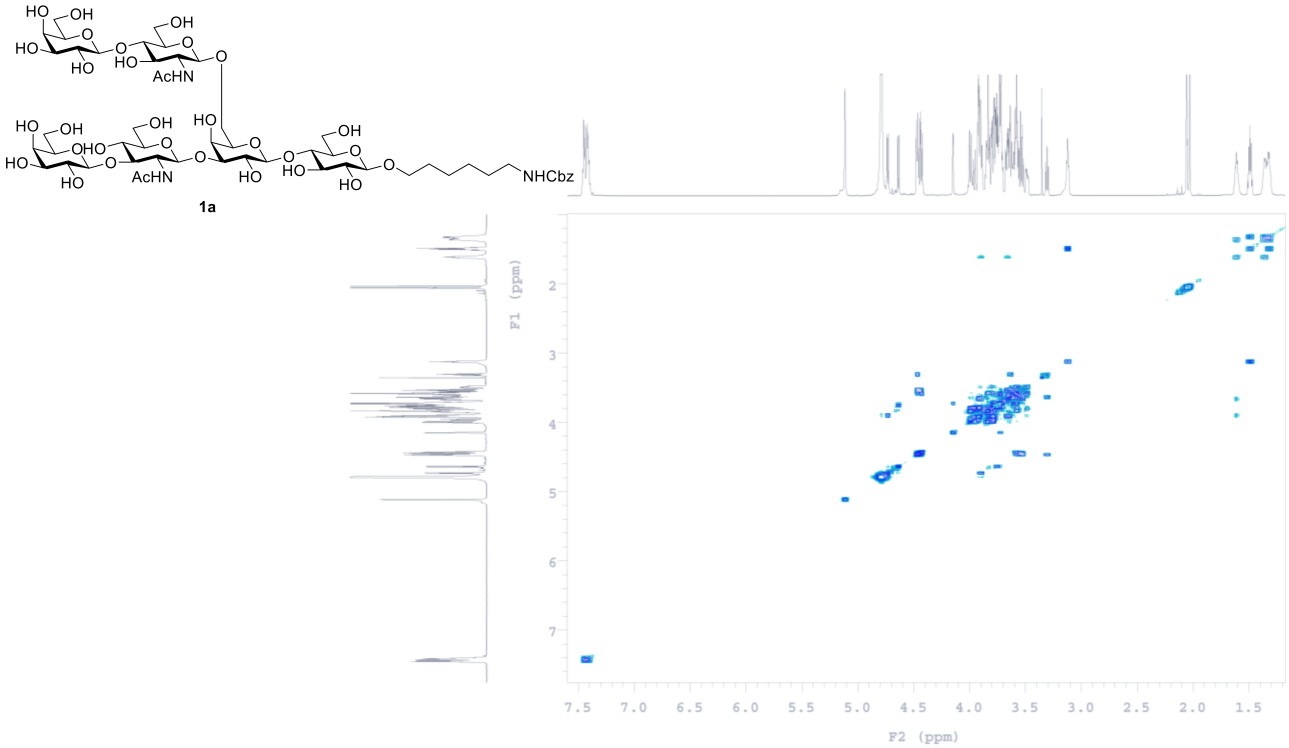

COSY NMR spectrum of **1a**, recorded at 700/175 MHz in D_2O.

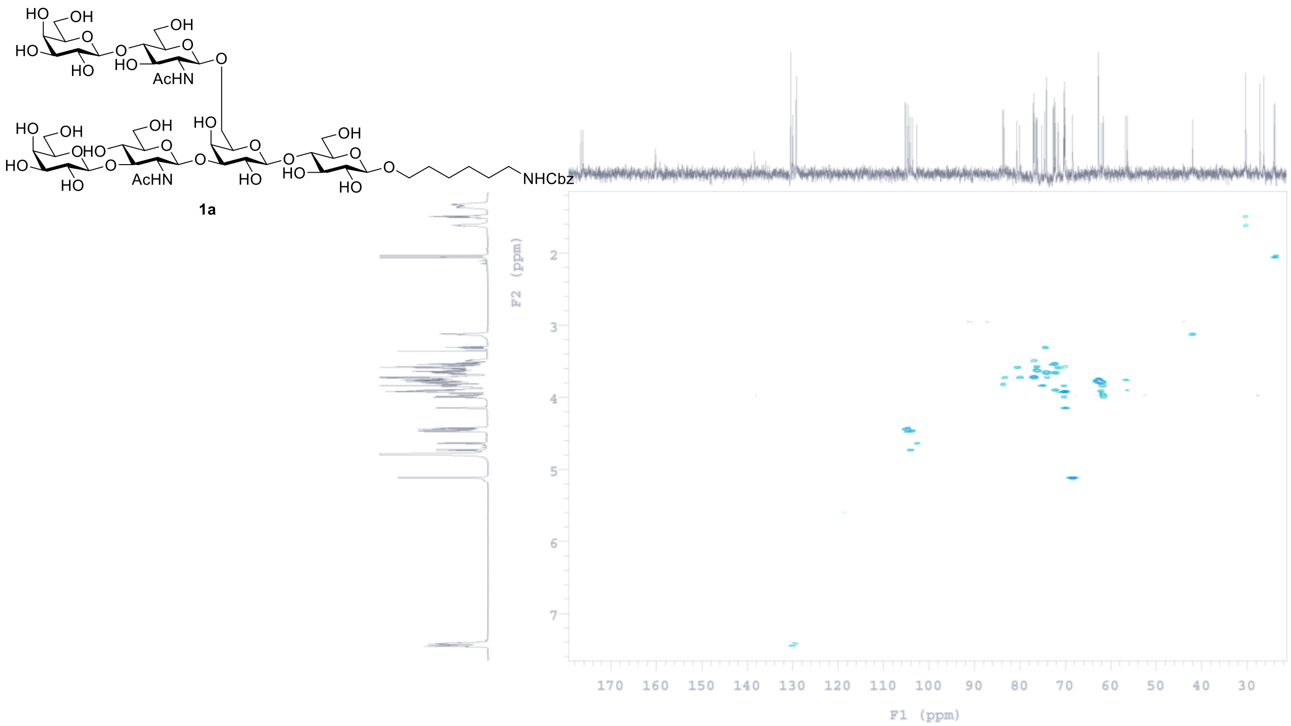

HSQC NMR spectrum of **1a**, recorded at 700/175 MHz in D_2O.

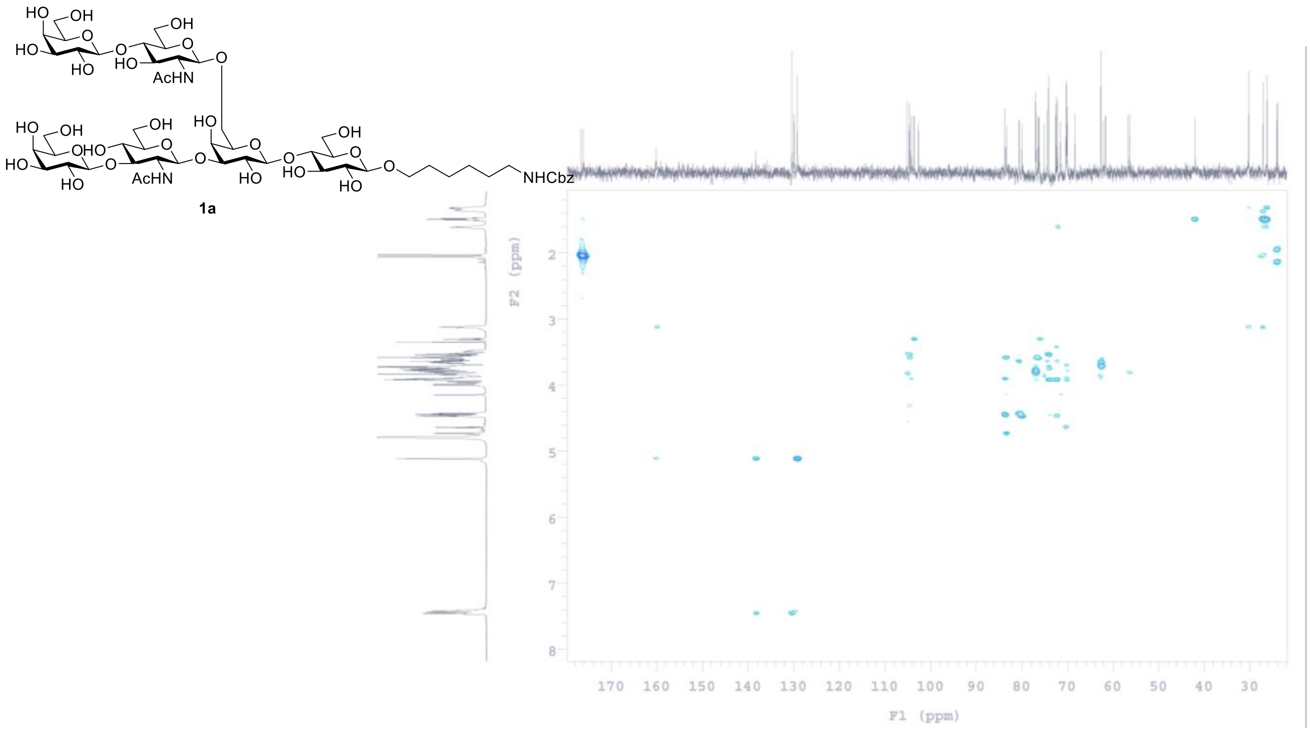

HMBC NMR spectrum of **1a**, recorded at 700/175 MHz in D_2O.

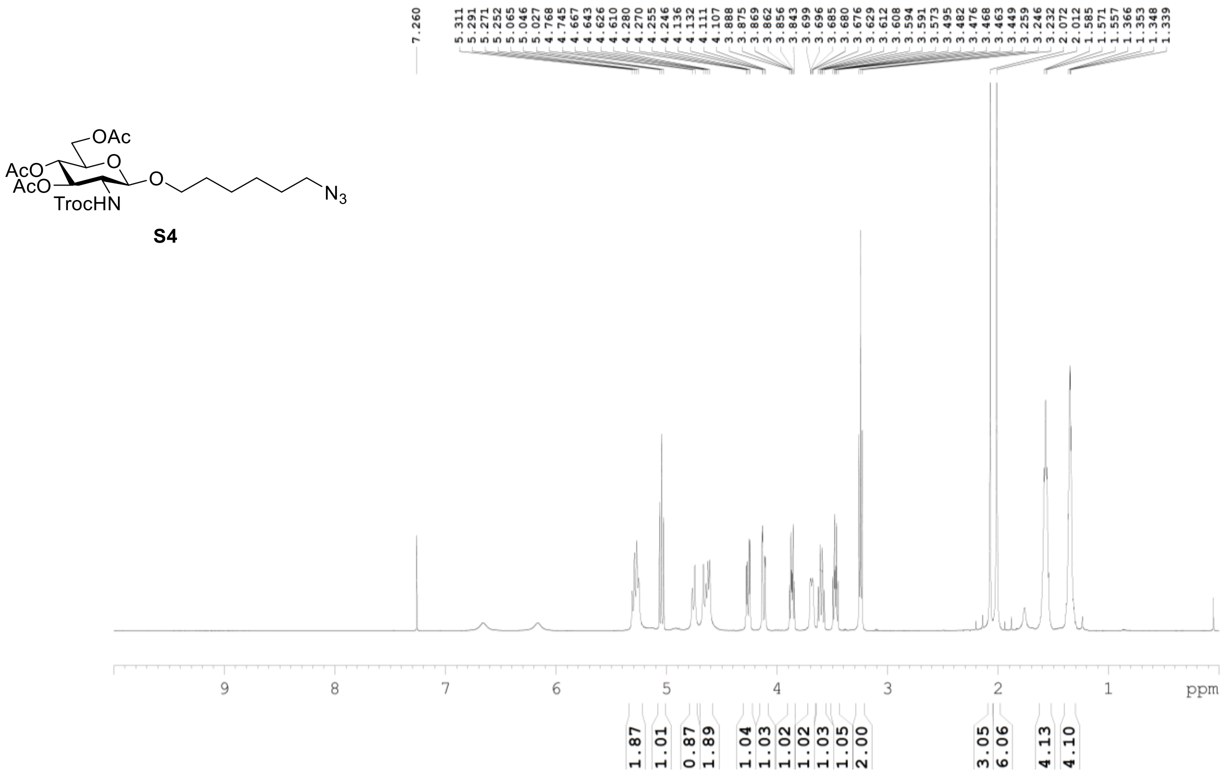

^{1}H NMR spectrum of **S4**, recorded at 500 MHz in $CDCl_3$ (δ = 7.26 ppm).

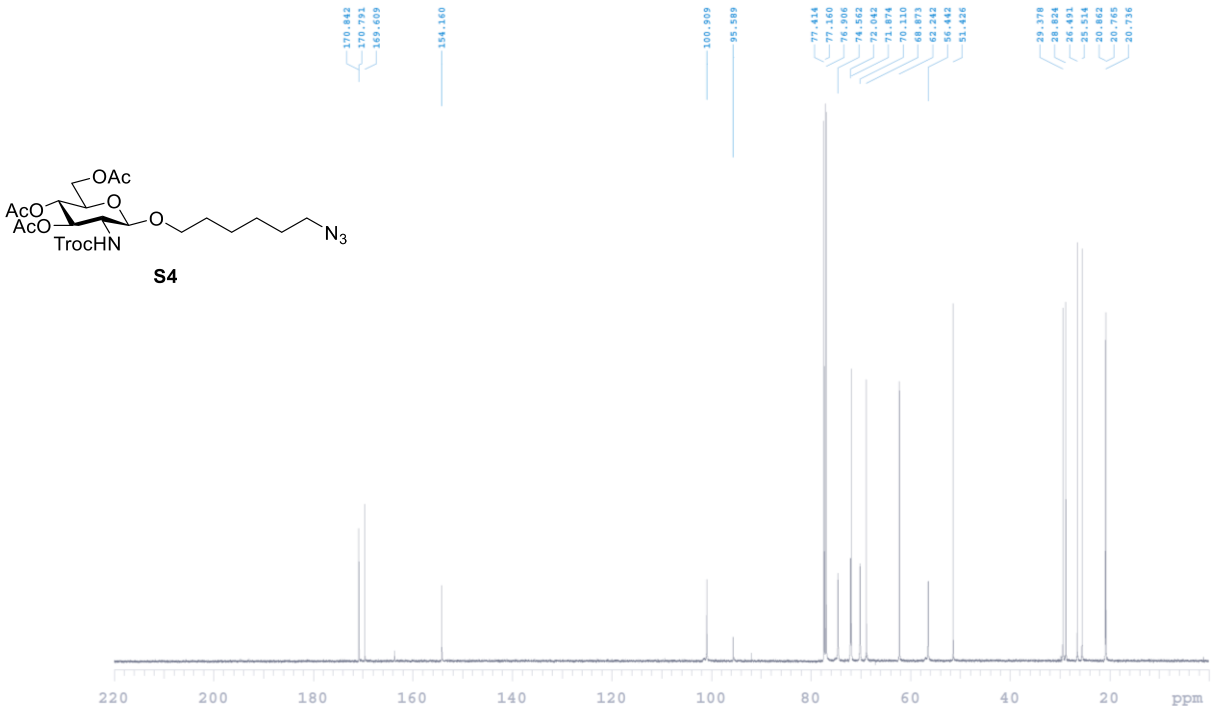

^{13}C NMR spectrum of **S4**, recorded at 125 MHz in $CDCl_3$ (δ = 77.16 ppm).

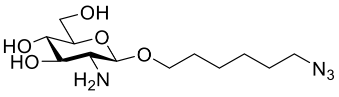

S2

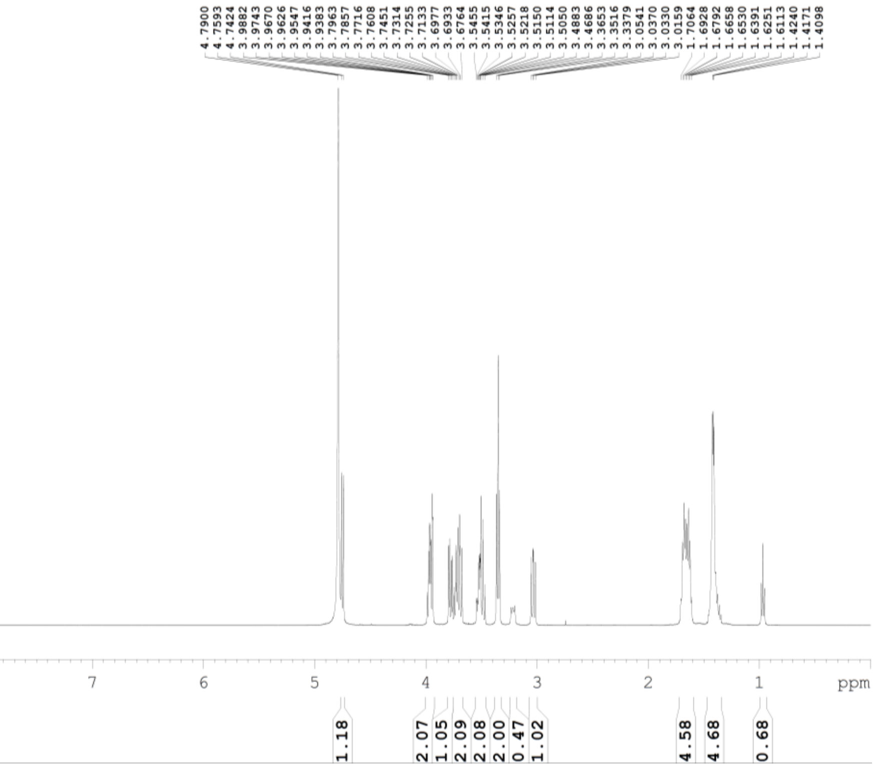

^{1}H NMR spectrum of **S2**, recorded at 500 MHz in D_2O. Residual water signal was used as reference (δ = 4.79 ppm).

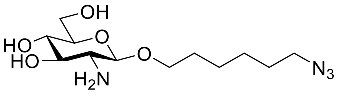

S2

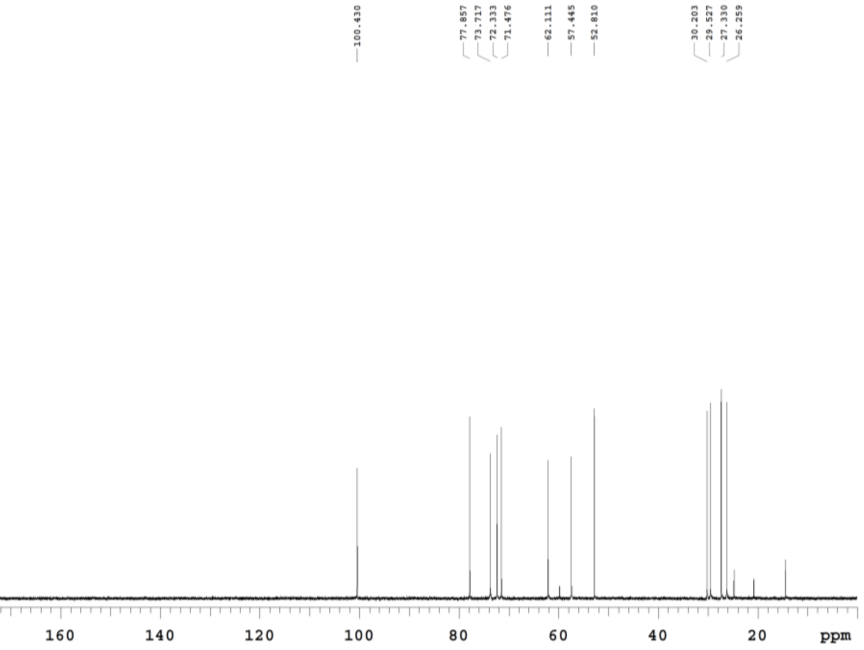

^{13}C NMR spectrum of **S2**, recorded at 125 MHz in D_2O.

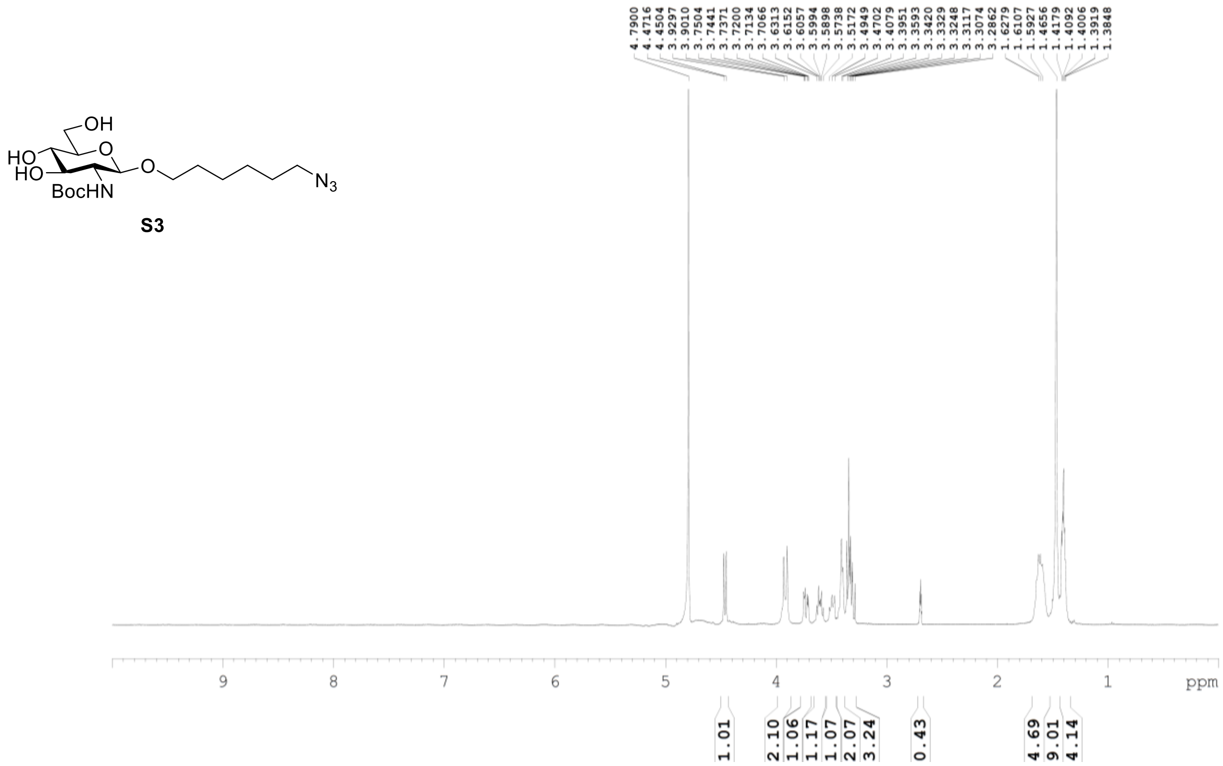

^{1}H NMR spectrum of **S3**, recorded at 400 MHz in D_2O and DMSO-d_6. Residual water signal was used as reference (δ = 4.79 ppm).

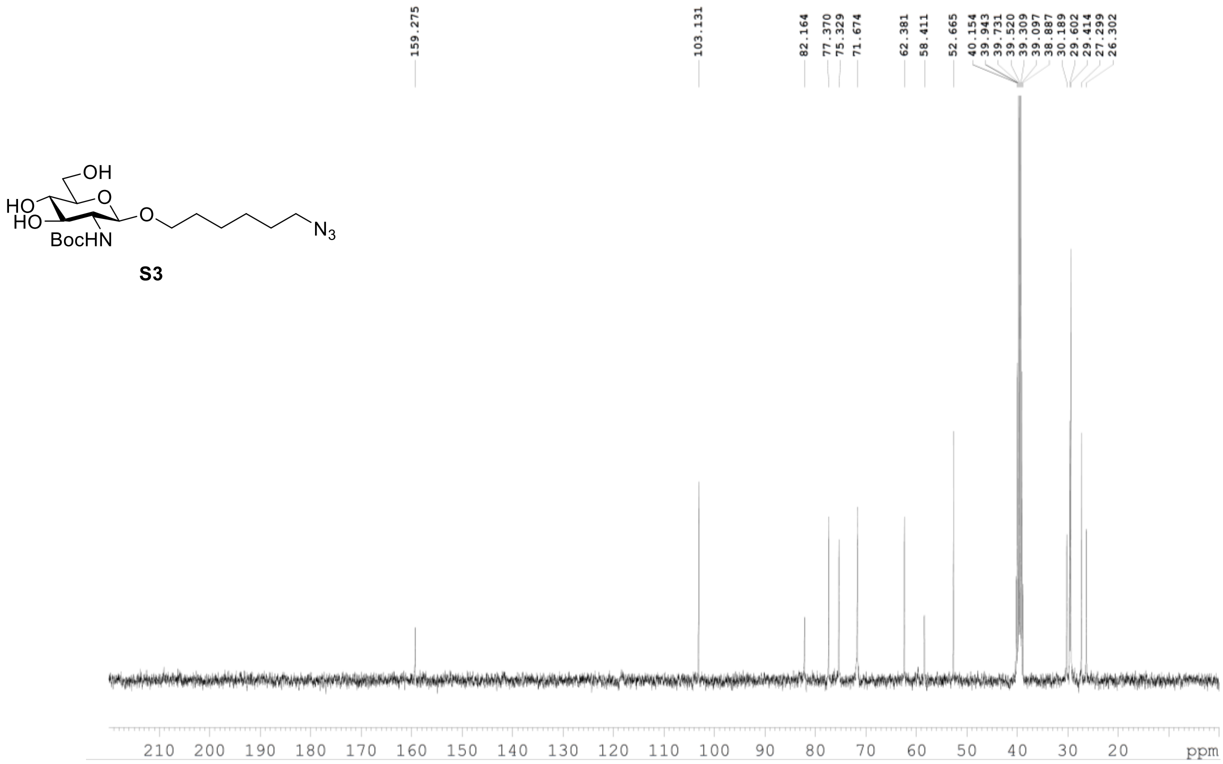

^{13}C NMR spectrum of **S3**, recorded at 100 MHz in D_2O and DMSO-d_6 (δ = 39.52 ppm as reference).
